# Conformational rearrangements enable iterative backbone *N*-methylation in RiPP biosynthesis

**DOI:** 10.1038/s41467-021-25575-7

**Published:** 2021-09-09

**Authors:** Fredarla S. Miller, Kathryn K. Crone, Matthew R. Jensen, Sudipta Shaw, William R. Harcombe, Mikael H. Elias, Michael F. Freeman

**Affiliations:** 1 Department of Biochemistry, Molecular Biology, and Biophysics, University of Minnesota-Twin Cities, St. Paul, MN USA; 2 BioTechnology Institute, University of Minnesota-Twin Cities, St. Paul, MN USA; 3 Department of Ecology, Evolution and Behavior, University of Minnesota-Twin Cities, St. Paul, MN USA; 4 Present Address: Science Department, Concordia University-St. Paul, St. Paul, MN USA

**Keywords:** Peptides, Natural products, X-ray crystallography, Biosynthesis

## Abstract

Peptide backbone α-*N*-methylations change the physicochemical properties of amide bonds to provide structural constraints and other favorable characteristics including biological membrane permeability to peptides. Borosin natural product pathways are the only known ribosomally encoded and posttranslationally modified peptides (RiPPs) pathways to incorporate backbone α-*N*-methylations on translated peptides. Here we report the discovery of type IV borosin natural product pathways (termed ‘split borosins’), featuring an iteratively acting α-*N*-methyltransferase and separate precursor peptide substrate from the metal-respiring bacterium *Shewanella oneidensis*. A series of enzyme-precursor complexes reveal multiple conformational states for both α-*N*-methyltransferase and substrate. Along with mutational and kinetic analyses, our results give rare context into potential strategies for iterative maturation of RiPPs.

## Abstract (web summary)

Borosins are ribosomally encoded and posttranslationally modified peptide (RiPP) natural products featuring amide-backbone α-*N*-methylation. Here, the authors report the discovery and characterization of type IV borosin ‘split’ pathways encoding distinct, separate α-*N*-methyltransferases and precursor peptide substrates.

## Introduction

**Fig. 1:**
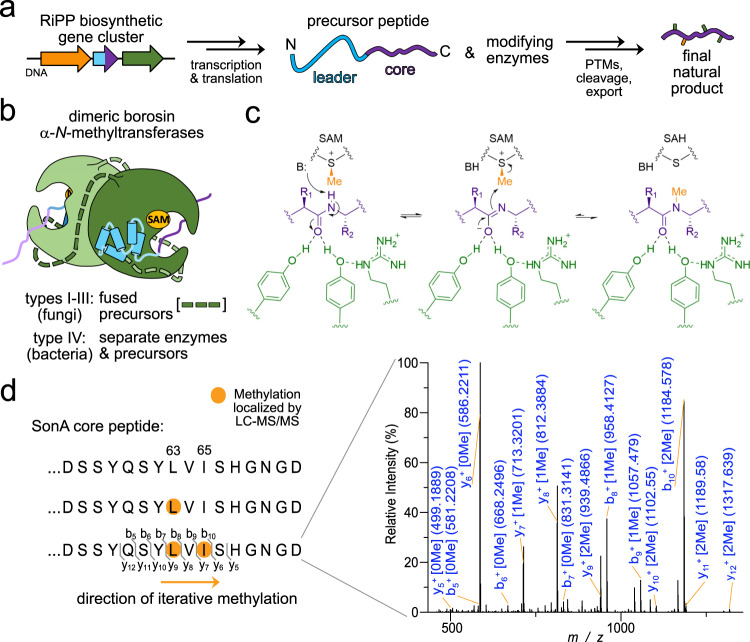
Borosin RiPP biosynthesis. Reaction schemes and protein structure configurations for the biosynthesis of borosin RiPP metabolites. **a** General scheme for RiPP biosynthesis highlighting the typical composition of precursor peptides. **b** Dimeric protein architectures of borosin α-*N*-methyltransferases. Types I–III borosin pathways^24^ encode α-*N*-methyltransferases that are fused (dashed segments) to their precursor peptides, wrapping almost completely around the other subunit to achieve iterative intermolecular α-*N*-methylation. Type IV borosin pathways (reported in this manuscript) have distinct protein architectures, where dimeric α-*N*-methyltransferases associate with two discrete precursor peptides (no connective dashed segments). **c** General reaction mechanism for borosin precursor α-*N*-methylation. Deprotonation of the target amide hydrogen is proposed to be mediated either by an activated water^30^ or conserved tyrosine^31^. **d** SonM-mediated α-*N*-methylation states of SonA. LC-MS/MS data show methylation of SonA-L63 always precedes methylation of SonA-I65, revealing N-to-C directionality for α-*N*-methylation as seen with all borosin methyltransferases to date (see also Supplementary Fig. 4). PTMs post-translational modifications, SAM *S*-adenosylmethionine, SAH *S*-adenosylhomocysteine.

Over the past 25 years, ribosomally synthesized and posttranslationally modified peptides (RiPPs) have proven to be a major class of natural products, whose expanding breadth of unique structures and bioactivities are beginning to rival those of non-ribosomal peptides^1^. Moreover, peptide features such as amide backbone α-*N*-methylations^2,3^ and D-configured residues^4–6^ once thought exclusive to non-ribosomal peptides are now known to also be installed by RiPP biosynthetic pathways. Initially translated by the ribosome, RiPPs are typically synthesized as short precursor peptides that are extensively posttranslationally modified at their C-termini prior to proteolytic maturation and export^1^. The short N-terminal leader peptides of RiPP precursors serve as docking domains that direct an assortment of tailoring enzymes to install post-translational modifications (PTMs) on the C-terminal core peptide destined to become the mature natural product (Fig. 1a)^7,8^. In prokaryotes, RiPPs are often encoded in monocistronic, streamlined biosynthetic gene clusters where encoded tailoring enzymes frequently introduce multiple modifications in a given RiPP precursor^1^. The the combination of modifying enzyme promiscuity and regiospecificity has driven the field to seek structural clarity of these protein complexes and their underlying kinetic constraints to understand the rules governing RiPP PTM incorporation.

In response, numerous structures of RiPP modifying enzymes in complex with their precursors have begun to shed light on the contacts and strategies employed by RiPP biosynthetic enzymes. For many RiPP families, a RiPP leader binding domain of the PqqD superfamily (PF05402) is found either as a discrete protein^9^ or as a domain within tailoring enzymes, now referred to as a RiPP precursor peptide recognition element (RRE)^10^. These winged-helix-turn-helix RRE domains have been observed to interact similarly with leader peptides presented as an antiparallel β-strand, as seen in examples of lasso peptides^11,12^, type I lanthipeptides^13^, and cyanobactins^14^. Still other RiPP heteromeric complexes display short α-helical or random coil portions of precursor peptides that use alternative binding strategies with^15–18^ or without^19–22^ RREs. Nonetheless, the small size and apparent flexibility of precursor peptides have limited their crystallographic resolution to a subset of either leader or core fragments with few or no residues from the other region of the precursor peptide. In addition, the lack of fully resolved intermediates or multiple conformation states in these examples have slowed progress in our understanding of the mechanisms and iterative nature of RiPP biosynthetic machinery^23^.

Recently, a new family of α-*N*-methylated RiPPs named the borosins was discovered as the biosynthetic origins for the nematicidal omphalotins^2,3^ and the antineoplastic gymnopeptides^24^, cyclic peptides produced by the basidiomycete fungi *Omphalotus olearius*^25–27^ and *Gymnopus fusipes*^28^, respectively. Two distinct features define the borosin RiPP family. Their pathways encode the first described α-*N*-methyltransferases that posttranslationally install multiple amide-bond α-*N*-methylations into the backbone of ribosomally derived peptides. Amide-backbone α-*N*-methylations restrict peptide flexibility and impart unique properties to natural products that include biological membrane permeability and favorable pharmacodynamics through proteolytic resistance^29^. The second distinguishing borosin family feature is that these *S*-adenosylmethionine-dependent (SAM-dependent) α-*N*-methyltransferases are encoded in frame as part of unusually long leader peptides, marking the borosins as the first pathways to encode iterative, autocatalytic RiPP precursors (Fig. 1b). Soon after this discovery, the omphalotins precursor Oph(M)A^30^ and a close homolog^31^ were structurally interrogated and found to be the first RiPP precursors to form homodimers, where each α-*N*-methyltransferase acts on the C-terminal core peptide of the other precursor subunit.

Crystal structures of OphMA C-terminal truncations and active site mutants along with biochemical studies and quantum mechanical calculations led to a mechanistic proposal for α-*N*-methylation^30^. Amide-bond polarization and stabilization is hypothesized to trigger water-mediated deprotonation of the target amide nitrogen and consequent imidate anion nucleophilic attack on SAM to yield the α-*N*-methylated amide^30^. The proposed mechanism for the OphMA homolog, dbOphMA, was near-equivalent, with the exception of a conserved tyrosine in lieu of water mediating abstraction of the amide proton (Fig. 1c)^31^. While mass spectrometric analysis of OphMA revealed up to 11 α-*N*-methylations installed in an N-terminal to C-terminal fashion on the core peptide^2^, slow reaction rates^30^ and complications resulting from the core peptide being tethered to the methyltransferase hampered more detailed kinetic characterization. In addition, and perhaps due to its closely associated catenane-like homodimeric complex, only a single conformational state was observed among all of the OphMA structures aside from two subtle core peptide configurations. As a result, few concrete details exist concerning the full catalytic cycle and the driving forces of iterative activity for borosin α-*N*-methyltransferases.

In this manuscript, we structurally and kinetically characterize a model type IV borosin RiPP system harboring distinct protein architectures we informally call “split borosins”. Split borosins are defined by discrete α-*N*-methyltransferases and precursor peptides. In contrast to the original borosins only found in fungi, split borosins appear predominantly in bacteria. Our model system is a conserved putative RiPP cluster encoded in the metabolically dynamic, metal-respiring bacterium *Shewanella oneidensis* MR-1^32^. Crystal structures of the α-*N*-methyltransferase and RiPP precursor heteromeric complex reveal fully resolved leader and core peptides. Moreover, complexes of α-*N*-methyltransferases and/or precursor mutants show substantial conformational changes surrounding the active site as well as gross structural changes with the core peptide based on its methylation state. Along with kinetic characterization and in silico kinetic modeling, we provide a more complete view for α-*N*-methylation in borosin pathways. More generally, our results provide a glimpse into the dynamics and strategies for iteratively acting enzymes in RiPP biosynthetic pathways.

## Results

### Discovery of type IV split borosin pathways

Previous bioinformatics analysis of fungi encoding well-conserved OphMA-like α-*N*-methyltransferase domains uncovered >50 putative borosin pathways that were not associated with known natural products^24^. While we validated >10 additional borosin α-*N*-methyltransferase precursors in the size range of ~400–850 amino acids (AA), all harbored a fused α-*N*-methyltransferase leader and core architecture. Consequently, we set out to mine for homologs that would open possibilities for untethered core peptides to these domains. Upon repeated PSI-BLAST analyses of the fungal borosin α-*N*-methyltransferase domains, we identified a large variety of more distantly related putative homologs in bacteria (Supplementary Fig. 1). These putative bacterial homologs bear all the conserved residues involved in core peptide stabilization and the oxyanion hole in OphMA (Supplementary Fig. 2).

To validate the putative bacterial borosin pathways, we initially focused our efforts on two systems, one found encoded in *Streptomyces* sp. NRRL S-118^32^ and the other in *Shewanella oneidensis* MR-1^33^. Both putative α-*N*-methyltransferases were 263 AA in length (compared to the 417 AA of OphMA) and did not appear to encode a C-terminal clasp domain or core peptide as defined in OphMA^2,30^. Furthermore, short peptides ~75 residues in length encoded adjacent to the putative methyltransferases were hypothesized to serve as discrete precursor peptides (Supplementary Fig. 3). Heterologous expressions of the putative methyltransferase-precursor pairs followed by purification and high-resolution, high-pressure liquid chromatography−mass spectrometric analysis (LC-MS/MS) confirmed them as α-*N*-methyltransferring borosin RiPP pathways. Similar to OphMA and the other fungal borosins, α-*N*-methylations are incorporated in an N-terminal to C-terminal direction on the split borosin precursors (Supplementary Fig. 4). These data verify borosin pathways in bacteria. The distinct architectural composition of modifying enzyme and precursor extends our structural classification of borosin precursors to type IV pathways^24^. We designate type IV borosin pathways informally as “split borosins”, with their defining characteristics as separately encoded α-*N*-methyltransferases and precursor peptides.

### Structures of the SonM—SonA complex

**Fig. 2:**
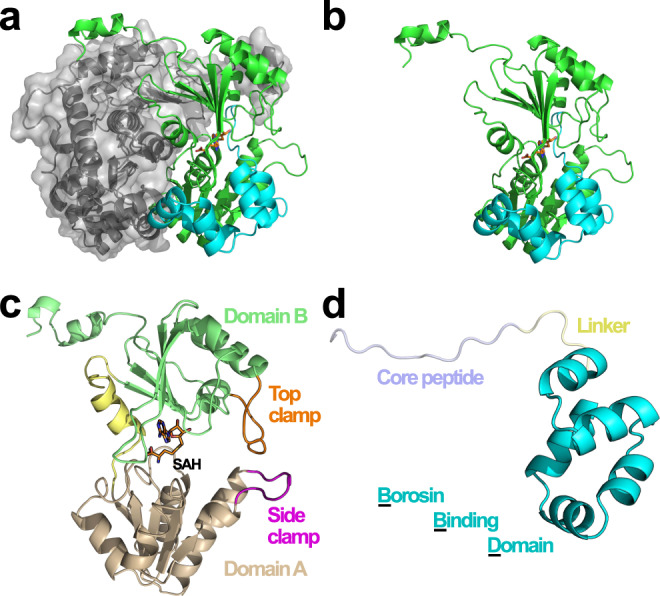
Global architecture of the SonM—SonA complex. The SonM monomer is composed of two α/β/α, Rossmann-like domains with five β-strands each. **a** SonM—SonA-2Me—SAH forms a dimer of heterodimers. One heterodimer is depicted in gray cartoon with a semi-transparent surface. The SonM—SonA-2Me interface involves few polar interactions (two hydrogen bonds, two salt bridges) and is mainly hydrophobic. **b** Each split borosin heterodimer is composed of the α-*N*-methyltransferase SonM (green cartoon) and the precursor peptide SonA (cyan cartoon). **c** The SonM α-*N*-methyltransferase can be further subdivided into two domains, Domain A (SonM 1–120, light brown cartoon) and Domain B (SonM 142–263, light green cartoon) that are linked by a bridging helix (SonM 121–141, yellow cartoon). The spent cofactor SAH (sticks) is buried deep within the active site pocket that is capped with two loops referred to as the top clamp (Domain B; SonM 172–182, orange) and side clamp (Domain A; SonM 58–67, magenta). **d** The precursor peptide is composed of an N-terminal five-helix-bundle termed the borosin binding domain (BBD; cyan cartoon) and a core peptide (slate) connected by a linker (yellow).

The α-*N*-methyltransferase—precursor pair from *S. oneidensis*, SonM (SO1478, UNIPROTKB Q8EGW3) and SonA (SO1479, UNIPROTKB Q8EGW2) was chosen for more rigorous structural and kinetic characterization due to their ease of overexpression, co-purification, and modest two α-*N*-methylations installed on the SonA backbone. In addition, the small *S. oneidensis* split borosin gene cluster is conserved in the majority of sequenced *Shewanella* genomes (Supplementary Table 1). While we were unable to obtain crystals of SonM or SonA individually, we were successful in determining a series of structures of SonM in complex with SonA, either bound or unbound with the methyl-donating cofactor *S*-adenosylmethionine (SAM) or its product *S*-adenosylhomocysteine (SAH). Complexes of SonM and SonA form dimers of heterodimers (Fig. 2a, b). Two molecules of SonM arrange in back-to-back orientation, each predominantly associated with a single SonA monomer with its core peptide bound and fully resolved in the SonM active site. This configuration differs from homologous systems such as homodimeric OphMA and dbOphMA, where a clasp region and core peptide is directly fused to the methyltransferase (Supplementary Fig. 5). Of note, individual expression and purification of SonM and SonA reveal their native oligomeric states as a dimer and monomer, respectively (Supplementary Fig. 6). Therefore, SonM, like all borosin methyltransferases characterized to date, is active as a homodimer.

A five helical bundle at the N-terminus of SonA, now referred to as the borosin binding domain (BBD; 1–53), serves as the SonA leader peptide and is homologous to a portion of the fused clasp region of OphMA. While major structural differences exist between OphMA and SonM—SonA complexes from translations of the BBD of up to 10 Å (Supplementary Fig. 7), the wildtype (wt) SonM—SonA heterodimer globally organizes in a similar orientation to OphMA (1.1 Å over 220 α-carbon atoms) and dbOphMA (0.9 Å over 209 α-carbon atoms). Interestingly, structural homologs of the BBD are encoded in other metabolic pathways. Homologous structures, used as either *trans* or *cis* domains as in fungal and bacterial borosin pathways, are found capping the active sites of dioxygenases (Supplementary Fig. 8).

### Fully resolved SonM—SonA-2Me and SonM—SonA-2Me—SAH complexes

**Fig. 3:**
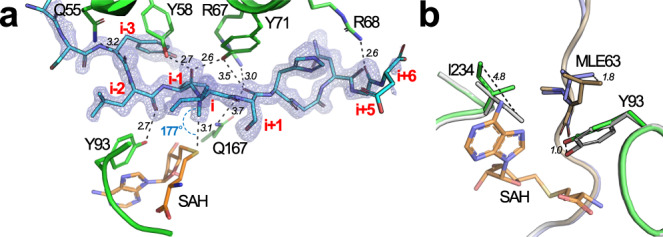
The SonA core peptide is fully resolved and doubly α-*N*-methylated. **a** Fourier difference electron density map (*F*_obs_ − *F*_calc_; blue mesh) contoured at 3σ level for the SonA core peptide (cyan sticks) in the SonM—SonA-2Me—SAH complex unambiguously reveal a fully resolved sequence with two residues, SonA-L63 (“i−2”) and SonA-I65 (“i”), α-*N*-methylated. The position “i” refers to SonA residues in SonM—SonA-2Me complexes that are in line to attack the cofactor SAM; negative “i” values count towards the SonA N-terminus, while positive “i” values count up towards the C-terminus. **b** Comparison between the *apo* (SonM, white; SonA-2Me, light brown) and SAH-bound (SonM, green; SonA-2Me, slate; SAH, orange sticks) structures of SonM—SonA-2Me complexes. Key distances are depicted as black dashed lines and their lengths noted in italics in Ångstroms.

Structural features identified with substrate binding are shown in Fig. 2c. Notably, two loops, termed the top clamp and side clamp, appear to enclose the active site. The complete peptide chain of SonA-2Me could be modeled, including the BBD leader, the linker region (SonA 54–61), and the putative core peptide (62–71) (Fig. 2d). SonM and doubly methylated SonA (SonA-2Me) interact tightly via the BBD of SonA-2Me (652.8 Å^2^). The BBD interacts with both domains of SonM, yet the major interaction consists of the wrapping of the BBD around a protruding loop from Domain B (SonM 208–216). The linker region shows some conformational heterogeneity for side chain conformations and consequently exhibits the highest thermal motion B-factor values of the SonM—SonA-2Me structure (Supplementary Fig. 9). Inspection of electronic density maps reveal a fully resolved core peptide that sits in the active site channel of SonM (Fig. 3). Electron density maps unambiguously reveal that the SonA peptide is doubly methylated at residues 63 and 65 as *N*-methyl-l-leucine (MLE) and *N*-methyl-l-isoleucine (IML), respectively; the α-*N*-methyl group of SonA-IML65 is positioned 3.1 Å away from the donor sulfur atom of the cofactor SAH. The angle (N(amide)-C(methyl)-S(SAH)) is 177° (Fig. 3a), close to the ideal angle value (180°) and consistent with an S_N_2 mechanism for methyl transfer. Using a similar nomenclature to previous work on OphMA, we define the closest amide nitrogen to the methyl group of SAM (SonA-IML65) as “i” hereafter. The core peptide of SonA-2Me is hydrogen bonded to seven SonM residues via backbone interactions (Fig. 3a). Both SonM-Y71 and SonM-Y58 are both in hydrogen bonding distance with three main chain groups of SonA-2Me (Supplementary Fig. 10). The side chain of SonM-Y71 interacts with the carbonyl and the NH groups of “i+1” (3.5 Å and 3.2 Å, respectively), and the carbonyl group of “i−1” (2.6 Å). The side chain of SonM-Y58 interacts with the carbonyl and the NH groups of “i−1” (2.7 Å and 3.6 Å, respectively) and the carbonyl group of “i−3” (3.6 Å). Overall, with the exception of the relatively large, hydrophobic pocket at position “i”, SonM maintains mostly SonA backbone interactions that are agnostic to side chain composition (Supplementary Fig. 11).

Unlike OphMA and its close homolog dbOphMA, the structure of SonM—SonA-2Me could be solved with (PDB: 7LTE [10.2210/pdb7LTE/pdb]) and without (PDB: 7LTC [10.2210/pdb7LTC/pdb]) SAH/SAM; the structures superimpose with minimal differences (Supplementary Fig. 12). While SAH is bound to the protein by an extensive network of interactions (Supplementary Fig. 13), the *apo* structure reveals the presence of water molecules filling the cofactor’s cavity. Most differences between the structures relate to concerted rotamer changes due to steric hindrance upon SAH binding. Specifically, upon SAH binding, the side chain of SonM-Y93 relocates by 1 Å and the side chain of SonM-I234 rotates nearly 180° to increase the cofactor binding pocket volume (Fig. 3b). Interestingly, changes in SonM-Y93 conformation induces a conformation change (up to 1.8 Å) of the side chain of i−2 SonA-MLE63. These changes appear necessary to sterically allow for cofactor exchange.

### Kinetic parameters and key residues

Sequence analysis shows several residues are conserved in closely related borosin methyltransferase domains (Supplementary Fig. 2). In combination with structural analysis, we decided to evaluate SonM active site mutants SonM-Y93F, SonM-R67K, SonM-R67A, SonM-Y58F, SonM-Y71F, and the double mutant SonM-Y58F/SonM-Y71F. These mutants were assessed for their involvement in catalysis as well as for their role in the binding of cofactor and substrate. A continuous, coupled-kinetics assay was employed to infer α-*N*-methylation events through the conversion of NADPH to NADP^+^ and the concomitant decrease in A_340_^34^. This assay eliminates the buildup of SAH, which inhibits some methyltransferases. Apparent steady-state Michaelis–Menten parameters were determined for both SAM and SonA (Table 1 and Supplementary Fig. 14a). It should be noted that the complexity of these reactions are beyond the constraints of Michaelis–Menten kinetics assumptions, since one cofactor and two possible substrates—SonA and singly methylated SonA-1Me—are present during the course of the reactions^35^. Nonetheless, the data and curves fit well to the models, and thus we posit the reported kinetic values are valid approximations.

**Table 1:** Apparent steady-state kinetic parameters.

SonM	*K*_M_ (μM)	Fold Δ to wt	*k*_cat_ (min^−1^)	Fold Δ to wt	*k*_cat_/*K*_M_ (M^−1^ s^−1^)	Fold Δ to wt
*Apparent steady-state parameters: saturating SAM with varying concentrations of SonA*						
wt	8.2 ± 1.5	–	0.52 ± 0.023	–	1.1 × 10^3^ ± 0.20 × 10^3^	–
Y93F	6.2 ± 1.0	1.3 ↓	0.25 ± 0.0087	2.1 ↓	0.67 × 10^3^ ± 0.11 × 10^3^	1.6 ↓
Y58F	7.6 ± 1.0	1.1 ↓	0.034 ± 0.0016	15 ↓	0.074 × 10^3^ ± 0.011 × 10^3^	14 ↓
R67K	18 ± 4.1	2.3 ↑	0.012 ± 0.00077	43 ↓	0.011 × 10^3^ ± 0.0025 × 10^3^	97 ↓
Y71F	9.6 ± 1.2	1.2 ↑	0.0061 ± 0.00018	84 ↓	0.011 × 10^3^ ± 0.0013 × 10^3^	98 ↓
Y58F, Y71F	*n.d*.	–	*n.d*.	–	*n.d*.	–

*Apparent steady-state parameters: saturating SonA with varying concentrations of SAM*						
wt	56 ± 8.5	–	0.47 ± 0.014	–	1.4 × 10^3^ ± 0.021 × 10^3^	–
Y93F	220 ± 39	3.8 ↑	0.24 ± 0.011	2.0 ↓	0.018 × 10^3^ ± 0.0034 × 10^3^	1.6 ↓
Y58F	47 ± 8.7	1.2 ↓	0.030 ± 0.0011	16 ↓	0.011 × 10^3^ ± 0.0020 × 10^3^	14 ↓
R67K	36 ± 10	1.6 ↓	0.011 ± 0.00054	43 ↓	0.0051 × 10^3^ ± 0.0014 × 10^3^	97 ↓
Y71F	82 ± 1.5	1.5 ↑	0.0066 ± 0.00033	71 ↓	0.0014 × 10^3^ ± 0.00030 × 10^3^	98 ↓
Y58F,Y71F	*n.d*.	–	*n.d*.	–	*n.d*.	–

*Inhibition data: saturating SAM with varying concentrations of SonA and SonA-BBD*						
**SonM**	***K***_**i**_**(μM)**	–	–	–	–	–
wt	3.9 ± 0.5	–	–	–	–	–

*Inhibition data: saturating SAM with varying concentrations of SonA and SonA-2Me*						
**SonM**	***K***_**i**_**(μM)**	–	–	–	–	–
wt	160 ± 26	–	–	–	–	–

SonM wt and mutant kinetics data for varying concentrations of SonA and SAM separately. The value of each kinetic parameter is shown with its standard deviation. Downward arrows indicate fold decreases compared to wt, whereas upward arrows indicate fold increases. Inhibition data (wt) is given separately for varying concentrations of SonA-BBD and SonA-2Me. See Supplementary Figs. 14 and 17 for further details.

We performed kinetic measurements by varying SAM concentration while keeping SonA concentration constant, and in a second set of measurements we varied SonA concentration while keeping the cofactor concentration constant. Both measurements indicate a relatively fast enzymatic system compared to OphMA, with an apparent *k*_cat_ of 0.52 min^−1^ and 0.47 min^−1^ when varying SonA and SAM concentrations, respectively. Interestingly, the apparent *K*_M_ for SonA is lower than the apparent *K*_M_ for SAM (8.2 μM vs. 56 μM, respectively). We note that the *K*_M_ for SAM is similar in magnitude to the measured SAM *K*_d_ for dbOphMA^31^.

We mutated all candidate residues that could play a role in catalysis. All mutants showed reduced catalytic parameters, and all except the double mutant SonM-Y58F/SonM-Y71F have measurable activity in the coupled kinetics assay (Supplementary Fig. 14b). These results may rule out a direct catalytic role for the mutated residues, such as the proposed role of the residue corresponding to SonM-Y71 in dbOphMA^31^. The deprotonation of the backbone NH group and the potential channeling of the proton therefore remain elusive. Yet, mutational work reveals key residues for active site electronic and spatial pre-organization, as well as cofactor binding. Among the mutations targeting residues directly interacting with the core peptide, SonM-Y93F shows the smallest changes in catalytic efficiency, as compared to the wt enzyme. However, the mutant exhibits the largest change in the apparent *K*_M_ value for SAM (220 μM, a 3.8-fold increase as compared to wt). We obtained the structure of the SonM-Y93F mutant bound to SonA-2Me in absence of cofactor (PDB: 7LTH [10.2210/pdb7LTH/pdb]). While, globally, the SonM-Y93F mutant structure complex does not reveal significant differences to wt (Supplementary Fig. 15a), the structural observation that mutation of SonM-Y93 incurs a conformational change upon SAH binding (Fig. 3b) is consistent with the increased apparent *K*_M_ value and a role in binding SAM.

The two other active site tyrosine residues, SonM-Y58 and SonM-Y71, were also mutated to phenylalanine. Both residues mainly affect the apparent *k*_cat_, where SonM-Y71F is more deleterious than SonM-Y58F (~100-fold and ~10-fold decrease in catalytic efficiency, respectively). While we could not crystallize the SonM-Y71F—SonA-2Me complex, the SonM-Y58F—SonA-2Me structure (PDB: 7LTF [10.2210/pdb7LTF/pdb]) was solved (Supplementary Fig. 15b). The overall structure is very similar to wt. Loss of the hydrogen bond between the carbonyl group of “i−1” and SonM-Y58 results in minor changes, including a slight change in the interaction angle (from 108° to 114°) and a deviation from amide bond planarity (*Ω* angle is 178° and 174° in wt and SonM-Y58F, respectively).

### Kinetics modeling suggests methylations are near-equivalent

The methylation of two different substrates (SonA-0Me and SonA-1Me) contribute to the overall kinetic parameters measured in our experiments. SonA-1Me is not yet available to test as a discrete substrate. In an effort to disentangle the relative contributions of each substrate in these iterative reactions, we compared in vitro substrate and product ratios with in silico kinetic models using a simplified reaction coordinate (see “Methods” section). Duplicate time points of SAM-saturated SonM and SonA reactions were run for 2, 8, 20, 40, and 60 min. Ratios of SonA-0Me, SonA-1Me, and SonA-2Me were estimated through relative LC-MS peak integrations of core peptide fragments (Supplementary Fig. 16). Near-exact retention times and similar overall peptide physiochemical properties afford reasonable estimations of relative peptide abundance during the course of the reactions. The buildup of the intermediate SonA-1Me in these reactions (up to ~25% total SonA, ~25 μM) is well beyond the concentration of SonM (5 μM) and unequivocally shows SonM is not exclusively a processive enzyme.

**Fig. 4:**
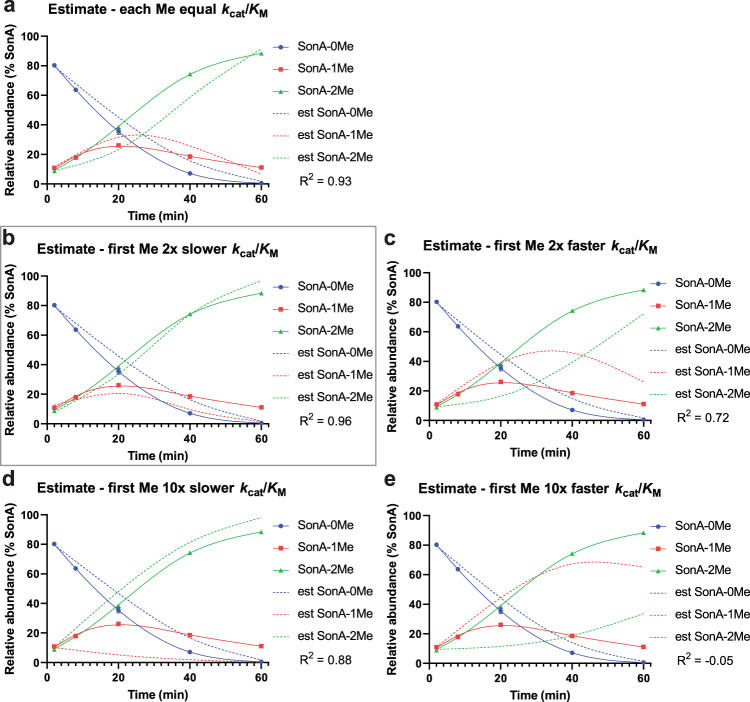
Kinetics modeling of the iteratively acting α-*N*-methyltransferase SonM. Graphical comparisons of in silico (dashed lines) and in vitro (solid lines) SonM reactions with the relative levels of substrate (SonA-0Me, blue) and the products of the first (SonA-1Me, red) and second (SonA-2Me, green) methylations plotted. Each graph contains the output of a simplified kinetics model (see “Methods” section) showing the estimated relative abundance of each species of SonA as dashed lines. Each panel contains a simulation for a different combination of relative *k*_cat_/*K*_M_ values for the first and second methylation (Me) events. R^2^ represents the fit of the model to data from two replicates. Data points are fit against the model prediction for the corresponding species. **a** Equal simulated *k*_cat_/*K*_M_ values; **b** the first Me is simulated with a 2× slower *k*_cat_/*K*_M_ value; **c** the first Me is simulated with a 2× faster *k*_cat_/*K*_M_ value; **d** the first Me is simulated with a 10× slower *k*_cat_/*K*_M_ value; **e** the first Me is simulated with a 10× faster *k*_cat_/*K*_M_ value. Relative SonA abundances from duplicate, timed in vitro reactions and observed on LC-MS/MS (solid lines) are overlaid in each plot (Supplementary Fig. 16). The in silico predictions and in vitro data match best in **b** (boxed in gray), where the second methylation of SonA is predicted to have a modestly higher relative catalytic efficiency than the first methylation of SonA.

In an effort to determine SonM substrate preferences, a series of in silico SonM and SonA reaction simulations were conducted in parallel, where we varied the relative *k*_cat_/*K*_M_ values for substrates SonA-0Me and SonA-1Me to either ten-fold higher/lower, two-fold higher/lower, or equal *k*_cat_/*K*_M_ values. The predicted concentrations of substrates and products in these simulations were then overlaid with the observed in vitro data. As seen in Fig. 4, the in silico data best fits with a modest two-fold more efficient second methylation event as compared to the first. These analyses are unable to discern contributions of local substrate concentration or other factors such as individual impacts to *K*_M_ and *k*_cat_ for each substrate. Nonetheless, these data support the hypothesis that substrate preference is not strongly skewed in favor of either SonA-0Me or SonA-1Me during the course of the reactions.

### SonM—SonA-BBD—(±)SAM reveals distinct conformations

All SonM active site mutants affect the apparent *k*_cat_ and not the apparent *K*_M_ for SonA. This observation is in line with previous reports claiming leader peptides are the source of molecular recognition and binding affinity for many RiPP biosynthetic enzymes^7,8^. To further test the role of the core peptide on the binding of SonA to SonM, the *K*_i_ for a BBD-only construct (SonA-BBD; 1–55) was estimated to be 3.9 μM (Table 1 and Supplementary Fig. 17a). While not directly comparable to the SonA apparent *K*_M_ of 8.2 μM, it suggests that the BBD is the major SonA-binding contribution. Additionally, we determined the apparent *K*_i_ for the SonA-2Me product to be 160 μM (Table 1 and Supplementary Fig. 17b). Combined with our previous finding that SonM shows little substrate preference for either SonA-0Me or SonA-1Me, the SonA-2Me *K*_i_ suggests the presence of the second methylation significantly reduces the binding affinity to the active site and thereby favors product release.

**Fig. 5:**
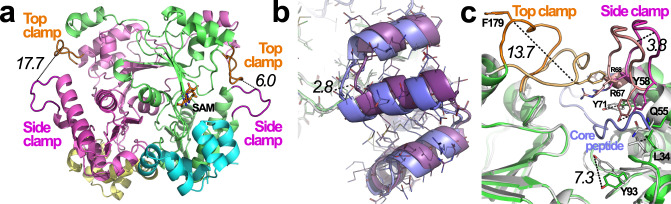
SonM—SonA-BBD—(±)SAM structure showcases closed and open conformations. **a** One SonM—SonA-BBD heterodimer (green and cyan cartoon, right) has SAM bound (orange sticks) in the active site with the top and side clamps in a previously seen closed conformation. The other SonM—SonA-BBD heterodimer (magenta and yellow cartoon, left) lacks a bound cofactor and display an open conformation for both top and side clamps. **b** Structural overlays of the BBDs from the *apo* SonM—SonA-2Me complex (slate cartoon) and the heterodimer in the open conformation for the SonM—SonA-BBD—(±)SAM complex (purple cartoon). The BBDs bind similarly but appear to be translated from each other. **c** Structural overlays of the active sites from the *apo* SonM—SonA-2Me complex (white and slate cartoon) and the heterodimer in the open conformation for the SonM—SonA-BBD—(±)SAM complex (green cartoon). Loop differences and residue shifts (sticks) are highlighted. Key distances are depicted as black dashed lines and their lengths noted in italics in Ångstroms. We note that crystal contacts may contribute to the configuration of the open form of the top clamp. However, the observation of both open and closed forms of the top clamp within the same crystal strongly suggests the two conformations precede crystal packing.

The structure of the complex SonM—SonA-BBD—(±)SAM (PDB: 7LTR [10.2210/pdb7LTR/pdb]) was solved. Notably, the structure captures two completely distinct conformations, differing by (I) the presence or the absence of the cofactor SAM and (II) a very large conformational change, an opening of the top and side clamp active site loops (Fig. 5a).

### Closed and opened SonM conformations

Indeed, one of the heterodimers is bound to the cofactor SAM, and the overall conformation is similar to the heterodimer SonM—SonA-2Me—SAH structure (Supplementary Fig. 18). The comparison of these structures suggests that the BBD in SonA-BBD binds similarly as full-length SonA-2Me, albeit slightly translated (~1.3 Å). This change could be due to the absence of the core peptide, or to conformational changes induced by the binding of SAM in lieu of SAH.

The cognate SonM—SonA-BBD heterodimer is without cofactor, and shows large conformational changes as compared to the *apo* SonM—SonA-2Me structure. Again, the BBD binds similarly as in SonA-2Me, yet is translated farther, ~2.8 Å (Fig. 5b). In the absence of cofactor, it is likely that this change is due to the absence of the core peptide. The active site binding cleft in the SonM—SonA-BBD heterodimer appears largely open, and there are diverse conformational changes compared to the other, cofactor-bound heterodimer. In fact, a large portion of Domain A of SonM appears translated by ~3 Å as a rigid body. The active site residues SonM-Y71 and SonM-Y58 are translated by ~2.8 Å, and SonM-F99, a residue involved in cofactor binding that interacts with Domain B, shifts ~2.4 Å. Numerous residues adopt different conformations, such as SonM-L34, SonM-Q55, SonM-F179, SonM-R68, and SonM-R67. Most prominently, the residue SonM-Y93 that interacts with the cofactor adopts a completely different rotamer (the hydroxyl group of the side chain differs by 7.3 Å between the *apo* heterodimer SonM—SonA-BBD and the *apo* SonM—SonA-2Me structure; Fig. 5c). Consequently, the cofactor cavity is significantly larger, and the ability of SonM-Y93 to switch between an inward trajectory towards the cofactor and outwards towards solvent and suggests a role in cofactor binding and release. This structural data corroborates the increase in the apparent *K*_M_ of SAM for the SonM-Y93F mutant (Table 1).

The SonM active site loops show large conformational changes as compared to other SonM—SonA-2Me structures (Fig. 5c). The side clamp is translated by ~3.5 Å and contributes to the opening of the active site cavity. The top clamp undergoes the largest conformational change. The top clamp that is typically observed in closed conformation in the other SonM—SonA-2Me structures, but also in OphMA and dbOphMA structures, relocates by nearly 14 Å. These very large conformational changes and active site opening (Supplementary Movie 1) may be critical for cofactor binding, core peptide binding, re-positioning after first turnover, and product release.

### Structural determinants for transition between conformations

**Fig. 6:**
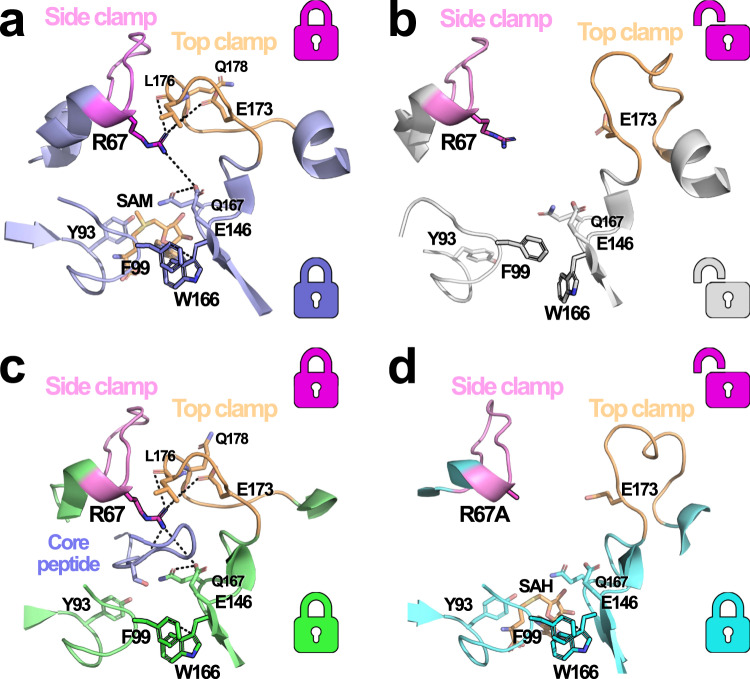
Interaction networks enabling multiple conformational states. **a** The closed conformation in the SonM—SonA-BBD—(±)SAM complex with SAM (orange sticks) bound in the active site. The hydrogen-bonding network involving SonM-R67 closes the top lock in the active site, while the π-stacking of SonM-F99 and SonM-W166 forms the bottom lock. **b** The open conformation in the SonM—SonA-BBD—(±)SAM complex without SAM reveals open top and bottom locks. **c** The *apo* SonM—SonA-2Me complex reveals both the top and bottom locks closed, using a similar network of interactions seen in **a** but with the core peptide present and cofactor absent. **d** The SonM-R67A—SonA-0Me—SAH complex is unable to form the top lock due to mutation of SonM-R67 but maintains the bottom lock contacts through binding of SAH. The core peptide in the active site of this complex was removed in this image for clarity. Key hydrogen bonds and distances are depicted as black dashed lines. Top and bottom locks are illustrated with corresponding color-coded icons next to each motif.

The comparison of the two heterodimers of SonM—SonA-BBD—(±)SAM provides insight on the structural determinants that allow for the change between the open and closed conformation. Indeed, a careful analysis and comparison of both conformations reveals an interaction network between the cofactor SAM, the side clamp, and the top clamp (Fig. 6a, b). Specifically, the binding of SAM, and in particular the ribose moiety, appears to sterically hinder and induce a conformational change for residues SonM-W166 and SonM-F99. Relocation of SonM-F99 (Domain A; ~2.8 Å) and SonM-W166 (Domain B; ~2 Å) brings both residues closer (4.8 Å in the *apo* structure vs. 3.4 Å) to a near-canonical π–π sandwich-stacking configuration (Supplementary Fig. 19). We denote this key interaction as the “bottom lock”. As a result, the two SonM domains are drawn closer to each other. Movement of SonM-W166 also relocates its adjacent residue SonM-Q167 and its hydrogen bonding partner SonM-E146. SonM-E146 relocation, combined with the closing of both domains, allows for SonM-Q167 to perform an ionic interaction with SonM-R67 (4.8 Å). This interaction positions the side chain of SonM-R67 close enough to Domain B’s top clamp to form a second salt bridge interaction with SonM-E173 (5.0 Å). SonM-R67 may also interact with the main chain carbonyl groups of SonM-Q178 (4.3 Å) and SonM-L176 (4.0 Å). The latter carbonyl performs the only other specific interaction between the two clamps, a hydrogen bond with the SonM-R67 backbone NH (2.9 Å). We denote this key interaction network as the “top lock” (Supplementary Fig. 20a).

We therefore find that SonM can adopt a closed conformation in the absence of the core peptide and with cofactor (SAM) bound, and also in the absence of cofactor with the core peptide bound. Structural analysis of the different structures reveals that the cofactor and the core peptide connects the same network of interactions and, therefore, both bottom and top locks (Fig. 6a, c). In fact, a large fraction of the involved residues interacts either nonspecifically (SonM-E146) or specifically (SonM-Q178; SonM-R67) with the core peptide. Similarly to the binding of the cofactor, the core peptide sterically clashes with SonM-F99 and may promote the formation of the observed π–π sandwich stacking. The establishment of this closed conformation interaction network is further promoted by the binding of the core peptide with the hydrogen bonding between SonM-R67 with the carbonyl group of “i+1” (Fig. 6c).

In sum, results from in vitro data, kinetic modeling, and the alternative active site conformation observed in the SonM—SonA-BBD—(±)SAM structure strongly suggest SonA can partially or completely dissociate between methylation events and that SonM is not a strictly processive enzyme. Structural analysis also suggests that binding of the core peptide or the cofactor can trigger SonM’s closed conformation via the same network of interactions.

### SonM-R67 is critical for clamp and SonA core conformations

Careful structural analysis of the SonM—SonA-2Me, SonM—SonA-2Me—SAH, and SonM—BBD-SonA—(±)SAM structures reveal that SonM-R67 may play multiple roles: (I) a role in spatial and electronic active site pre-organization via its interaction with SonM-Y71, (II) a role in direct substrate binding via its interaction with the carbonyl group of residue “*i* + 1”, and (III) a third role stabilizing the top clamp’s conformation via a complex network of interactions described above. We note that these roles seem to be conserved, since equivalent residues to SonM-R67 in OphMA appear to be involved in similar interactions (Supplementary Fig. 20b).

In response, we mutated residue SonM-R67 to both lysine and alanine. SonM-R67K, a mutation that could compensate for some but not all of the roles described above, resulted in a 100-fold decrease in SonM catalytic efficiency. The mutation SonM-R67A, likely disrupting all side chain interactions, had no discernable methyltransferase activity beyond trace amounts detected by LC-MS/MS. We have so far been unsuccessful in crystallizing the SonM-R67K—SonA complex. In contrast, we were able to solve the structure of SonM-R67A—SonA-0Me—SAH (PDB: 7LTS [10.2210/pdb7LTS/pdb]). As expected, this mutation disrupts the previously mentioned interaction network (top lock). Consequently, the structure of the complex reveals an open conformation (Fig. 6d). Despite the overall conservation of the interaction network in the methyltransferase, the equivalent SonM-R67A mutant in OphMA did not yield an open conformation, possibly due to a much longer top clamp loop that makes additional, extensive contacts with both the side clamp and the core peptide (Supplementary Fig. 20).

However, the structure of SonM-R67A—SonA-0Me—SAH is distinct from the open structure observed in the SonM—SonA-BBD heterodimer. In fact, the structure captures an intermediate conformation between the observed closed conformations (e.g., *apo* or SAH-bound wt structures) and the open conformation of the SonM—SonA-BBD heterodimer (Supplementary Fig. 21). In the above-described closed structures, both bottom and top locks were closed, while in the open structure both locks were open. In this structure, the bottom lock (the π–π sandwich stacking between SonM-W166 and SonM-F99) is closed, concomitant to the binding of SAH, whereas the top lock is open due to the SonM-R67A mutation (Fig. 6d).

Similar to other structures of the SonM—SonA-2Me complex, the closed bottom lock brings both SonM domains closer to one another. Consequently, and contrary to the open conformation of the SonM—SonA-BBD heterodimer, the bottom lock and neighboring residues from Domain A such as SonM-Y93 adopt conformations that are superimposable to the wt closed structure. However, the side clamp adopts a conformation that is intermediate between the closed and the fully opened conformations of the SonM—SonA-BBD heterodimer (Supplementary Fig. 21). Due to the SonM-R67A mutation, the top lock is disrupted (Supplementary Fig. 22). We note that the two heterodimers are not strictly identical, and while they globally adopt the same conformations including loops, the two clamps as well as the linker domain of SonA show some conformational variability (Supplementary Fig. 23).

### SonM-R67A—SonA-0Me—SAH reveals metamorphic properties of SonA

**Fig. 7:**
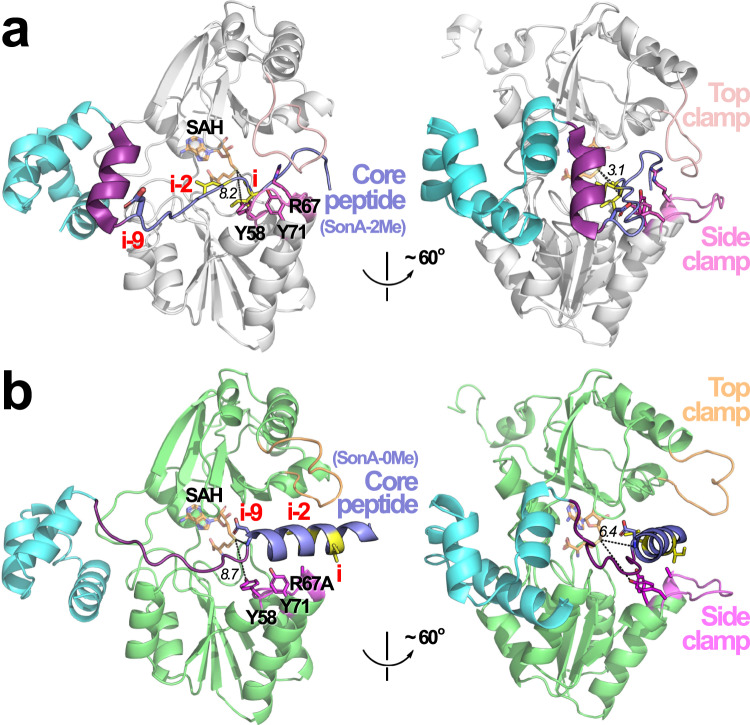
Global differences in structures of SonM—SonA-2Me—SAH and SonM-R67A—SonA-0Me—SAH complexes. **a** One SonM—SonA-2Me—SAH heterodimer is shown, where SonM is represented in white cartoon, the BBD of SonA-2Me is shown in cyan cartoon, and the core peptide is shown in slate. α-*N*-Methylated residues “i” and “i–2” are depicted as yellow sticks. Helix 5 of the BBD is highlighted purple. The two structures in this panel are rotated ~60 degrees to view the structure from two different perspectives. **b** One SonM-R67A—SonA-0Me—SAH heterodimer is shown, where SonM is represented in light green cartoon, and all other portions of the structure are colored and depicted as in **a**. Note that residues “i” and “i−2” are not methylated in this structure as SonM-R67A is an inactive mutant. Key distances are depicted as black dashed lines and their lengths noted in italics in Ångstroms. In comparison to **a**, the SonM-R67A—SonA-0Me—SAH complex reveals a pre-reacted state, where the top lock and clamps are open and the core peptide is more than 3 Å farther away from the cofactor. More significantly, the core peptide is α-helical and positions deeper into the active site, resulting in the BBD helix 5 being unwound into a coil.

The most notable feature of the SonM-R67A mutant structure relates to the significant conformational and secondary structural change of SonA, including the core peptide itself. The BBD is bound to SonM-R67A in a similar conformation to the one observed in the SonM—SonA-BBD heterodimer, i.e., translated ~4.8 Å compared to the closed structure SonM—SonA-2Me—SAH (Supplementary Fig. 24). Interestingly, the core peptide is bound in the active site of SonM as an α-helix (3.5 turns). Concomitantly, helix 5 of the BBD (2.5 turns; as observed in the wt structure) is entirely unwound in the SonM-R67A—SonA-0Me—SAH complex. Comparison between the wt SonM—SonA-2Me—SAH and the SonM-R67A—SonA-0Me—SAH complexes highlights the extent of the change and reveals that unwinding of the BBD’s helix 5 appears necessary to allow the longer section of the core peptide (SonA 53–71 of SonM-R67A—SonA-0Me—SAH vs. SonA 62–71 of wt) to enter the active site in the form of an α-helix (Fig. 7). In contrast to all previously described structures, the SonM-R67A—SonA-0Me—SAH structure is not a post-reaction complex. The helical core peptide is not methylated as expected, since this mutant was not active in our kinetics assay and α-*N*-methylation disrupts backbone hydrogen bonding in α-helices^36^. The binding of the core peptide “deeper” into the active site of SonM is consistent with the expected iterative activity of the enzyme. The observation that helix 5 of the BBD can fold and unfold may contribute to iteratively incorporated methylations (Supplementary Movie 2).

The helical core peptide harbors few specific contacts in the SonM-R67A—SonA-0Me—SAH complex (Fig. 7b). We note that the structure is a pre-reaction complex but is not in a productive conformation: the closest core peptide backbone NH group to the sulfur atom of SAH is “i−9” (6.4 Å). Residue “i” is therefore approximately two α-helix turns away from the first methylated residue in the native complex. SonM-R67A is also not in a catalytically active conformation. The active site is in an open conformation, where the distance between the SAH sulfur atom and the hydroxyl group of SonM-Y71, that are located on opposite sides of the attacked peptide bond, is 8.7 Å vs. 8.1 Å in the closed wt complex. We note that in the absence of the conserved interaction of SonM-R67 with SonM-Y71, and despite significant Domain A movement, the distance between the hydroxyl groups of SonM-Y71 and SonM-Y58 is still conserved (4.9 vs. 4.8 Å in wt). Therefore, SonM-R67 has little importance in the pre-alignment of SonM-Y71 and SonM-Y58. However, the SonM-R67A—SonA-0Me—SAH structure suggests SonM-R67 is critical for the top clamp conformation, core peptide configuration, and the positioning of the complex into productive modes.

## Discussion

RiPP biosynthetic pathways have garnered increasing interest in the last several decades due to their streamlined genetic architecture encoding iteratively acting enzymes that produce diverse natural products. Our results detail the structural and kinetic characterization of a model α-*N*-methyltransferase—precursor pair from a distinct structural type (type IV) of split borosin RiPP biosynthetic pathways. The distinguishing features of split borosin pathways are their discretely expressed precursors and their predominance in bacteria, as compared to the unusual fusion of core peptides to α-*N*-methyltransferase domains in fungal borosin pathways. The discovery of type IV split borosin pathways opens up new avenues of research as none of the pathways are yet associated with a final natural product.

This work reports the first detailed kinetic analyses of borosin α-*N*-methyltransferases, since previously discovered fungal borosin pathways were hampered by tethered substrate peptides and slow reaction rates. The α-*N*-methyltransferase SonM from *Shewanella oneidensis* MR-1 iteratively methylates its substrate SonA at two residues, SonA-L63 and SonA-I65, in an N-to-C terminal fashion on the core peptide. The catalytic efficiency of SonM was determined to be 1.1 × 10^3^ M^−1^ s^−1^ when varying the precursor peptide SonA with saturating levels of the cofactor SAM. This value, along with our other kinetics data, places SonM among other characterized SAM-dependent *N*-methyltransferases (reported bounds of upper 10^4^ M^−1^ s^−1^)^37,38^ and within range of the calculated average of ~7 × 10^4^ M^−1^ s^−1^ *k*_cat_/*K*_M_ for secondary metabolic enzymes^39^. The apparent SonM *k*_cat_ of 0.52 min^−1^ is considerably faster than the observed 0.002 min^−1^ apparent *k*_cat_ for OphMA in vitro^30^. Kinetics analysis of SonM active site mutants support a previously proposed mechanism for borosin methyltransferases, where deprotonation of the target amide nitrogen is stabilized by residues SonM-Y58, SonM-Y71, and SonM-R67. Measurable yet reduced activity of the active site mutant SonM-Y71F disfavors a previously proposed hypothesis for the synonymous residue in dbOphMA in deprotonating the amide nitrogen^31^.

The SonA leader peptide encodes a five-helix bundle we have termed the borosin binding domain (BBD). This BBD leader motif has not been observed in other RiPP classes. In common with other RiPP systems, however, the BBD leader peptide provides the predominant binding contributions of SonA to SonM, as evidenced by our SonM-mutant kinetics and SonA-BBD *K*_i_ data. Mass spectrometric analyses of in vitro reactions compared to in silico reactions further support a non-processive model for SonM activity and revealed the relative efficiency for each methylation event to be near-equivalent (Fig. 4 and Supplementary Fig. 16).

The suite of crystalized SonM—SonA complexes described in this manuscript display fully resolved and contiguous peptide backbones for a RiPP enzyme in complex with its precursor. More importantly, these structural complexes reveal dramatically different structural states depending on the presence or absence of bound cofactor and/or core peptide. Top and side clamps enclose the SonM active site and are linked through a concerted network of residues that trigger both a bottom lock and/or top lock in the active site (Fig. 4). The described locks are closed in the presence of bound cofactor and/or core peptide, where the top and side clamps are drawn together to restrict access to the active site. These data give credence to the proposal that movement of the top and side clamps of up to 14 Å allow for cofactor replenishment and core peptide repositioning during iterative α-*N*-methylation.

Mutation of SonM-R67A produced an inactive enzyme, that when co-crystallized with SonA and SAH, was at once unable to maintain normal contacts with the top clamp, while still binding the cofactor in a manner that allowed the bottom lock to close. The resulting SonM-R67A—SonA-0Me—SAH crystal structure complex thus revealed our only structural data of an unmethylated SonA core peptide. Both the leader and core peptide of unmethylated SonA displayed significant structural changes compared to doubly-methylated SonA (Fig. 7). The unmethylated core peptide is bound as an α-helix within the active site of SonM-R67A in contrast to all other structures that are configured in a defined, stretched out conformation (including the fungal homologs). In addition, helix 5 of the BBD is completely unwound to presumably allow the core peptide to be fully stretched through the active site of SonM-R67A. To help visualize the differences in the structural states observed, we have produced morphing animations of hypothetical transitions between crystallographic snapshots (Supplementary Movies 1 and 2).

Our cumulative results from our model *Shewanella* split borosin pathway allow us to put forth a tentative biogenic proposal for the driving forces governing N-to-C directionality of SonM iterative α-*N*-methylation upon its peptide substrate. We propose the α-helical nature of the unmethylated core peptide, in combination with the degree of helicity of helix 5 of the BBD, positions the core peptide correctly in the active site for catalysis. After the first methylation event, SAH and the singly-methylated SonA core peptide exit from the active site of SonM through opening of the top and side clamps. The newly incorporated α-*N*-methylation at SonA-L63 subsequently disrupts the helicity of the core peptide in close proximity of this residue. α-*N*-Methylations, similarly to prolines, are well-known to disrupt α-helicies^36,40^. The shortened core peptide helix is then repositioned in the active site for the next, more C-terminal methylation site, through compensatory helical formation of helix 5 in the BBD. This trade off in BBD helix 5 formation and core peptide helix disruption, we believe, is driving the N-to-C iterative activity of α-*N*-methylation.

Much work is still necessary to tease out and confirm our proposal concerning the iterative catalytic cycles for borosin *N*-methyltransferases, which we are avidly pursuing through kinetic, structural, and in silico strategies. Nonetheless, this body of work highlights the likely dynamic structural rearrangements and core sequence-specific guidance at play during RiPP biogenesis. In line with this rationale, precursor core sequences and their ensuing conformation restraints have been shown to direct the stereoselectivity of lanthionine bond formation during cytolysin and haloduracin biosynthesis^41,42^. In another example, incorporations of D-configured amino acids into a proteusin RiPP core sequence were proposed to alter the structure and thereby increase solubility of the polytheonamide precursor via a foldase-like mechanism^4^.

All in all, the multiple conformations seen in the split borosin SonA leader peptide hint at a dynamic interplay of structural states to help dictate binding and catalysis. The coming years will undoubtedly shed more light on the intricate strategies and governing forces driving iterative PTM incorporation in RiPPs.

## Methods

### Materials

HiFi DNA Assembly Master Mix, restriction enzymes, phosphatase, OneTaq, and Q5 High Fidelity polymerases were purchased from New England Biolabs (NEB). AspN sequencing grade protease was purchased from Promega. Primers were ordered from IDT (Supplementary Table 2). All genes sequences cloned for this manuscript are found in Supplementary Table 3. Unless otherwise stated, chemicals and reagents were purchased from MilliporeSigma. *Shewanella oneidensis* MR-1 bacteria were given by Dr. Jeffrey Gralnick (University of Minnesota). Images of protein structural models were created using PyMOL 2.3.3. For those containing multiple ray trace modes, images were layered and edited using the software Affinity Designer 1.8.2. Sequence alignments and phylogenetic trees were created using the software Geneious 2019.2.

### Genomic DNA extraction

Genomic DNA from *Shewanella oneidensis* MR-1 was extracted by resuspending cell mass in 600 μL lysis buffer (10 mM Tris pH 8, 1 mM EDTA pH 8, 0.6% SDS, 120 μg/mL proteinase K) and incubating 1 h at 37 °C. An equal volume of phenol:chloroform:isoamyl alcohol (25:24:1 v:v:v) was added and mixed well by inversion. After centrifugation at top speed at room temperature for 5 min, the upper aqueous phase was transferred into a fresh tube. Addition of lysis buffer was repeated until the white protein phase disappeared. Phenol was removed by adding an equal volume of chloroform:isoamyl alcohol (24:1 v:v) to the aqueous layer, mixing by inversion and then spinning at 14,000 × *g* at room temperature for 5 min. The aqueous layer was moved to a fresh tube and DNA was precipitated using ethanol.

### Cloning

All constructs for the heterologous expression of SonM (Uniprot Q8EGW3) and SonA (Uniprot Q8EGW2) proteins in *E. coli* were made using the genes cloned out of *S. oneidensis* MR-1. Q5 polymerase was used to amplify *sonM* and *sonA* genes from the extracted genomic DNA according to the manufacturer’s instructions (Q5 standard buffer used at 1×, 200 μM dNTPs, 0.5 μM each primer, 0.02 U polymerase/50 μL PCR, with 5% DMSO). All constructs were made using HiFi DNA Assembly Master Mix.

To make the s*onM*-*sonA* co-expression construct, pET28b backbone was digested with NcoI-HF and SalI-HF, treated with Antarctic phosphatase, and the band was extracted from an agarose gel using a kit (Thermo Scientific). The native RBS was used in the co-expression construct and an N-terminal hexa-histidine (his_6_) tag was encoded into *sonA*. Gene *sonM* was amplified using primers prmMRJ036_fw and prmMRJ043_rev. Q5 polymerase was used as described above with the following reaction conditions: initial denaturation 30 s at 98 °C; denature 98 °C 10 s, anneal 61.5 °C 30 s, extend 72 °C 25 s for 30 cycles; final extension 72 °C 2 min. Gene *sonA* was amplified with an N-terminal *his*_*6*_ tag using primers prmMRJ044_fw and prmMRJ045_rev in a PCR reaction as follows: initial denaturation 30 s at 98 °C; denature 98 °C 10 s, anneal 57.5 °C 30 s, extend 72 °C 7 s for 30 cycles; final extension 72 °C 2 min. Overlap extension PCR was used to join the *sonM* and *his*_*6*_*-sonA* amplicons as follows: using these two amplicons as DNA template, the first five cycles were allowed to proceed without primers under the following conditions: initial denaturation 30 s at 98 °C; denature 98 °C 10 s, anneal 68 °C 30 s, extend 72 °C 25 s for five cycles; after the fifth cycle, primers prmMRJ036_fw and prmMRJ045_rev were added and the annealing temperature was increased to 72 °C for the remaining 25 cycles, followed by a final extension 72 °C 2 min. The resulting band was excised from an agarose gel before assembly into the backbone. Assembly was transformed into electrocompetent TOP10 *E. coli* cells and colonies were screened via colony PCR using primers T7_fw and T7_rv and OneTaq polymerase. The PCR reaction was set up as follows: standard PCR buffer at 1×, 200 μM dNTPs, 0.2 μM each primer, 1.25 U polymerase/50 μL reaction, with 5% DMSO; initial denaturation 30 s at 94 °C; 30 cycles denature 94 °C 20 s, anneal 46.3 °C 40 s, extend 68 °C 60 s; final extension 68 °C 5 min. Colonies showing a correctly sized band were sequence verified by ACGT.

For expression constructs of the individual genes, *sonM* and *sonA* were amplified from extracted genomic DNA. An N-terminal *his*_*6*_ tag was added to each gene before assembly into the same backbone as the co-expression construct. For *his*_*6*_-*sonM*, prFM1177 and prFM1178 primers were used in a standard Q5 polymerase reaction as follows: initial denaturation 30 s at 98 °C; first five cycles denature 98 °C 7 s, anneal 56.5 °C 15 s, extend 72 °C 20 s; remaining 25 cycles increase annealing temperature to 72 °C; final extension 72 °C 2 min. For *his*_*6*_*-sonA*, prFM1175 and prFM116 primers were used in a standard Q5 polymerase reaction as follows: initial denaturation 30 s at 98 °C; first five cycles denature 98 °C 5 s, anneal 51.5 °C 15 s, extend 72 °C 10 s; remaining 25 cycles increase annealing temperature to 65.5 °C; final extension 72 °C 2 min. PCR products were cleaned up using a kit (Thermo Scientific), assembled with the backbone via HiFi DNA Assembly Master Mix, transformed into TOP10 *E. coli* electrocompetent cells, screened via colony PCR using OneTaq and aforementioned T7 primers, and sequence verified (ACGT) as described above.

Active site mutants of SonM were constructed in the *sonM* and *sonA* co-expression and *sonM* individual expression backgrounds using site directed mutagenesis. Primers prFM1191-prFM1215 were used in appropriate pairs to amplify the entire plasmid under standard Q5 reaction conditions: initial denaturation 30 s at 98 °C; denature 98 °C 10 s, anneal 63.5 °C 20 s, extend 72 °C 3 min for 30 cycles; final extension 72 °C 2 min. The PCR reaction was cleaned up using a kit (Thermo Scientific) and treated with T4 polynucleotide kinase and ligase (NEB) according to manufacturer’s instructions. Subsequent transformation and sequencing was performed as described as above.

Gene *sonA-BBD* (encoding the helical bundle composed of the first 55 amino acids of SonA, 1–55) constructs were assembled into a pET28b empty vector that was digested with NcoI-HF and BamHI, treated with Antarctic phosphatase, and the band was extracted from an agarose gel using a kit (Thermo Scientific). Both inserts described here used plasmid pMF1181 as PCR template DNA. To amplify *his6*-*sonA-BBD*, a standard Q5 PCR was run with primers prFM1175 and pKKC1010: initial denaturation 30 s at 98 °C; denature 98 °C 10 s, anneal 67 °C 30 s, extend 72 °C 20 s for 30 cycles; final extension 72 °C 2 min. To create the co-expression construct of *sonM* and *sonA-BBD*, prmMRJ_036 and pKKC1010 were used in a standard Q5 PCR: initial denaturation 30 s at 98 °C; denature 98 °C 10 s, anneal 72 °C 20 s, extend 72 °C 30 s for 30 cycles; final extension 72 °C 2 min. PCR products were digested with DpnI and cleaned up using a kit (Thermo Scientific), assembled with the backbone via HiFi DNA Assembly Master Mix, transformed into TOP10 *E. coli* electrocompetent cells, screened via colony PCR using OneTaq and aforementioned T7 primers, and sequence verified (ACGT), all as described above.

Both constructs for the heterologous expression of *sspM*_*NRRLS118*_ (NCBI accession WP_031073184.1) and *sspA*_*NRRLS118*_ (NCBI accession WP_031073186.1) in *E. coli* were made using synthesized, codon-optimized genes ordered from Genscript. The codon-optimized genes *sspM*_*NRRLS118*_ and *sspA*_*NRRLS118*_ were amplified as described above with primers to add NdeI and BamHI restriction sites. For *sspM*_*NRRLS118*_, prmMRJ_066_fwd and prmMRJ_067_rev primers were used in a standard Q5 polymerase reaction as follows: initial denaturation 30 s at 98 °C; 30 cycles denature 98 °C 7 s, anneal 62 °C 15 s, extend 72 °C 28 s; final extension 72 °C 2 min. For *sspA*_*NRRLS118*_, prmMRJ_068_fwd and prmMRJ_069_fwd primers were used in a standard Q5 polymerase reaction as follows: initial denaturation 30 s at 98 °C; 30 cycles denature 98 °C 5 s, anneal 65 °C 15 s, extend 72 °C 7 s; final extension 72 °C 2 min. PCR products were cleaned up using a kit (Thermo Scientific) and digested using NdeI and BamHI (NEB). Two plasmid backbones were used: pET28b and pET28_6xHis-gdSUMO (pET28b modified to include a *his*_*6*_ SUMO tag and gifted to us by Dr. Anna Vagstad). The pET28b backbone with a *his*_*6*_ tag and thrombin cleavage site directly upstream of the NdeI restriction site was used for *sspA*_*NRRLS118*_. The pET28_6xHis-gdSUMO backbone containing a his_6_-SUMO tag directly upstream of the NdeI restriction site was used for *sspM*_*NRRLS118*_. The respective plasmids were digested with NdeI, BamHI, and concurrently treated with Antarctic phosphatase (NEB). After digestion, the linearized plasmids were excised and extracted from a 1% agarose gel using a kit (Thermo Scientific). Each insert was ligated into their respective pET28 backbone using T4 ligase (NEB). The assembled constructs were transformed and sequence verified as described above.

### Protein expression and purification

*E. coli* BL21(DE3) cells were transformed with the pET28b expression plasmids and cultured overnight with 50 μg/mL kanamycin at 37 °C. A 10 mL overnight culture was added to 1 L Terrific Broth with 50 μg/mL kanamycin in 2.5 L baffled flasks (Thomson Scientific) with foam stoppers. The 1 L culture was grown to an optical density at 600 nm (OD_600_) of approximately 1. At this time, the cultures were cold-shocked on ice for 30 min followed by induction with 200 μM IPTG (final concentration). After induction, cultures were incubated at 16 °C for 24 h shaking at 250 rpm. Cells were harvested by centrifugation at 5000 × *g* for 30 min at 4 °C. Cell pellets were resuspended in ice-cold lysis buffer (50 mM HEPES pH 8, 300 mM NaCl, 10% (v/v) glycerol) with 20 mM imidazole, and lysed using 1 mg/mL (final concentration) lysozyme for 30 min at 4 °C followed by sonication. The resultant lysate was clarified by centrifugation at 15,000 × *g* for 30 min at 4 °C. Benchtop or FPLC affinity purifications were used for all proteins and both methods yielded protein with equivalent activity and purity. For benchtop purifications, supernatant was incubated with Ni-NTA beads (Gold Bio) on a rotator at 4 °C for 1 h. Beads were washed with ten column volumes of ice-cold lysis buffer and protein was eluted in lysis buffer containing 250 mM imidazole. For FPLC affinity purification, a pre-packed HisTrap 5 mL column (GE) was used: the supernatant was filtered with a 0.2 µm syringe filter before being loaded onto the pre-equilibrated column. After loading, the column was washed with five column volumes of lysis buffer with 20 mM imidazole and protein was eluted using lysis buffer with 250 mM imidazole. For benchtop and FPLC purifications, protein-containing fractions were collected and concentrated using Amicon Ultra filters (10-kDa MWCO) and subsequently loaded onto a HiLoad 16/600 Superdex 200 pg size exclusion column (GE) pre-equilibrated with lysis buffer. A flow rate of 1 mL/min was used. Fractions were again collected and concentrated using Amicon Ultra filters. Concentrations were determined using the BioRad Bradford assay. For his_6_-SonM and his_6_-SonA proteins to be used in kinetics assays, samples were flash frozen in liquid nitrogen and stored at −80 °C. For proteins to be used in crystallography, samples were concentrated to approximately 20 mg/mL and dialyzed into 10 mM HEPES pH 8 to de-salt and remove glycerol. Samples were divided into 40 µL aliquots, flash frozen in liquid nitrogen, and stored at −80 °C until needed.

### Mass spectrometry

Heterologously expressed and purified protein was prepared for mass spectrometric analysis by an in-gel digest method^24^. Briefly, the band corresponding to his_6_-SonA was extracted from an SDS-PAGE gel, cut into ~2 mm × 2 mm pieces and placed in 1.5 mL LoBind tubes (Eppendorf). Gel cubes were then washed with a 1:1 ratio of 100 mM ammonium bicarbonate (ABC): acetonitrile (ACN) three times until gel pieces appeared clear. After dye removal, they were then dehydrated in 100% ACN until semi-opaque (~30 s), and the ACN was subsequently discarded. After rehydration in digest buffer (50 mM ABC and 1:50 units AspN protease (Promega)), gel pieces were placed on ice for 15 min and then were transferred to a 37 °C incubator overnight. The next day, excess liquid from the digest was collected and transferred to a new LoBind tube. Digested peptides were extracted from the gel pieces by first covering them with 60 µL of 50% ACN and 0.3% formic acid (FA) and incubating at room temperature for 15 min. After this incubation, the supernatant was recovered. This extraction was repeated with 60 µL of 80% ACN and 0.3% FA and the supernatant was recovered and placed into the same LoBind tube. The pooled peptide extractions were frozen at −80 °C for 30 min to deactivate the protease. After freezing, the extracted peptides were thawed and dried using a SpeedVac (Eppendorf). Dried peptides were reconstituted in 0.1% FA and purified/desalted using C18 ZipTips according to the manufacturer’s instructions. Purified and desalted peptides were again dried using the SpeedVac and then reconstituted in 15–30 µL of 20% ACN, 0.1% FA, and transferred to glass vials for MS analysis. Peptide mass spectrometric analysis (LC-MS/MS HCD) LC-MS/MS measurements of digested peptides was performed^30^. Briefly, data were obtained on a Thermo Scientific Fusion mass spectrometer furnished with a Dionex Ultimate 3000 UHPLC system with a nLC column (200 mm × 75 μm) packed with Vydac 5-μm particles of 300 Å pore size (Hichrom Limited). The LC method used the following solvents: 0.1% FA in water (solvent A) and 0.1% FA in acetonitrile (solvent B). After a 4.5 min equilibration of 20% solvent B at a flow rate of 1 μL/min, the flow rate was dropped to 0.3 μL/min over 0.5 min. The sample was then injected and the gradient run was as follows: solvent B at 20–85% for 32 min, 85% 2 min. Mass spectra were acquired in positive-ion mode. Full MS was done at a resolution of 60,000 [automatic gain control (AGC) target, 4 × 105; maximum ion trap (IT), 50 ms; range, 300–1800 *m*/*z*], and data-dependent and targeted MS/MS were both performed at a resolution of 15,000 (AGC target, 5 × 105; maximum IT, 500 ms; isolation window, 2.2) using higher-energy collisional dissociation (HCD). HCD collision energies from 14 to 20% with steps of ±4% were used during LC-MS/MS measurements. Data were processed and analyzed using Thermo Fisher Xcalibur 3.0.63 software and MaxQuant 1.5.3.30^2^. Protein his_6_-SspA_NRRLS118_ was prepared for and analyzed by LC-MS/MS in a manner identical to the method described above.

### Kinetics assay

Plasmids for expressing *S*-adenosylhomocysteine nucleosidase (SAHN; Uniprot P0AF12) and adenine deaminase (ADE; Uniprot P31441) with N-terminal *his*_*6*_ tags were acquired from the ASKA collection^43^. The gene for SAHN was expressed and purified as above with the addition of 1 mM DTT in all buffers. During the expression of ADE, to replace the Fe^2+^ metal with Mn^2+^ in the active site, 20 µM 2,2′-dipyridyl and 1.0 mM MnCl_2_ were added to the media at the time of induction^44^. Other expression and purification steps for ADE were carried out in the same manner as for SAHN. Glutamate dehydrogenase (GDH) and ammonia assay reagent were used from the Ammonia Detection Kit (Millipore Sigma AA0100) according to previously established methods^34^. For use in the kinetics assays, SAM was purified by HPLC using a BUCHI PrepChrom C-700 instrument and BUCHI FlashPure EcoFlex C18 Column (140000048). The solvents used for HPLC: 0.1% FA in water (solvent A) and ACN (solvent B) at a flow rate of 10 mL/min. The linear gradient used: solvent B at 5% for 0.5 min, 5–95% for 15 min, and 95% for 2 min. SAM was purified to ~97–98.5% purity when measured by our assay. Kinetic experiments were conducted in a clear, flat-bottomed 96-well plate (Sarstedt 82.1581.001) in a SpectraMax ID5 (Molecular Devices, Inc). Methyl transfer was measured by monitoring the decrease in absorbance at 340 nm (corresponding to the loss of NADPH in the coupled-enzyme assay). Three technical replicates were performed for each varied substrate concentration, and reads were taken every 30 or 40 s. For the experiments varying SonA, a saturating concentration of SAM (1 mM) was assayed with 2, 4, 8, 15, 25, 40, 60, 100, and 200 μM SonA. For the experiments varying SAM, a saturating concentration of SonA (100 μM) was assayed with 10, 50, 100, 300, 500, 800, and 1000 μM SAM to define the substrate-velocity curve. All kinetic experiments with wt SonM used a final concentration of 5 μM enzyme. Upon assembling all assay components except the methyltransferase in the plate wells, absorbance values were collected for 10–15 min after which the methyltransferase was added to start the reaction. The absorbance data was used to calculate the concentration of NADPH at each time point with Beers’ Law and the reported extinction coefficient of NADPH, ε340 = 6220 M^−1^ cm^−1^. The concentration of the final reading before addition of the methyltransferase was used as baseline for all successive concentration values, making the curve reflect product formation over time. The slope was taken over the linear range of this curve, giving the velocity of product formation (µM/min). The velocity of three negative control replicates (lacking the varied substrate) were averaged and subtracted from the velocity of each individual replicate to account for background SAM degradation. These velocity values were then divided by the enzyme concentration used giving the rate of product formation (min^−1^) and plotted with their respective substrate concentrations in GraphPad Prism 8 to produce the substrate-velocity curve. A non-linear regression analysis was used to fit the data to the Michaelis–Menten equation and give values for the desired kinetic constants, *V*_max_, *k*_cat_, and *K*_M_ or *K*_i_, where appropriate. All SonM mutants run in the kinetics assay were verified by LC-MS/MS to only methylate positions SonA-MLE63 and SonM-IML65 when methylation was observed (Supplementary Fig. 25).

For the collection of data for the kinetic modeling of 0, 1, and 2 methylated species of his_6_-SonA (SonA-0Me, SonA-1Me, and SonA-2Me, respectively) the reactions were prepared as described above using 5 μM SonM, 100 μM SonA, and 1 mM SAM. Duplicates of each reaction time point to be analyzed by mass spectrometry were measured using the plate reader prior to quenching the reactions in SDS-dye and boiling for 5 min. SonA was then prepared for mass spectrometry analysis following the procedure described above. After reconstitution in 30 μL, the samples were further diluted 200-fold. The LC method was also modified to a flow rate of 1 μL/min: solvent B at 20% for 5 min, 20–85% for 15 min, and 85% for 2 min. Mass spectra were acquired and analyzed using the methods described in the mass spectrometry section.

### In vitro methylation of his_6_-SspA_NRRLS118_ by SspM_NRRLS118_

Methylation of his_6_-SspA_NRRLS118_ by SspM_NRRLS118_ was performed via an in vitro reaction containing 40 µM his_6_-SspA_NRRLS118_, 8 µM SspM_NRRLS118_, 1000 µM SAM, and 8 µM SAHN in 50 mM HEPES buffer pH 8. The reaction was quenched after 15 min by adding SDS-dye and immediately boiling the sample for 5 min. This was followed by preparation of his_6_-SspA_NRRLS118_ for mass spectrometry analysis as described previously.

### Kinetic modeling of observed methylated species of SonA

A computational simulation was used to evaluate which parameters would be consistent with the dynamics that were observed. Specifically, a reaction of the following form was considered:

1 $$E+S\,\mathop{\leftrightarrow}^{{k}_{1}}\ ES\,\mathop{\leftrightarrow}^{{k}_{2}}\ E+{P}_{1}\,\mathop{\leftrightarrow}^{{k}_{3}}\ EP\, \mathop{\leftrightarrow}^{{k}_{4}}\ E+{P}_{2}$$

In this case *S* represents free substrate (SonA), *P*_1_ is the intermediate product (SonA-1Me), and *P*_2_ is the final product (SonA-2Me). *E* represents free enzyme (SonM), while *ES* and *EP* represent enzyme bound to substrate or the intermediate product. These dynamics were simulated using the following series of ordinary differential equations:

2 $$\frac{{{{{{{\rm{d}}}}}}E}}{{{{{{{\rm{d}}}}}}t}}=\; -{k}_{1}\,* E* S+{k}_{1r}* {ES}+{k}_{2}* {ES}-{k}_{2r}* E* {P}_{1}-{k}_{3}* E* {P}_{1}\\ +{k}_{3r}* {EP}+{k}_{4}* {EP}-{k}_{4r}* E* {P}_{2},$$

3 $$\frac{{{{{{{\rm{d}}}}}}ES}}{{{{{{{\rm{d}}}}}}t}}\,=\,{k}_{1}* E* S-{k}_{1r}* {ES}-{k}_{2}* {ES}+{k}_{2r}* E* {P}_{1},$$

4 $$\frac{{{{{{{\rm{d}}}}}}EP}}{{{{{{{\rm{d}}}}}}t}}\,=\,{k}_{3}* E*{P}_{1}-{k}_{3r}*{EP}-{k}_{4}* {EP}+{k}_{4r}* E* {P}_{2},$$

5 $$\frac{{{{{{{\rm{d}}}}}}S}}{{{{{{{\rm{d}}}}}}t}}=-{k}_{1}* E* S+{k}_{1r}* {ES},$$

6 $$\frac{{{{{{\rm{d}}}}}}{P}_{1}}{{{{{{{\rm{d}}}}}}t}}\,=\,{k}_{2}* {ES}-{k}_{2r}* E* {P}_{1}-{k}_{3}* E* {P}_{1}+{k}_{3r}* {EP},$$

7 $$\frac{{{{{{\rm{d}}}}}}{P}_{2}}{{{{{{{\rm{d}}}}}}t}}={k}_{4}* {EP}-{k}_{4r}* E* {P}_{2}$$

The rate at which each reaction proceeds in the forward direction is *k*_n_, while the reverse rate of the reaction is *k*_nr_. The parameter values that were used for our base model can be found in Supplementary Table 4. Modeling was done in R version 3.6.2. The model was solved using the deSolve 1.28 package in R. Simulations were run for 3600 s by 0.1 s timesteps. Code to run simulations is provided in Supplementary Data 1.

### Protein crystallization

After purification, concentration, and dialysis into 10 mM Hepes pH 8 buffer as described above, proteins at 20 mg/mL (as measured by Bradford assay) were sterile filtered and screened for precipitation and crystal formation using the JCSG+ Suite (Qiagen) at 292 K. For each condition, three precipitant:protein ratios were tested (1:1, 1:2, and 1:3). Screens were conducted by the Nanoliter Crystallization Facility at the University of Minnesota (Minneapolis, MN). For the SonM—SonA complex, the best condition was identified as 20% polyethylene glycol (PEG) 3350 with 240 mM sodium malonate at pH 7. This condition was further refined for pH (5.5–7) and PEG 3,350 concentration (0–20%). These conditions were also used for crystallizing all of SonM active site mutants crystallized in complex with SonA. For the SonM—SonA-BBD complex, the best condition was identified as 100 mM Bis–Tris at pH 5.5 with 100 mM ammonium acetate and 17% PEG 10,000. This condition was further refined for pH (5–5.5) and PEG concentration (4–7%). Diffraction-quality crystals were visible at 292 K in 1 day for all crystals except SonM-R67A—SonA-0Me—SAH, which produced crystals in 3 days. SAH was dissolved in the mother liquor to a concentration of 5 mM for co-crystallization or 1 mM SAM was added to protein solutions before drops were set. Crystals were cryoprotected by transferring to a drop consisting of the mother liquor supplemented with 20% PEG and 20% glycerol.

### Data collection, structure resolution, and refinement

X-ray diffraction datasets were collected at 100 K using synchrotron radiation on the 23-IDB beamline (Supplementary Table 5) at the Advanced Photon Source (APS, Argonne, Illinois, USA). The integration and the scaling of the X-ray diffraction data were performed using the XDS package version Mar 15, 2019^45^. Data were processed in P2_1_ space group for all but the SonM-R67A—SonA-0Me—SAH structure (P422). Molecular replacement was performed using the OphMA structure as a model (PDB: 5N0P [10.2210/pdb5N0P/pdb]) (33% sequence identity) using PHASER 2.8.3^46^. Manual model building was performed and improved using Coot 0.8.9.2^47^. Cycles of refinement were performed using REFMAC 5.8.0238^48^. Final refinement statistics are shown in Supplementary Table 5.

### Reporting summary

Further information on research design is available in the Nature Research Reporting Summary linked to this article.

## Supplementary information

Supplementary Information

Supplementary Movie 1. Animated transition between SonM—SonA-2Me—SAH and SonM—SonA-BBD—(±)SAM complexes.

Supplementary Movie 2. Animated transition between SonM—SonA-2Me—SAH and SonM-R67A—SonA-0Me—SAH complexes.

Supplementary Dataset 1

Description of additional supplementary files

Reporting Summary

## Data Availability

The mass spectrometry data and custom R code for the kinetics simulations generated in this study have been deposited in the Data Repository for the University of Minnesota (10.13020/y8ry-gm18)^49^. Crystallographic data generated in this study are deposited in the Protein Data Bank: 7LTE [10.2210/pdb7LTE/pdb] (SonM—SonA-2Me—SAH); 7LTC [10.2210/pdb7LTC/pdb] (SonM—SonA-2Me); 7LTF [10.2210/pdb7LTF/pdb] (SonM-Y58F—SonA-2Me); 7LTH [10.2210/pdb7LTH/pdb] (SonM-Y93F—SonA-2Me); 7LTS [10.2210/pdb7LTS/pdb] (SonM-R67A—SonA-0Me—SAH); 7LTR [10.2210/pdb7LTR/pdb] (SonM—SonA-BBD—(±)SAM). Additional data in this study are provided in the Supplementary Information. For Supplementary Figs. 6, 14, and 17, source data are provided with this paper. All other materials and data supporting the results of this study can be requested from the corresponding authors.

## Source data

source data

## Code availability

Code for the kinetic modeling in this study can be found in Supplementary Data 1 and was also deposited in the Data Repository for the University of Minnesota (10.13020/y8ry-gm18).
