# Supplementary material for: Conformational rearrangements enable iterative backbone N-methylation in RiPP biosynthesis
Source: Nat Commun. 2021 Sep 9;12:5355. doi: 10.1038/s41467-021-25575-7 (PMC8429565; doi:10.1038/s41467-021-25575-7)
Supplement: Supplementary file 1 — Supplementary Information [file 41467_2021_25575_MOESM1_ESM.pdf]

Supplementary Information for

Conformational rearrangements enable iterative backbone *N*-methylation in RiPP biosynthesis

Fredarla S. Miller,[1†] Kathryn K. Crone,[1†] Matthew R. Jensen,[2,3] Sudipta Shaw,[1] William R. Harcombe,[2,4] Mikael H. Elias,[1,2*] and Michael F. Freeman[1,2*]

[1] Department of Biochemistry, Molecular Biology, and Biophysics, the [2] BioTechnology Institute, and the [4] Department of Ecology, Evolution and Behavior, University of Minnesota-Twin Cities, St. Paul, Minnesota, USA

[3] Present address: Science Department, Concordia University-St. Paul, St. Paul, Minnesota, USA

[†] These authors contributed equally to this work: F.S. Miller and K.K. Crone

[*] Correspondence and requests for materials should be addressed to M.H. Elias (email: mhelias@umn.edu) or M.F. Freeman (mffreema@umn.edu)

Table of Contents

Supplementary Table 1. Split borosins encoded in NCBI representative genomes from the genus *Shewanella*. 3
Supplementary Table 2. All primers and plasmids used in this manuscript. 4
Supplementary Table 3. All genes used in this manuscript. 5
Supplementary Table 4. Parameter values used in our base kinetics model. 8
Supplementary Table 5. Crystallographic data and statistics. 9
Supplementary Figure 1. Phylogenic tree of borosin methyltransferase domains including putative split borosin domains encoded in bacteria. 10
Supplementary Figure 2. Geneious sequence alignment of previously identified fungal borosin and putative bacterial split borosin methyltransferase domains. 11
Supplementary Figure 3. Gene clusters of the two split borosins analyzed in this study. 15
Supplementary Figure 4. Mass spectrometric analysis of split borosin coexpressions. 16
Supplementary Figure 5. Global structural comparisons of borosin α-*N*-methylating enzymes. 27
Supplementary Figure 6. Oligomeric state of his$_6$-SonM and his$_6$-SonA proteins by size exclusion chromatography. 28
Supplementary Figure 7. Structural overlay of SonM—SonA-2Me—SAH. 29
Supplementary Figure 8. Structural overlay of the BBD with other protein domains. 30
Supplementary Figure 9. Thermal motion B-factors for SonA and SonM. 31
Supplementary Figure 10. SonM—SonA-2Me—SAH active site coordination. 32
Supplementary Figure 11. SonM interactions with the SonA core peptide in SonM—SonA-2Me—SAH. 33
Supplementary Figure 12. Superposition of SonM—SonA-2Me complexes. 34
Supplementary Figure 13. SAH makes extensive contacts in the SonM—SonA-2Me—SAH complex. 35
Supplementary Figure 14. SonM – fitted Michaelis-Menten kinetic curves. 36
Supplementary Figure 15. Active site tyrosine mutant structures. 38
Supplementary Figure 16. SonM in vitro reactions analyzed by LC-MS/MS and compared to kinetic model simulations. 39
Supplementary Figure 17. SonM – fitted Michaelis-Menten competitive inhibition kinetic curves. 40
Supplementary Figure 18. Superposition of SonM—SonA-2Me and SonM—SonA-BBD—(±)SAM complexes. 41
Supplementary Figure 19. Bottom lock configurations of the SonM—SonA-BBD—(±)SAM complex. 42
Supplementary Figure 20. Top lock configurations between borosin α-*N*-methyltransferases. 43
Supplementary Figure 21. Active site configuration differences among split borosin complexes. 44
Supplementary Figure 22. Interaction networks in different configurations of SonM and mutant complexes. 45
Supplementary Figure 23. Structural differences in the heterodimers of SonM-R67A—SonA-0Me—SAH. 46
Supplementary Figure 24. Structural differences in the BBD of SonM-R67A—SonA-0Me—SAH and *apo* SonM—SonA-2Me—SAH. 47
Supplementary Figure 25. Mass spectrometric analysis of SonM mutant in vitro reactions. 48
References 55

Supplementary Table 1. Split borosins encoded in NCBI representative genomes from the genus *Shewanella*.

	Representative species	Genome assembly	Gene cluster	Putative split borosin methyltransferase Protein ID	Putative split borosin precursor Protein ID	Syntenic with *S. oneidensis* MR-1 cluster (Y/N)
1	*Shewanella aestuarii* strain PN3F2	GCA_011765625.1 ASM1176562v1	1	WP_167676051.1	WP_167676053.1	Y
2	*Shewanella algae* strain MARS 14	GCA_000947195.1	1	WP_044735032.1	WP_028781613.1	N
3	*Shewanella algidipiscicola* strain LMG 23746	GCF_003605125.1_ASM360512v1	1	WP_119977437.1	WP_110456456.1	Y
4	*Shewanella amazonensis* SB2B	NC_008700	1	WP_011760496.1	WP_011760495.1	Y
			2	WP_011758902.1	WP_011758901.1	N
5	*Shewanella atlantica* strain HAW-EB5	GCF_003966265.1_ASM396626v1	1	WP_126503733.1	WP_126503731.1	Y
			2	WP_126507131.1	WP_126507132.1	N
6	*Shewanella baltica* OS678	GCF_000178875.2_ASM17887v2	1	WP_006085112.1	WP_006085113.1	Y
7	*Shewanella benthica* KT99	GCF_000172075.1_ASM17207v1	1	WP_005497745.1	WP_005497743.1	Y
8	*Shewanella bicestrii* strain JAB-1	GCF_002216875.1_ASM221687v1	1	WP_089067321.1	WP_086904596.1	Y
9	*Shewanella canadensis* strain HAW-EB2	GCF_003966225.1_ASM396622v1	1	WP_126520857.1	WP_126520690.1	Y
			2	WP_126518905.1	WP_126518907.1	N
10	*Shewanella carassii* strain 08MAS2251	GCA_002777975.1 ASM277797v1	-	-	-	-
11	*Shewanella chilikensis*	GCF_003217175.1_ASM321717v1	-	-	-	-
12	*Shewanella colwelliana* ATCC 39565	GCF_000518705.1_ASM51870v1	1	WP_051413204.1	WP_028765344.1	Y
13	*Shewanella corallii* strain A687	GCF_003353085.1_ASM335308v1	1	WP_115137829.1	WP_115137830.1	Y
14	*Shewanella decolorationis* S12	GCF_000485795.1_SheDec2.0	1	WP_039978563.1	WP_006085113.1<br>WP_023268149.1	Y
15	*Shewanella denitrificans* OS217	NC_007954	1	WP_041405716.1	WP_041405717.1	Y
			2	WP_011495401.1	WP_011495402.1<br>WP_041405677.1	N
			3	WP_011495261.1	WP_011495262.1<br>WP_157599827.1<br>WP_041405665.1	N
16	*Shewanella donghaensis* strain LT17	NZ_CP041783	1	WP_144213469.1	WP_144213471.1	Y
			2	WP_144214053.1	WP_144206800.1<br>WP_144206802.1	N
17	*Shewanella fidelis* ATCC BAA-318	GCF_000518605.1_ASM51860v1	1	WP_028768597.1	WP_028768598.1<br>WP_028768599.1	Y
			2	WP_037410318.1	WP_028766355.1	N
18	*Shewanella fodinae* strain 74A	GCF_004342405.1_ASM434240v1	1	WP_133038992.1	WP_133038991.1	Y
			2	WP_133039724.1	-	N
19	*Shewanella frigidimarina* NCIMB 400	NC_008345	1	WP_049763522.1	WP_011636573.1	Y
20	*Shewanella hafniensis* isolate NILIPAHB1	GCA_902728295.1_NILIPAHB_1	1	CAA7314325.1	CAA7314324.1	Y
21	*Shewanella halifaxensis* HAW-EB4	NC_010334	1	WP_012276456.1	WP_012276457.1	Y
22	*Shewanella hanedai* strain JCM 20706	GCF_007197645.1_ASM719764v1	1	WP_143563155.1	WP_143563156.1	Y
23	*Shewanella indica*	GCF_002836975.1_ASM283697v1	-	-	-	-
24	*Shewanella japonica* strain KCTC 22435	NZ_CP020472	1	WP_080916319.1	WP_080916317.1	Y
			2	WP_080915084.1	WP_080915085.1	N
25	*Shewanella khirikhana* strain TH2012	GCA_003957745.1_ASM395774v1	1	AZQ11249.1	AZQ11248.1	Y
			2	AZQ09459.1	AZQ09458.1	N
26	*Shewanella litoralis* strain JCM 32306	GCF_009828585.1_ASM982858v1	1	WP_160053732.1	WP_160053733.1	Y
27	*Shewanella livingstonensis* strain LMG 19866	NZ_CP034015	1	*disrupted*	WP_124730162.1	Y
28	*Shewanella loihica* PV-4	NC_009092	1	WP_011865074.1	WP_011865075.1	Y
29	*Shewanella mangrovi* strain YQH10	GCF_000753795.1_ASM75379v1	1	WP_037443456.1	WP_037443455.1	N
30	*Shewanella marina* JCM 15074	GCF_000614975.1_ASM61497v1	-	-	-	-
31	*Shewanella marisflavi* strain EP1	NZ_CP022272	1	WP_088905093.1	WP_088905092.1	Y
32	*Shewanella maritima* strain D4-2	NZ_CP036200	1	WP_130597927.1	WP_130597926.1	Y
			2	WP_130598048.1	WP_130598049.1	N
33	*Shewanella morhuae* strain ATCC BAA-1205	GCF_900156405.1_IMG-taxon_2681812898	1	WP_076497071.1	WP_006085113.1<br>WP_076496897.1	Y
34	*Shewanella oneidensis* MR-1	GCF_000146165.2_ASM14616v2	1	WP_011071665.1	WP_011071666.1	Y
35	*Shewanella pealeana* ATCC 700345	NC_009901	1	WP_012154539.1	WP_012154541.1	Y
36	*Shewanella piezotolerans* WP3	NC_011566	-	-	-	-
37	*Shewanella polaris* strain SM1901	NZ_CP041036	1	WP_140234809.1	WP_140234808.1	Y
38	*Shewanella psychrophila* strain WP2	NZ_CP014782	1	WP_077753941.1	WP_077753940.1	Y
			2	WP_077754459.1	WP_077754460.1<br>WP_077754461.1	N
39	*Shewanella putrefaciens* CN-32	NC_009438	1	WP_011918927.1	WP_011918928.1	Y
40	*Shewanella sediminis* HAW-EB3	NC_009831	1	WP_041421581.1	WP_012141759.1	Y
			2	WP_012143903.1	WP_012143904.1	N
41	*Shewanella vesiculosa* LMG 24424	GCF_003797885.1_ASM379788v1	1	WP_124016834.1	WP_124016835.1	Y
42	*Shewanella violacea* DSS12	NC_014012	1	WP_013050640.1	WP_013050641.1	Y
43	*Shewanella waksmanii* ATCC BAA-643	GCF_000518805.1_ASM51880v1	1	WP_028771625.1	WP_028771626.1	Y
			2	WP_051484558.1	WP_028773238.1<br>WP_028773237.1	N
44	*Shewanella woodyi* ATCC 51908	NC_010506	1	WP_041418034.1	WP_012324159.1	Y
45	*Shewanella xiamenensis* strain T17	GCF_001723195.1_ASM172319v1	1	WP_039978563.1	WP_006085113.1<br>WP_037425540.1	Y

Supplementary Table 2. All primers and plasmids used in this manuscript.

Primers used in this study	
**Name**	**Sequence (5'-3')**
prmMRJ036_fw	ACTTTAAGAAGGAGATATACCATGGGATCACTCGTCTGTG
prmMRJ043_rev	GATGATGATGATGATGCATGTTTTCTCCTTATTGTTAATAATGATTCAATAAC
prmMRJ044_fw	AGGAGAAAACATGCATCATCATCATCATCACATGTCTGGATTATCGGATTTT TTTAC
prmMRJ045_rev	CGAGTGCGGCCGCAAGCTTGTCGACTTAATCACCATTACCATGTG
T7_fw	TAATACGACTCACTATAGGG
T7_rv	GCTAGTTATTGCTCAGCGG
prFM1175	TTTAAGAAGGAGATATACATGCATCATCATCATCAT
prFM1176	AGTGCGGCCGCAAGCTTGTTAATCACCATTACCATG
prFM1177	TAAGAAGGAGATATACATGCATCATCATCATCATCACAGCAGCATGGGATCA CTCGTC
prFM1178	AGTGCGGCCGCAAGCTTGTTATCCCAAATCTTCGGG
prFM1191	GAAGTTAAAAATAAACGAGACACCTACGA
prFM1192	GAAGTTAAAAATGCCCGAGACACCTAC
prFM1193	ACCATTTTGCGCATAAAACTGCTG
prFM1194	CGAGACACCTTCGAGCAAATGGTC
prFM1195	GCGATTTTTAACTTCACCATTTTGCG
prFM1212	GCAGCAGTTTTTTGCGCAAAA
prFM1213	AAATTGATGACATTGGGGTTGAGC
prFM1214	TGTGCACTCTTCGGTCATCC
prFM1215	CACGGTTTTTTTACCCGCTCTC
prKKC1010	GAGCTCGAATTCGGATCTTAACCACTTAACGT
prmMRJ_066_fwd	ATATAACATATGCAGGAGACCACCG
prmMRJ_067_rev	TTATATGGATCCTTAACGACGCGCCG
prmMRJ_068_fwd	ATATAACATATGCCGGCGGC
prmMRJ_069_rev	TTATATGGATCCTTACGCACCGCTCGG
**Plasmids used in this study**	
**ID**	**Description**
pMF1181	SonM-gRBS-His-SonA_pET28b
pMF1235	His-SonA_pET28b
pMF1236	His-SonM_pET28b
pMF1230	His-ADE (JW_3640 ASKA collection)
pMF1231	His-SAHN (JW_0155 ASKA collection)
pMF1256	SonM-R67A-gRBS_His-SonA_pET28b
pMF1257	His-SonM-R67A_pET28b
pMF1258	SonM-R67K-gRBS_His-SonA_pET28b
pMF1259	His-SonM-R67K_pET28b
pMF1260	SonM-Y71F-gRBS_His-SonA_pET28b
pMF1261	His-SonM-Y71F_pET28b
pMF1263	SonM-Y58F-gRBS_His-SonA_pET28b
pMF1264	His-SonM Y58F_pET28b
pMF1265	SonM-Y58F-Y71F-gRBS_His-SonA_pET28b
pMF1266	His-SonM Y58F + Y71F_pET28b
pMF1267	SonM-Y93F-gRBS_His-SonA_pET28b
pMF1268	His-SonM Y93F_pET28b
pMF1269	His-SonA_helicalbundle_pET28b
pMF1283	SonM-gRBS-His-SonA_helical bundle_pET28b
pMF1197	pET28b-His-StrA

Supplementary Table 3. All genes used in this manuscript.

UniProt ID	Name	Description
P31441	*ade*	Adenine deaminase
ATGAATAATTCTATTAACCATAAATTTCATCACATTAGCCGGGCTGAATACCAGGAATTGTTAG<br>CCGTTTCCCGTGGCGACGCTGTTGCCGATTATATTATTGATAATGTCTCTATTCTCGACCTGAT<br>CAATGGCGGAGAAATTTCCGGCCCAATTGTGATTAAAGGACGTTACATTGCCGGTGTTGGCGC<br>AGAATACACTGATGCTCCGGCTTTGCAGCGGATTGATGCTCGCGGCGCAACGGCGGTGCCAG<br>GGTTTATTGATGCTCACCTGCATATTGAATCCAGCATGATGACGCCGGTCACTTTTGAAACCG<br>CTACCCTGCCGCGCGGCCTGACGACCGTTATTTGCGACCCTCATGAAATCGTCAACGTGATG<br>GGCGAAGCCGGATTCGCCTGGTTTGCCCGCTGTGCCGAACAGGCAAGGCAAAACCAGTACTT<br>ACAGGTCAGCTCTTGCGTACCCGCCCTGGAAGGCTGCGATGTTAACGGTGCCAGTTTTACCC<br>TTGAACAGATGCTCGCCTGGCGGGACCATCCGCAGGTTACCGGCCTTGCAGAAATGATGGAC<br>TACCCTGGCGTAATTAGCGGGCAGAATGCGCTGCTCGATAAACTGGATGCATTTCGCCACCT<br>GACGCTGGACGGTCACTGCCCGGGTTTGGGTGGTAAAGAACTTAACGCCTATATTACTGCGG<br>GTATTGAAAACTGCCACGAAAGTTATCAGCTGGAAGAAGGACGCCGGAAATTACAACTCGGCA<br>TGTCGTTGATGATCCGCGAAGGGTCCGCTGCCCGCAATCTCAACGCGCTGGCACCGTTGATC<br>AACGAATTTAACAGCCCGCAATGCATGCTCTGTACCGATGACCGTAACCCGTGGGAGATCGC<br>CCATGAAGGACACATCGATGCCTTAATTCGCCGCCTGATCGAACAACACAATGTGCCGCTGCA<br>TGTGGCATATCGCGTCGCCAGCTGGTCGACGGCGCGCCACTTTGGTCTGAATCACCTCGGCT<br>TACTGGCACCCGGCAAGCAGGCCGATATCGTCCTGTTGAGCGATGCGCGTAAGGTCACGGTG<br>CAGCAGGTACTGGTGAAAGGCGAGCCGATTGATGCGCAAACCTTACAGGCGGAAGAGTCGG<br>CGAGACTGGCACAATCCGCTCCGCCATATGGCAACACCATTGCCCGCCAGCCAGTTTCCGCC<br>AGCGACTTTGCCCTGCAATTTACGCCCGGAAAACGCTATCGGGTCATTGACGTCATCCATAAC<br>GAATTGATTACGCACTCCCACTCCAGCGTCTACAGCGAAAATGGTTTTGATCGCGATGATGTG<br>AGCTTTATTGCCGTACTTGAGCGTTACGGGCAACGGCTGGCTCCGGCTTGTGGTTTGCTTGG<br>CGGCTTTGGACTGAATGAAGGTGCGCTGGCTGCGACGGTCAGCCATGACAGCCATAATATTG<br>TGGTGATCGGTCGCAGTGCCGAAGAGATGGCGCTGGCGGTCAATCAGGTGATTCAGGATGG<br>CGGCGGGCTGTGCGTGGTACGTAACGGCCAGGTACAAAGTCATCTGCCGTTACCCATTGCCG<br>GGCTGATGAGCACCGACACGGCGCAGTCGCTGGCGGAACAAATTGACGCCTTGAAAGCCGC<br>CGCCCGTGAATGCGGTCCGTTACCCGATGAGCCGTTTATTCAGATGGCGTTTCTTTCTCTGCC<br>AGTGATCCCCGCGCTAAAACTAACCAGTCAGGGGCTATTTGATGGCGAGAAGTTTGCCTTCAC<br>TACGCTGGAAGTCACGGAATAA		
P0AF12	*sahn*	*S*-adenosylhomocysteine nucleosidase
ATGAAAATCGGCATCATTGGTGCAATGGAAGAAGAAGTTACGCTGCTGCGTGACAAAATCGAA<br>AACCGTCAAACTATCAGTCTCGGCGGTTGCGAAATCTATACCGGCCAACTGAATGGAACCGAG<br>GTTGCGCTTCTGAAATCGGGCATCGGTAAAGTCGCTGCGGCGCTGGGTGCCACTTTGCTGTT<br>GGAACACTGCAAGCCAGATGTGATTATTAACACCGGTTCTGCCGGTGGCCTGGCACCAACGT<br>TGAAAGTGGGCGATATCGTTGTCTCGGACGAAGCACGTTATCACGACGCGGATGTCACGGCA<br>TTTGGTTATGAATACGGTCAGTTACCAGGCTGTCCGGCAGGCTTTAAAGCTGACGATAAACTG<br>ATCGCTGCCGCTGAGGCCTGCATTGCCGAACTGAATCTTAACGCTGTACGTGGCCTGATTGTT<br>AGCGGCGACGCTTTCATCAACGGTTCTGTTGGTCTGGCGAAAATCCGCCACAACTTCCCACA<br>GGCCATTGCTGTAGAGATGGAAGCGACGGCAATCGCCCATGTCTGCCACAATTTCAACGTCC<br>CGTTTGTTGTCGTACGCGCCATCTCCGACGTGGCCGATCAACAGTCTCATCTTAGCTTCGATG<br>AGTTCCTGGCTGTTGCCGCTAAACAGTCCAGCCTGATGGTTGAGTCACTGGTGCAGAAACTTG<br>CACATGGCTAA		
Q8EGW3	*sonM* (SO1478)	Borosin methyltransferase
ATGGGATCACTCGTCTGTGTGGGCACTGGGTTACAGCTCGCGGGGCAAATTAGCGTATTAAG<br>CCGCAGCTATATTGAACATGCCGATATTGTATTTTCACTCTTACCTGACGGTTTCTCGCAGCGT<br>TGGTTGACGAAGCTCAACCCCAATGTCATCAATTTGCAGCAGTTTTATGCGCAAAATGGTGAA<br>GTTAAAAATCGCCGAGACACCTACGAGCAAATGGTCAATGCCATTCTAGATGCGGTGAGAGC<br>GGGTAAAAAAACCGTGTGTGCACTCTACGGTCATCCGGGGGTATTTGCCTGTGTATCCCATAT<br>GGCGATAACTCGGGCGAAGGCCGAAGGGTTTTCGGCAAAGATGGAGCCGGGGATTTCGGCC<br>GAAGCTTGCCTGTGGGCCGACTTAGGGATTGACCCCGGCAACTCGGGGCATCAAAGTTTTGA<br>AGCTAGCCAGTTTATGTTTTTCAACCATGTGCCCGATCCCACTACCCACTTATTACTCTGGCAA<br>ATCGCCATTGCAGGCGAACATACCTTAACCCAATTTCATACCTCGAGTGATAGGTTGCAGATC<br>CTCGTGGAGCAGTTGAATCAATGGTATCCCCTCGACCATGAGGTGGTCATATACGAAGCGGC<br>CAATTTGCCAATCCAAGCCCCGCGTATCGAGCGTTTACCTTTAGCGAATTTACCCCAAGCACA		

CTTAATGCCGATTAGTACGTTGTTAATTCCGCCAGCAAAAAGCTGGAGTACAACTATGCTATT<br>TTGGCTAAGTTAGGGATCGGTCCCGAAGATTTGGGATAA		
Q8EGW2	*sonA* (SO1479)	Borosin RiPP precursor
ATGTCTGGATTATCGGATTTTTTTACCCAGTTAGGCCAAGATGCGCAGTTAATGGAAGACTATA<br>AACAGAATCCTGAGGCGGTGATGCGTGCCCACGGATTAACTGATGAACAAATTAACGCTGTAA<br>TGACTGGGGATATGGAAAAGCTCAAAACGTTAAGTGGTGATAGTAGCTATCAATCTTACCTTGT<br>TATTTCACATGGTAATGGTGATTAA		
n/a	*his₆-sonM*	Hexahistidine tagged borosin precursor for heterologous expression
ATGCATCATCATCATCATCACAGCAGCATGGGATCACTCGTCTGTGTGGGCACTGGGTTACAG<br>CTCGCGGGGCAAATTAGCGTATTAAGCCGCAGCTATATTGAACATGCCGATATTGTATTTTCAC<br>TCTTACCTGACGGTTTCTCGCAGCGTTGGTTGACGAAGCTCAACCCCAATGTCATCAATTTGC<br>AGCAGTTTTATGCGCAAAATGGTGAAGTTAAAAATCGCCGAGACACCTACGAGCAAATGGTCA<br>ATGCCATTCTAGATGCGGTGAGAGCGGGTAAAAAAACCGTGTGTGCACTCTACGGTCATCCG<br>GGGGTATTTGCCTGTGTATCCCATATGGCGATAACTCGGGCGAAGGCCGAAGGGTTTTCGGC<br>AAAGATGGAGCCGGGGATTTCGGCCGAAGCTTGCCTGTGGGCCGACTTAGGGATTGACCCC<br>GGCAACTCGGGGCATCAAAGTTTTGAAGCTAGCCAGTTTATGTTTTTCAACCATGTGCCCGAT<br>CCCACTACCCACTTATTACTCTGGCAAATCGCCATTGCAGGCGAACATACCTTAACCCAATTTC<br>ATACCTCGAGTGATAGGTTGCAGATCCTCGTGGAGCAGTTGAATCAATGGTATCCCCTCGACC<br>ATGAGGTGGTCATATACGAAGCGGCCAATTTGCCAATCCAAGCCCCGCGTATCGAGCGTTTAC<br>CTTTAGCGAATTTACCCCAAGCACACTTAATGCCGATTAGTACGTTGTTAATTCCGCCAGCAAA<br>AAAGCTGGAGTACAACTATGCTATTTTGGCTAAGTTAGGGATCGGTCCCGAAGATTTGGGATA<br>A		
n/a	*his₆-sonA*	Hexahistidine tagged borosin methyltransferase for heterologous expression
ATGCATCATCATCATCATCACATGTCTGGATTATCGGATTTTTTTACCCAGTTAGGCCAAGATG<br>CGCAGTTAATGGAAGACTATAAACAGAATCCTGAGGCGGTGATGCGTGCCCACGGATTAACT<br>GATGAACAAATTAACGCTGTAATGACTGGGGATATGGAAAAGCTCAAAACGTTAAGTGGTGAT<br>AGTAGCTATCAATCTTACCTTGTTATTTCACATGGTAATGGTGATTAA		
n/a	*his₆-sonA-BBD*	Hexahistidine tagged SonA helical bundle/BBD (SonA-BBD)
ATGCATCATCATCATCATCACATGTCTGGATTATCGGATTTTTTTACCCAGTTAGGCCAAGATG<br>CGCAGTTAATGGAAGACTATAAACAGAATCCTGAGGCGGTGATGCGTGCCCACGGATTAACT<br>GATGAACAAATTAACGCTGTAATGACTGGGGATATGGAAAAGCTCAAAACGTTAAGTGGTTAA		
n/a	*sspA$_{NRRLS118}$*	Codon optimized borosin RiPP precursor
ATGCCGGCGGCGGTGGTTGACTTCATGGAGGAACTGGTGACCCAGCCGCGTCGTCAACACG<br>CGTACCGTCGTAGCGCGGAGGCGTATGTTGCGGATAGCGCGCTGACCGCTAGCGAGCGTGA<br>AGCGGTGGTTAGCGGTGACGTGGATCGTATGCGTGCGGTTCTGGCCGAGCACAGCGGCGTG<br>AAAGAGGAGTGCCACGCGGTTCTGGTGGTTATCATTTTTGACCCGGATGAAGTTCCGAGCGG<br>TGCGTAA		
n/a	*sspM$_{NRRLS118}$*	Codon optimized borosin methyltransferase
ATGCAGGAGACCACCGGTAACGCGCAACTGGTGGTTGTGGGTACCGGTTTCCGTGCGATTGG<br>TGACCTGACCGTTGAAGCGCGTGCGTGCCTGGAACAGGCGGACAAGGTTCTGTGCCTGATCG<br>GTGATCCGCTGGTGACCCGTCACATTGAGAAACTGAACGCGAGCGTTGAAACCCTGGATGTT<br>CATTATGCGGTGGGCAAGCCGCGTAGCGCGAGCTATGAGGACATGGTGGAACACATTATGAG<br>CGAACTGCACCGTGATCAATTCGTTTGCGTGGCGCTGTACGGTCACCCGGGCGTTTTTGCGT<br>ATACCGGTCATGAGGCGATCCGTCGTGCGCGTGAGGAAGGCATCGCGGCGCGTATGCTGCC<br>GGCGTGCAGCGCGGAAGACTGGCTGTTTGCGGATCTGGGTCTGGACCCGGGCGAGCGTGGC<br>TGCCAGAGCTTCGAAGCGACCGACTTTCTGATCCGTCACCGTGTGTTTGATCCGACCGGCCT<br>GCTGATTCTGTGGCAAGTTGGTGTGATCGGCATGATTGATCGTGATCCGGGTTATGATGCGCG<br>TCCGGGCGTTACCACCCTGACCGATGCGCTGGTTGCGAGCTACGGTAGCGGCCACCCGGTT<br>ACCGTGTACGAGGCGAGCCCGTATGTTACCGCGGAACCGCGTACCACCACCGTGCCGCTGG<br>CGGAGCTGCCGGACACCCCGCTGAGCGCGGCGAGCACCCTGGTTGTGCCGCCGCTGCCGC<br>CGCGTCCGGTGGATCGTGAACTGCTGGCGCGTCTGGCGGCGCGTCGTTAA		
n/a	*his₆-sspA$_{NRRLS118}$*	Hexahistidine tagged borosin RiPP precursor: SspA$_{NRRLS118}$
ATGGGCAGCAGCCATCATCATCATCATCACAGCAGCGGCCTGGTGCCGCGCGGCAGCCATAT<br>GCCGGCGGCGGTGGTTGACTTCATGGAGGAACTGGTGACCCAGCCGCGTCGTCAACACGCG<br>TACCGTCGTAGCGCGGAGGCGTATGTTGCGGATAGCGCGCTGACCGCTAGCGAGCGTGAAG		

CGGTGGTTAGCGGTGACGTGGATCGTATGCGTGCGGTTCTGGCCGAGCACAGCGGCGTGAA<br>AGAGGAGTGCCACGCGGTTCTGGTGGTTATCATTTTTGACCCGGATGAAGTTCCGAGCGGTG<br>CGTAA		
n/a	*his_6-SUMO-$sspM_{NRRLS118}$*	Hexahistidine and SUMO tagged borosin methyltransferase: $SspM_{NRRLS118}$
ATGGGTAGCCACCACCACCACCATCATCATAGCAGCGGTTTAGTTCCTCGTGGTTCAGCTAGC<br>CACATCAACCTGAAGGTGAAAGGCCAGGATGGCAACGAGGTGTTCTTCCGCATTAAACGCTC<br>AACCCAGCTGAAGAAGCTGATGAACGCGTACTGCGATCGTCAGAGCGTGGATATGACCGCAA<br>TTGCGTTCCTGTTCGATGGTCGTCGTTTACGTGCAGAACAAACCCCGGACGAACTGGAAATGG<br>AAGATGGCGACGAGATTGATGCCATGCTGCATCAGACCGGTGGCCATATGCAGGAGACCACC<br>GGTAACGCGCAACTGGTGGTTGTGGGTACCGGTTTCCGTGCGATTGGTGACCTGACCGTTGA<br>AGCGCGTGCGTGCCTGGAACAGGCGGACAAGGTTCTGTGCCTGATCGGTGATCCGCTGGTG<br>ACCCGTCACATTGAGAAACTGAACGCGAGCGTTGAAACCCTGGATGTTCATTATGCGGTGGG<br>CAAGCCGCGTAGCGCGAGCTATGAGGACATGGTGGAACACATTATGAGCGAACTGCACCGTG<br>ATCAATTCGTTTGCGTGGCGCTGTACGGTCACCCGGGCGTTTTTGCGTATACCGGTCATGAGG<br>CGATCCGTCGTGCGCGTGAGGAAGGCATCGCGGCGCGTATGCTGCCGGCGTGCAGCGCGG<br>AAGACTGGCTGTTTGCGGATCTGGGTCTGGACCCGGGCGAGCGTGGCTGCCAGAGCTTCGA<br>AGCGACCGACTTTCTGATCCGTCACCGTGTGTTTGATCCGACCGGCCTGCTGATTCTGTGGCA<br>AGTTGGTGTGATCGGCATGATTGATCGTGATCCGGGTTATGATGCGCGTCCGGGCGTTACCA<br>CCCTGACCGATGCGCTGGTTGCGAGCTACGGTAGCGGCCACCCGGTTACCGTGTACGAGGC<br>GAGCCCGTATGTTACCGCGGAACCGCGTACCACCACCGTGCCGCTGGCGGAGCTGCCGGAC<br>ACCCCGCTGAGCGCGGCGAGCACCCTGGTTGTGCCGCCGCTGCCGCCGCGTCCGGTGGATC<br>GTGAACTGCTGGCGCGTCTGGCGGCGCGTCGTTAA		

Supplementary Table 4. Parameter values used in our base kinetics model.

Variables	Value (M)
E	0
S	7.57E-05
ES	4.30E-06
EP	7.00E-07
P_1	1.03E-05
P_2	9.00E-06
**Parameters**	**Value (s^{-1})**
k_1	1.21E+03
k_2	8.70E-03
k_3	1.21E+03
k_4	8.70E-02
k_{1r}	1.21E-02
k_{2r}	0
k_{3r}	1.21E-03
k_{4r}	0

Supplementary Table 5. Crystallographic data and statistics.

DATA COLLECTION						
	SonM—SonA-2Me—SAH	SonM—SonA-2Me	SonM-Y58F—SonA-2Me	SonM-Y93F—SonA-2Me	SonM-R67A—SonA-0Me—SAH	SonM—SonA-BBD—(±)SAM
**PDB ID**	7LTE	7LTC	7LTF	7LTH	7LTS	7LTR
**Resolution (Å)**	2.0	2.0	2.2	2.1	2.32	1.75
**Diffraction source**	APS Argonne 23ID-B					
**Wavelength (Å)**	1.033167				0.991840	
**Detector**	EIGER 16M					
**Rotation range per image (˚)**	0.4	0.2	0.4	0.4	0.5	0.5
**Total rotation range (˚)**	180	250	260	300	150	225
**Space group**	$P2_1$	$P2_1$	$P2_1$	$P2_1$	P422	$P2_1$
**Unit-cell parameters (Å)**	a = 52.54, b = 108.75, c = 59.14; α= γ= 90.000, β= 94.0	a = 52.51, b = 108.62, c = 59.02; α= γ= 90.000, β= 94.0	a = 52.37, b = 108.50, c = 58.95; α= γ= 90.000, β= 94.1	a = 52.34, b = 108.71, c = 58.96; α= γ= 90.000, β= 93.9	a = b = 80.95, c = 236.47; α= γ= β= 90.0	a = 72.24, b = 112.86, c = 85.87; α= γ= 90.000, β= 97.8
**Resolution range (Å)**	2.0 (2.1-2.0)	2.0 (2.1-2.0)	2.2 (2.3-2.2)	2.1 (2.2-2.1)	2.32 (2.45-2.32)	1.75 (1.85-1.75)
**N˚ of reflections (last bin)**	134820 (19119)	197428 (27550)	152302 (17833)	205844 (27677)	370988 (53390)	579688 (85609)
**N˚ of unique reflections (last bin)**	42332 (6106)	43871 (6015)	33041 (4031)	38054 (4943)	35110 (5161)	134823 (20453)
**Completeness (%) (last bin)**	94.6 (95.8)	98.4 (99.3)	99.1 (97.8)	99.2 (99.5)	99.9 (99.9)	98.3 (97.4)
**Redundancy**	3.18 (3.27)	4.50(4.58)	4.61 (4.42)	5.41(5.60)	10.56 (10.34)	4.30 (4.19)
**$\langle I/\sigma(I)\rangle$**	14.49 (8.41)	15.53 (8.45)	15.48 (9.42)	17.50 (12.27)	19.16 (2.33)	24.88 (2.56)
**R_{meas} (%)**	6.2 (14.1)	6.1 (17.3)	6.3 (13.0)	5.8 (8.8)	8.9 (172.5)	3.5 (70.0)
**$CC_{1/2}$**	99.7 (99.1)	99.8 (98.8)	99.7 (99.4)	99.8 (99.7)	99.9 (84.6)	99.9 (82.2)
**REFINEMENT STATISTICS**						
**R_{free}/R_{work}**	24.44 / 21.37	23.31 / 20.55	23.76 / 22.55	22.92 / 18.55	23.76 / 19.14	21.05 / 17.96
**N˚ of total model atoms**	6266	5711	5678	6181	5295	10920
**N˚ of water molecules**	1044	543	518	1007	69	993
**Ramachandran core and allowed (%)**	99.6	99.5	99.4	99.3	99.3	99.3
**Ramachandran generously allowed (%)**	0.0	0.2	0.3	0.2	0.0	0.2
**Ramachandran outliers (%)**	0.3	0.3	0.3	0.5	0.7	0.5
Rmsd from ideal						
**Bond lengths (Å)**	0.012	0.006	0.004	0.012	0.002	0.002
**Bond angles (˚)**	1.40	1.26	1.40	1.65	1.16	1.12

Supplementary Figure 1. Phylogenic tree of borosin methyltransferase domains including putative split borosin domains encoded in bacteria. Bayesian posterior probability values support tree branching using the sequence alignment (Supplementary Fig. 2) along with the methyltransferase CobA from *Bacillus megaterium* as the outgroup. The tree was constructed using the MrBayes plugin in Geneious 2019.2 using the following parameters: [Rate Matrix (fixed): wag; Rate Variation: propinv; Gamma Categories: 4; Chain Length: 1,110,000; Subsampling Freq: 1,000; Heated Chains: 4; Burn-in Length: 10,000; Heated Chain Temp: 0.2; Random Seed: 25,028; Unconstrained Branch Lengths: GammaDir (1, 0.1, 1, 1)]. Protein names are listed in general agreement with suggested RiPP nomenclature.[1] Proteins are named in an XxxM/A format that signifies the first letter of the encoding organism's genus followed by two lowercase letters from the species. The terminal letters MA denote a fused methyltransferase and precursor, while the individual letters denote separate methyltransferases (M) or precursors (A). Strain specific identifiers are added when species names are unavailable or when strain-specific genetic differences are present. Previous structurally defined borosin precursors are highlighted in blue; split borosin pathways interrogated in this study are highlighted in green.

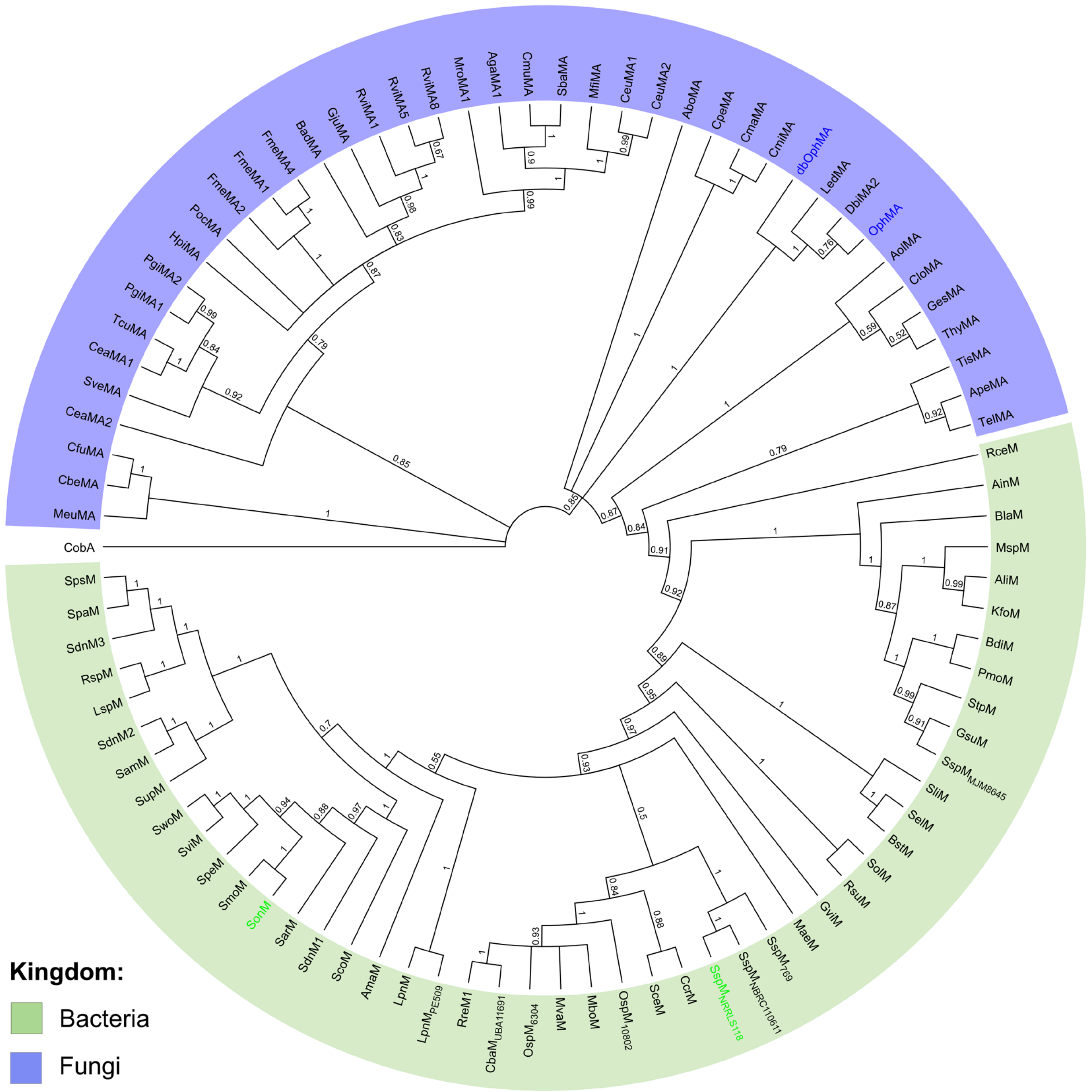

Supplementary Figure 2. Geneious sequence alignment of previously identified fungal borosin and putative bacterial split borosin methyltransferase domains. A cutoff of 90% amino acid identity was used to remove near-duplicate sequences. Methyltransferase domain sequences correspond to Gly10-Ala242 of SonM. Key active site residues are marked with an asterisk (*). Previous structurally defined borosin precursors are highlighted in blue; split borosins interrogated in this study are highlighted in green.

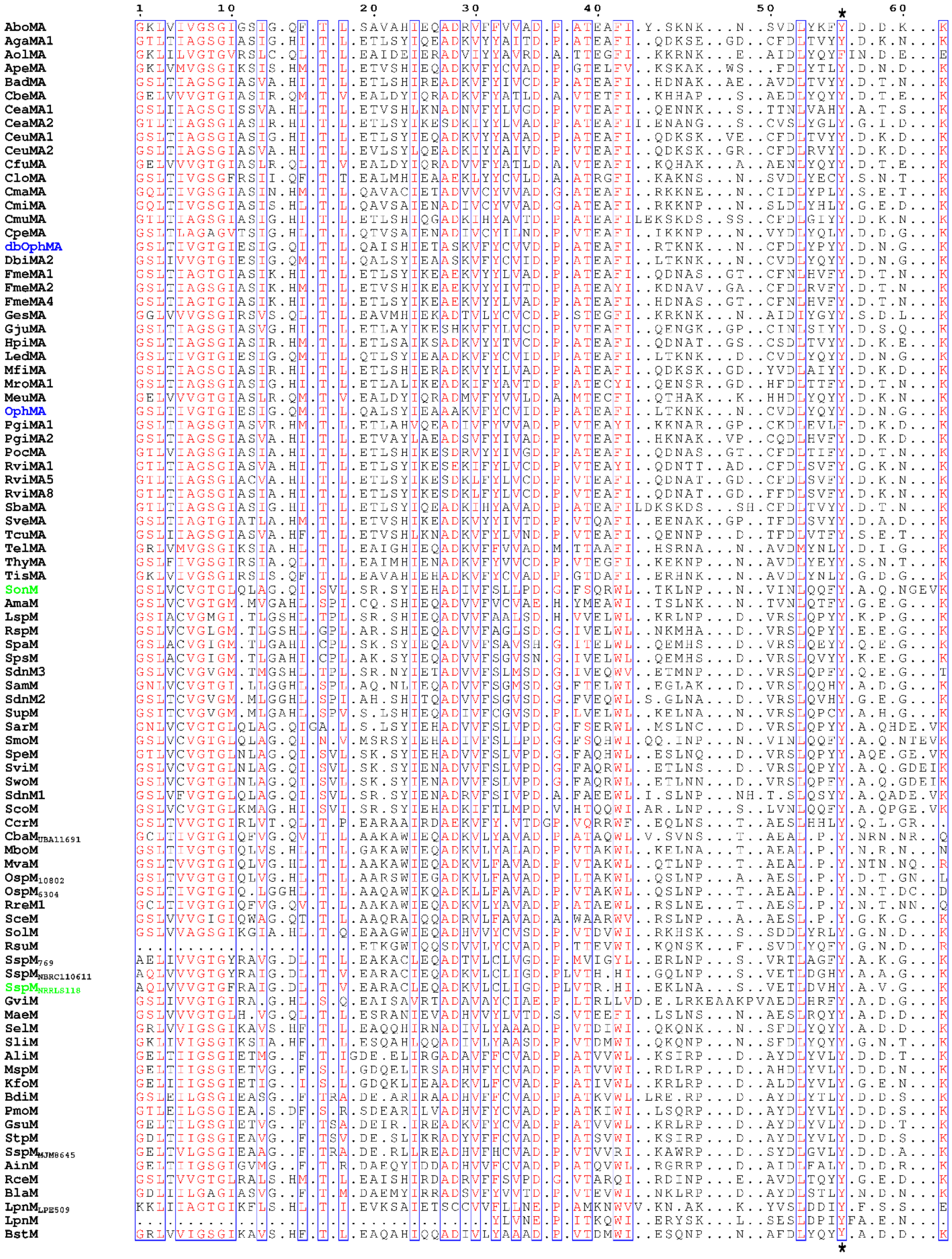

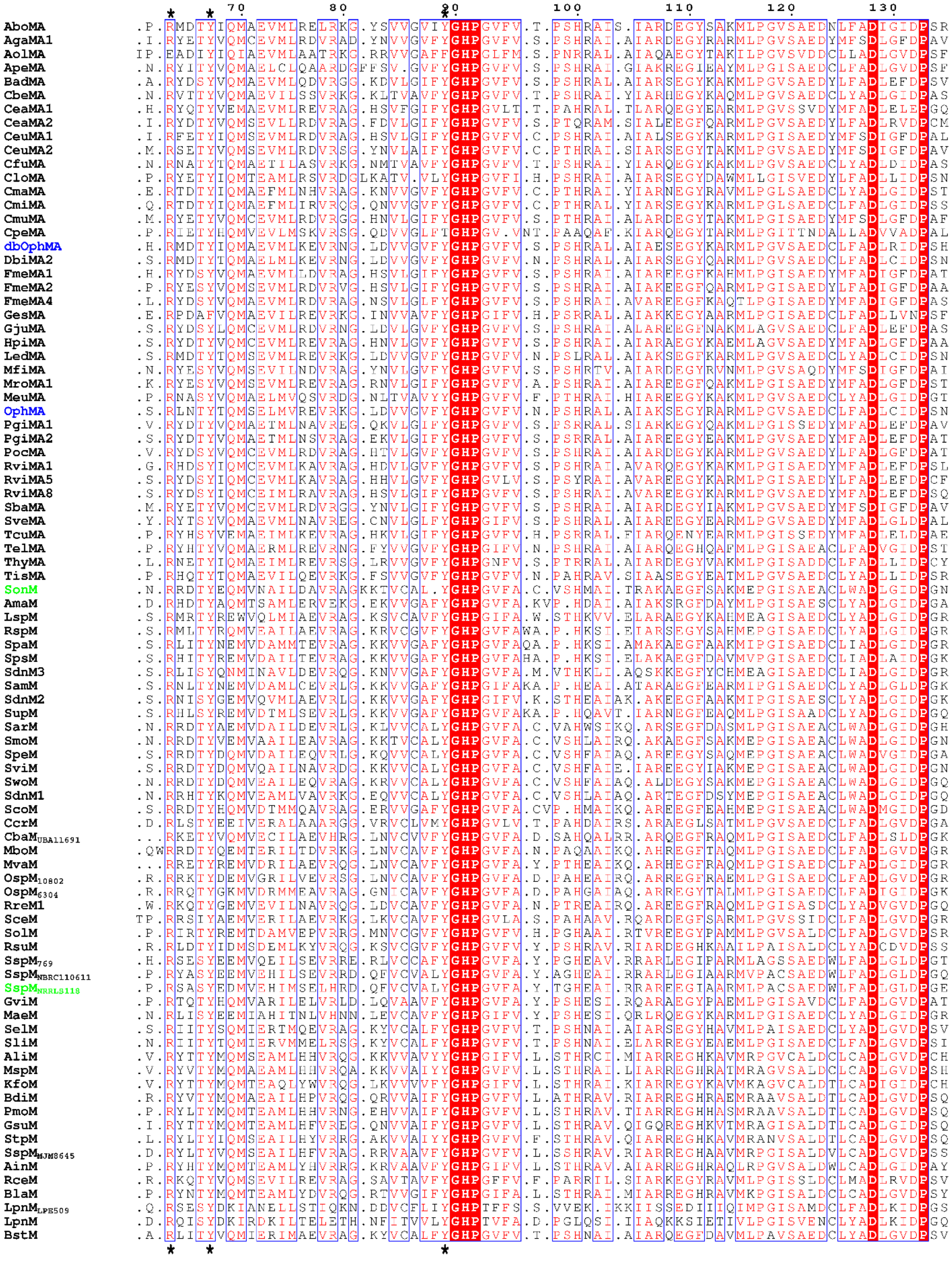
70
80
90
100
110
120
130
AboMA
AgaMA1
AolMA
ApeMA
BadMA
CbeMA
CeaMA1
CeaMA2
CeuMA1
CeuMA2
CfuMA
CloMA
CmaMA
CmiMA
CmuMA
CpeMA
dbOphMA
DbiMA2
FmeMA1
FmeMA2
FmeMA4
GesMA
GjuMA
HpiMA
LedMA
MfiMA
MroMA1
MeuMA
OphMA
PgiMA1
PgiMA2
PocMA
RviMA1
RviMA5
RviMA8
SbaMA
SveMA
TcuMA
TelMA
ThyMA
TisMA
SonM
AmaM
LspM
RspM
SpaM
SpsM
SdnM3
SamM
SdnM2
SupM
SarM
SmoM
SpeM
SviM
SwoM
SdnM1
ScoM
CcrM
CbaM UBA11691
MboM
MvaM
OspM 10802
OspM 6304
RreM1
SceM
SolM
RsuM
SspM 769
SspM NBRC110611
SspM NRRLS118
GviM
MaeM
SelM
SliM
AliM
MspM
KfoM
BdiM
PmoM
GsuM
StpM
SspM MJM8645
AinM
RceM
BlaM
LpnM LPE509
LpnM
BstM

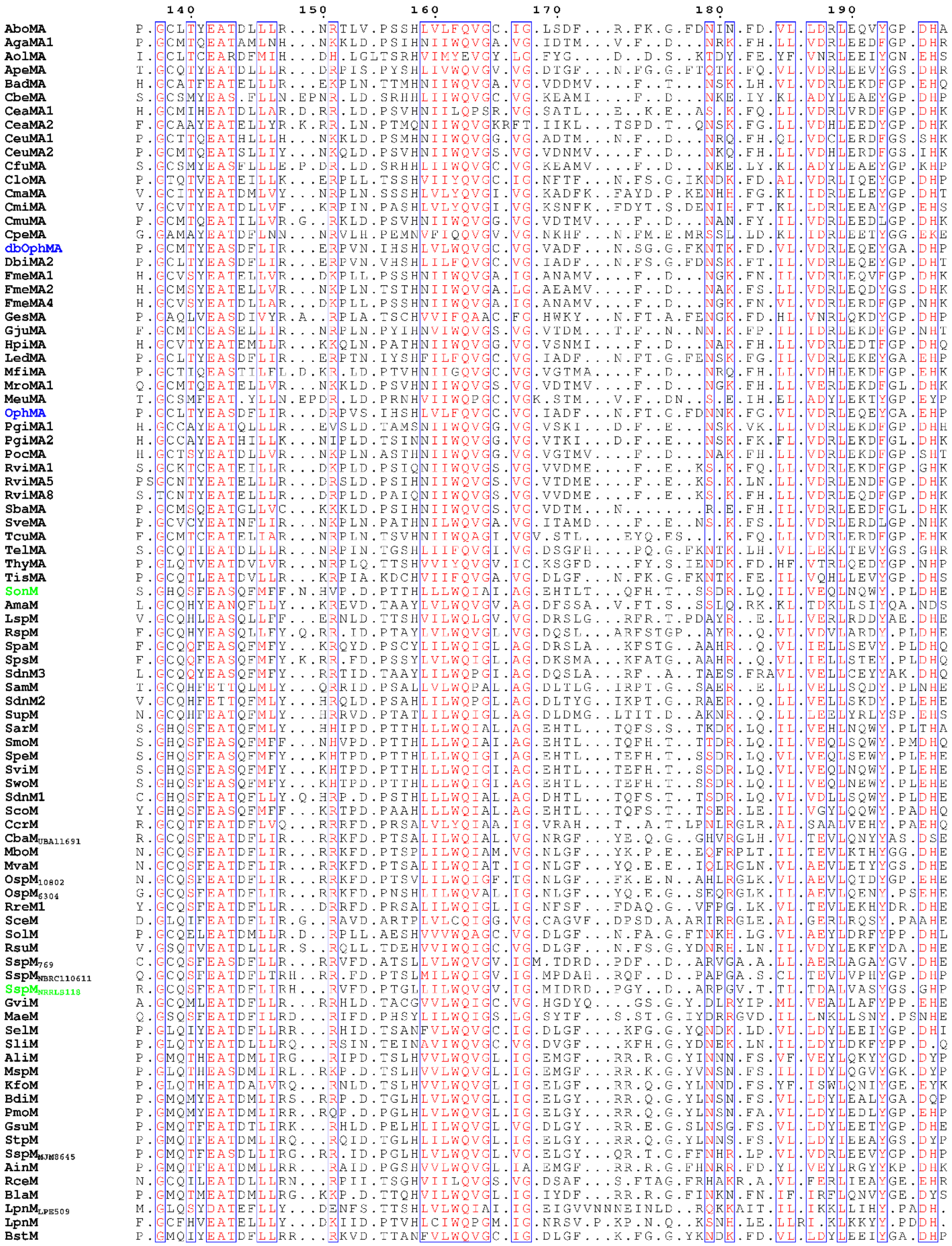
140
150
160
170
180
190
AboMA
AgaMA1
AolMA
ApeMA
BadMA
CbeMA
CeaMA1
CeaMA2
CeuMA1
CeuMA2
CfuMA
CloMA
CmaMA
CmiMA
CmuMA
CpeMA
dbOphMA
DbiMA2
FmeMA1
FmeMA2
FmeMA4
GesMA
GjuMA
HpiMA
LedMA
MfiMA
MroMA1
MeuMA
OphMA
PgiMA1
PgiMA2
PocMA
RviMA1
RviMA5
RviMA8
SbaMA
SveMA
TcuMA
TelMA
ThyMA
TisMA
SonM
AmaM
LspM
RspM
SpaM
SpsM
SdnM3
SamM
SdnM2
SupM
SarM
SmoM
SpeM
SviM
SwoM
SdnM1
ScoM
CcrM
CbaM$_{UBA11691}$
MboM
MvaM
OspM$_{10802}$
OspM$_{6304}$
RreM1
SceM
SolM
RsuM
SspM$_{769}$
SspM$_{NBRC110611}$
SspM$_{NRRLS118}$
GviM
MaeM
SelM
SliM
AliM
MspM
KfoM
BdiM
PmoM
GsuM
StpM
SspM$_{MJM8645}$
AinM
RceM
BlaM
LpnM$_{LPE509}$
LpnM
BstM

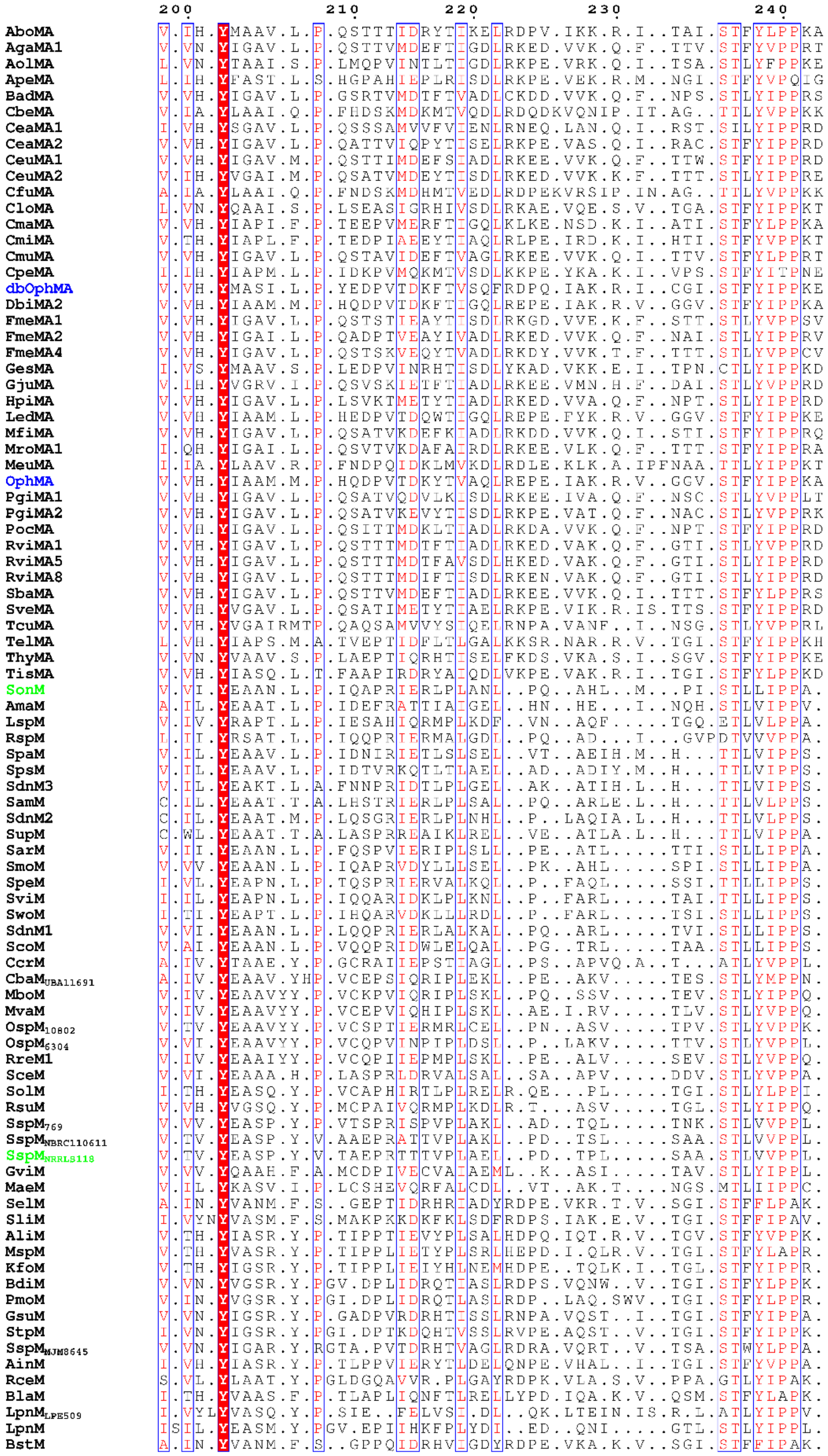
200
210
220
230
240
AboMA
AgaMA1
AolMA
ApeMA
BadMA
CbeMA
CeaMA1
CeaMA2
CeuMA1
CeuMA2
CfuMA
CloMA
CmaMA
CmiMA
CmuMA
CpeMA
dbOphMA
DbiMA2
FmeMA1
FmeMA2
FmeMA4
GesMA
GjuMA
HpiMA
LedMA
MfiMA
MroMA1
MeuMA
OphMA
PgiMA1
PgiMA2
PocMA
RviMA1
RviMA5
RviMA8
SbaMA
SveMA
TcuMA
TelMA
ThyMA
TisMA
SonM
AmaM
LspM
RspM
SpaM
SpsM
SdnM3
SamM
SdnM2
SupM
SarM
SmoM
SpeM
SviM
SwoM
SdnM1
ScoM
CcrM
CbaMUBA11691
MboM
MvaM
OspM10802
OspM6304
RreM1
SceM
SolM
RsuM
SspM769
SspMNBRC110611
SspMNRRLS118
GviM
MaeM
SelM
SliM
AliM
MspM
KfoM
BdiM
PmoM
GsuM
StpM
SspMMJM8645
AinM
RceM
BlaM
LpnMLPE509
LpnM
BstM

Supplementary Figure 3. Gene clusters of the two split borosins analyzed in this study. The putative split borosin gene clusters of (**a**) *Shewanella oneidensis* MR-1 and (**b**) *Streptomyces* sp. NRRL S-118 are depicted with genes as arrows. Protein IDs are given along with the gene name or proposed enzyme function. Putative split borosin precursor genes are colored in cyan, while α-*N*-methyltransferases are colored green.

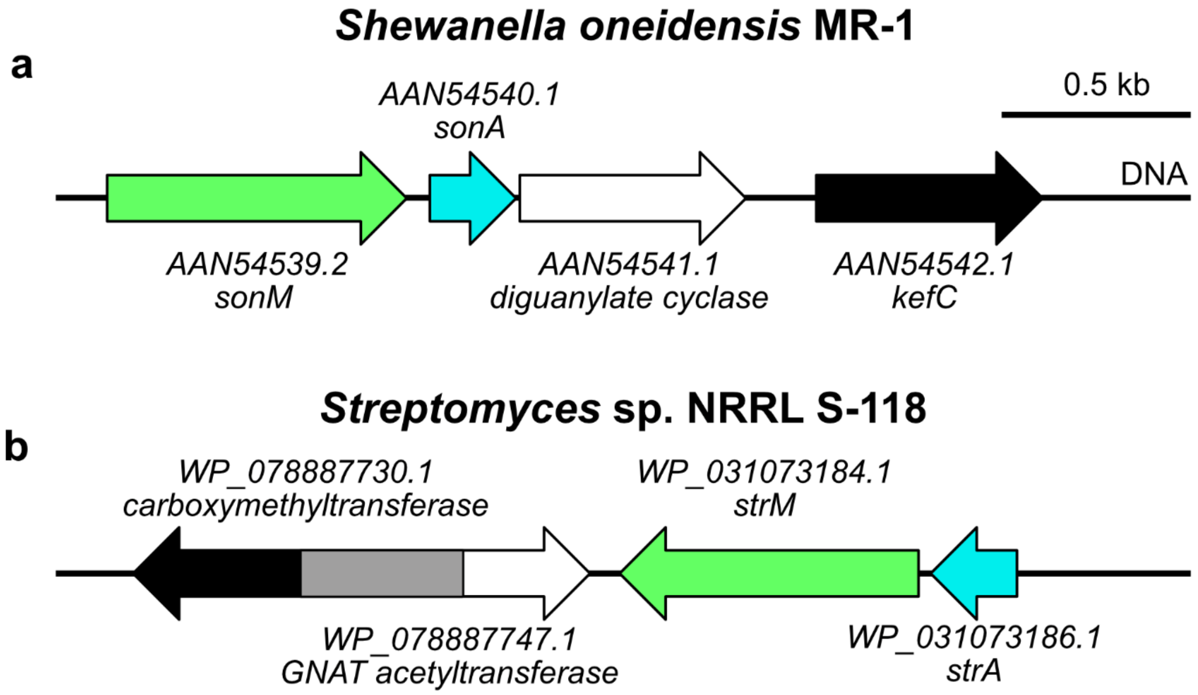

Supplementary Figure 4. Mass spectrometric analysis of split borosin coexpressions. (**a-c**) HPLC-MS/MS spectra from AspN-digested SonA peptides after incubation at 30°C with wt SonM, saturating [SAM], and the other enzymes and kit reagents used in the kinetics assay (see materials and methods). The amino acid sequence above each spectra depicts the *N*-methylated residues that could be confirmed by MS/MS fragmentation (solid orange circles) or are inferred *N*-methylated since the position is not completely defined by MS/MS (unfilled orange circles). Lowercase 'c' denotes cysteine derivatized by iodoacetimide. Observed MS/MS fragmentation masses are listed above (b-ions) and below (y-ions) the amino acid sequence. The gray lines within the sequence mark the sites of fragmentation. Masses of methylation-containing ions are denoted in brackets, where 'Me' stands for methylation. The ppm difference from the observed masses to the theoretical expected masses are labeled in parentheses. A 10.0-ppm mass cutoff for annotated HPLC-MS/MS peaks was used. The protein, time of *in vitro* reaction, parent ion information and HPLC retention time (RT) are listed in the upper right corner of the LC-MS/MS spectra. Note, panel c depicts the same raw data as seen in Fig. 1d. (**d-i**) HPLC-MS/MS spectra from GluC-digested SspA$_{NRRLS118}$ peptides after a 15 min incubation at room temperature with SspM$_{NRRLS118}$, saturating [SAM], and *S*-adenosylhomocysteine nucleosidase in 50 mM HEPES buffer. (**j**) Relative abundance of SspA$_{NRRLS118}$ peptides from panels (d-h). The methylation state is indicated over each graph (0-4) in an orange box with the relative abundance (%) of the methylated species directly below. Relative abundance (intensity %) was determined by integrating under each peak from the MS1 extracted ion chromatogram. Each peak is plotted over its retention time (x-axis).

a

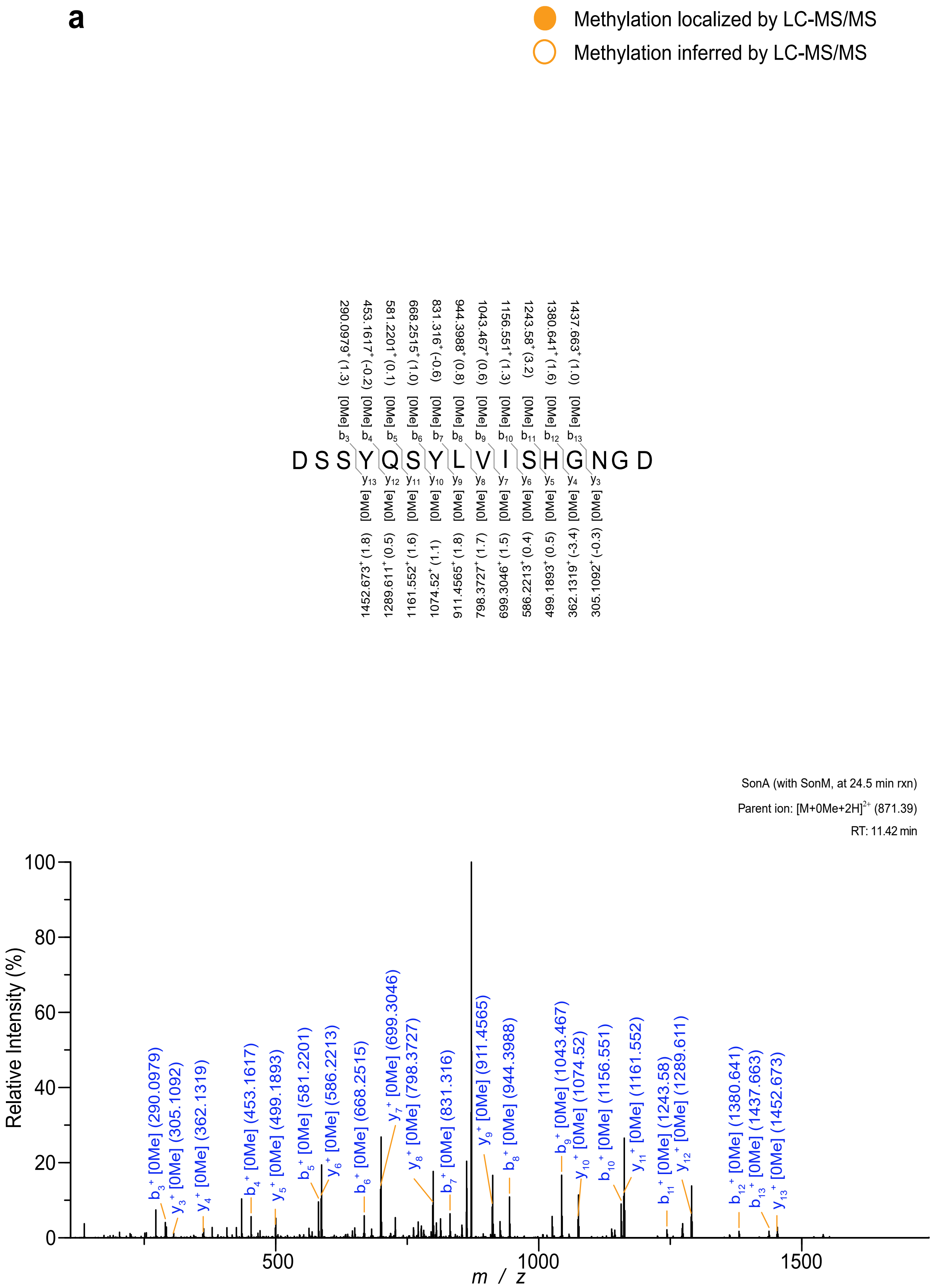
Methylation localized by LC-MS/MS
Methylation inferred by LC-MS/MS
D S S Y Q S Y L V I S H G N G D
SonA (with SonM, at 24.5 min rxn)
Parent ion: [M+0Me+2H]2+ (871.39)
RT: 11.42 min
Relative Intensity (%)
m / z

b

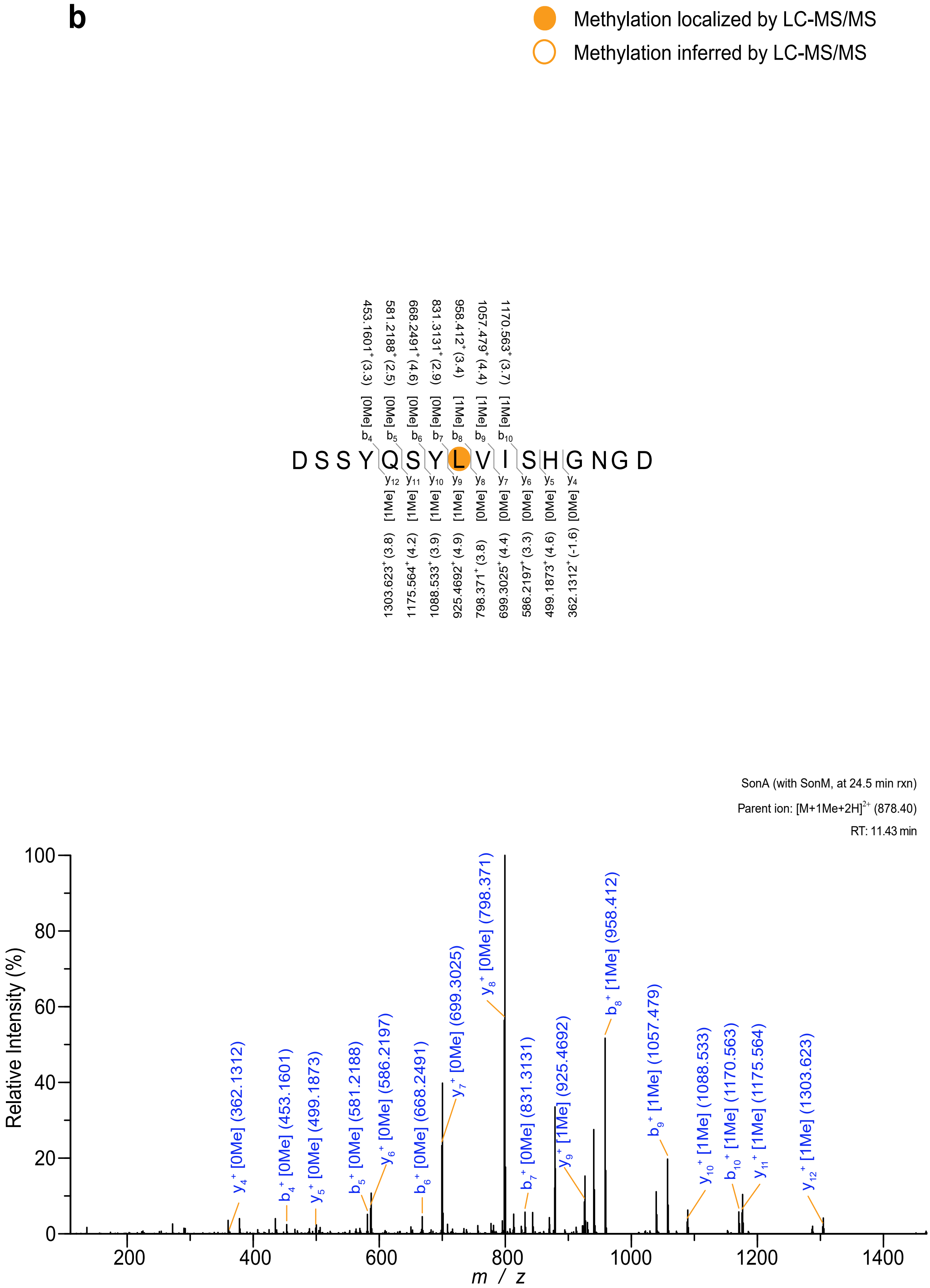

Methylation localized by LC-MS/MS
Methylation inferred by LC-MS/MS
D S S Y Q S Y L V I S H G N G D
453.1601+ (3.3) [0Me] b4
581.2188+ (2.5) [0Me] b5
668.2491+ (4.6) [0Me] b6
831.3131+ (2.9) [0Me] b7
958.412+ (3.4) [1Me] b8
1057.479+ (4.4) [1Me] b9
1170.563+ (3.7) [1Me] b10
y12 [1Me] 1303.623+ (3.8)
y11 [1Me] 1175.564+ (4.2)
y10 [1Me] 1088.533+ (3.9)
y9 [1Me] 925.4692+ (4.9)
y8 [0Me] 798.371+ (3.8)
y7 [0Me] 699.3025+ (4.4)
y6 [0Me] 586.2197+ (3.3)
y5 [0Me] 499.1873+ (4.6)
y4 [0Me] 362.1312+ (-1.6)
SonA (with SonM, at 24.5 min rxn)
Parent ion: [M+1Me+2H]2+ (878.40)
RT: 11.43 min
Relative Intensity (%)
m / z
y4+ [0Me] (362.1312)
b4+ [0Me] (453.1601)
y5+ [0Me] (499.1873)
b5+ [0Me] (581.2188)
y6+ [0Me] (586.2197)
b6+ [0Me] (668.2491)
y7+ [0Me] (699.3025)
y8+ [0Me] (798.371)
b7+ [0Me] (831.3131)
y9+ [1Me] (925.4692)
b8+ [1Me] (958.412)
b9+ [1Me] (1057.479)
y10+ [1Me] (1088.533)
b10+ [1Me] (1170.563)
y11+ [1Me] (1175.564)
y12+ [1Me] (1303.623)

c

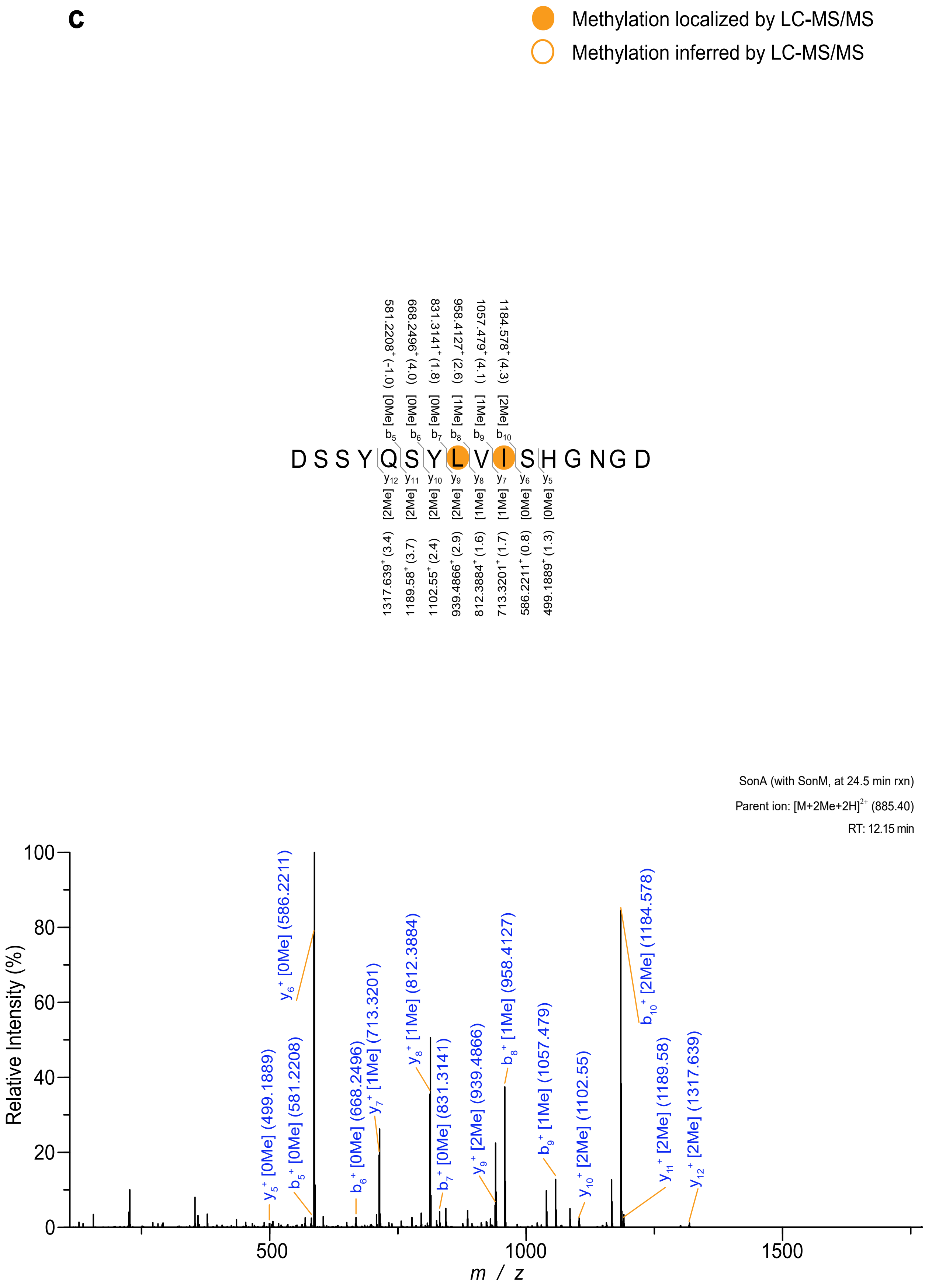

Methylation localized by LC-MS/MS
Methylation inferred by LC-MS/MS
581.2208+ (-1.0) [0Me] b5
668.2496+ (4.0) [0Me] b6
831.3141+ (1.8) [0Me] b7
958.4127+ (2.6) [1Me] b8
1057.479+ (4.1) [1Me] b9
1184.578+ (4.3) [2Me] b10
D S S Y Q S Y L V I S H G N G D
y12 [2Me] 1317.639+ (3.4)
y11 [2Me] 1189.58+ (3.7)
y10 [2Me] 1102.55+ (2.4)
y9 [2Me] 939.4866+ (2.9)
y8 [1Me] 812.3884+ (1.6)
y7 [1Me] 713.3201+ (1.7)
y6 [0Me] 586.2211+ (0.8)
y5 [0Me] 499.1889+ (1.3)
SonA (with SonM, at 24.5 min rxn)
Parent ion: [M+2Me+2H]2+ (885.40)
RT: 12.15 min
Relative Intensity (%)
0
20
40
60
80
100
500
1000
1500
m / z
y5+ [0Me] (499.1889)
b5+ [0Me] (581.2208)
y6+ [0Me] (586.2211)
b6+ [0Me] (668.2496)
y7+ [1Me] (713.3201)
y8+ [1Me] (812.3884)
b7+ [0Me] (831.3141)
y9+ [2Me] (939.4866)
b8+ [1Me] (958.4127)
b9+ [1Me] (1057.479)
y10+ [2Me] (1102.55)
b10+ [2Me] (1184.578)
y11+ [2Me] (1189.58)
y12+ [2Me] (1317.639)

d

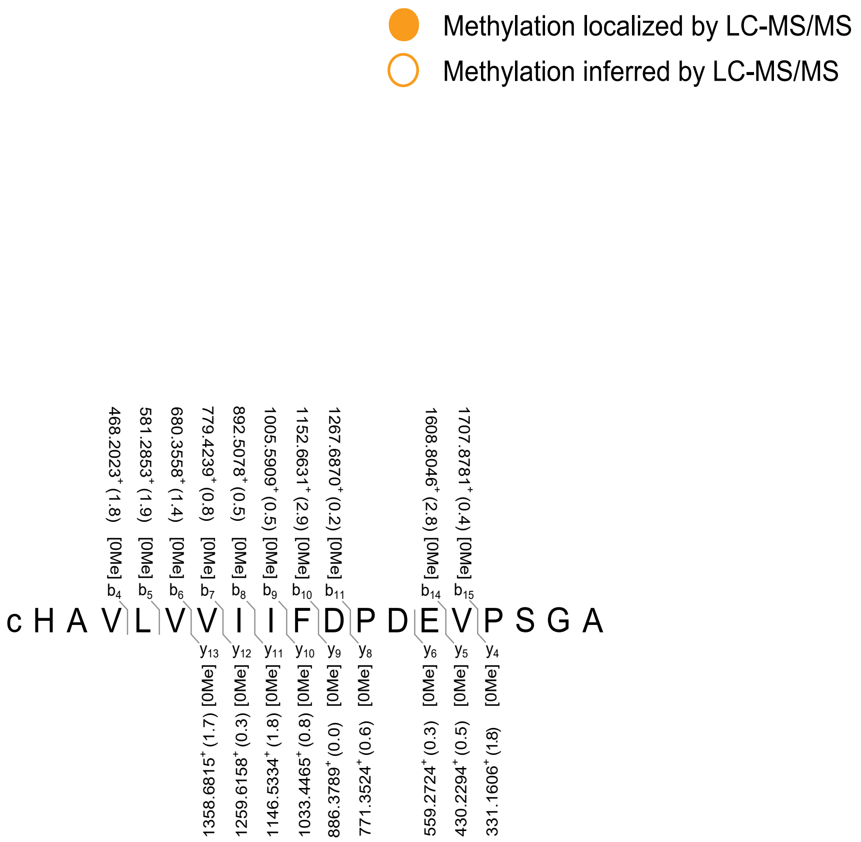
Methylation localized by LC-MS/MS
Methylation inferred by LC-MS/MS
c H A V L V V I I F D P D E V P S G A

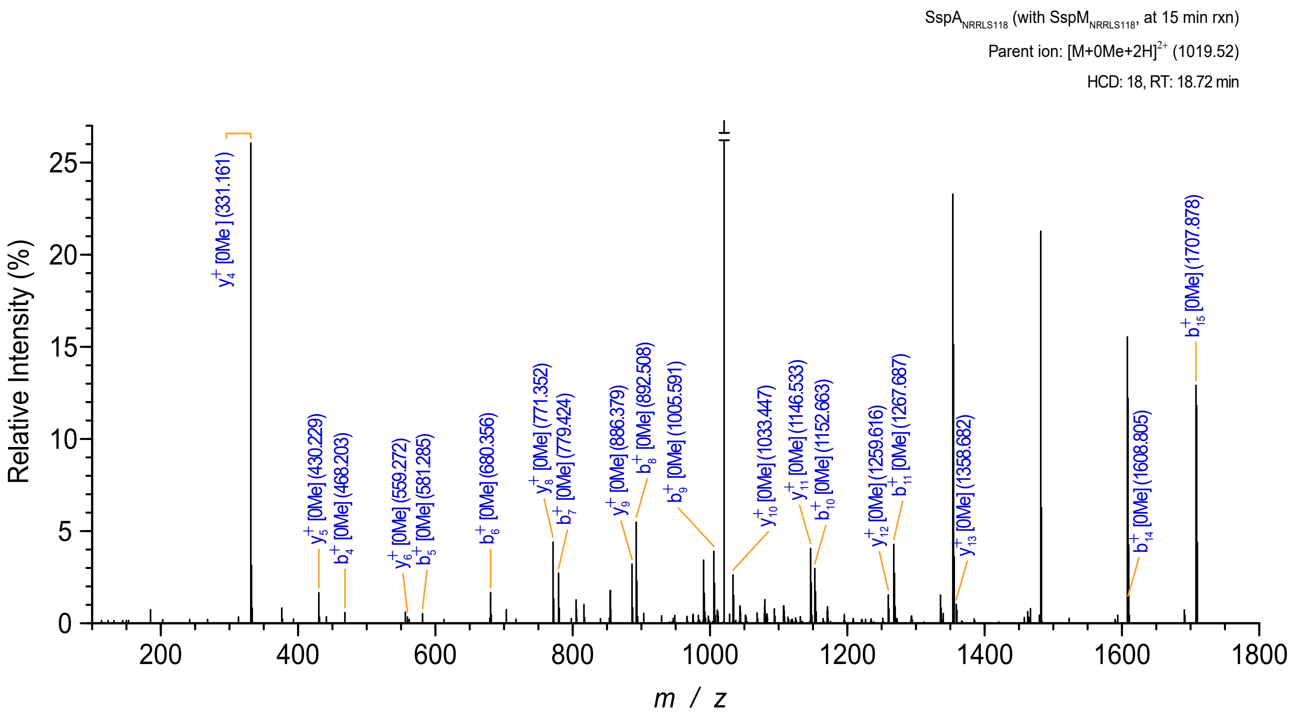
SspA NRRLS118 (with SspM NRRLS118, at 15 min rxn)
Parent ion: [M+0Me+2H]2+ (1019.52)
HCD: 18, RT: 18.72 min
Relative Intensity (%)
m / z

e

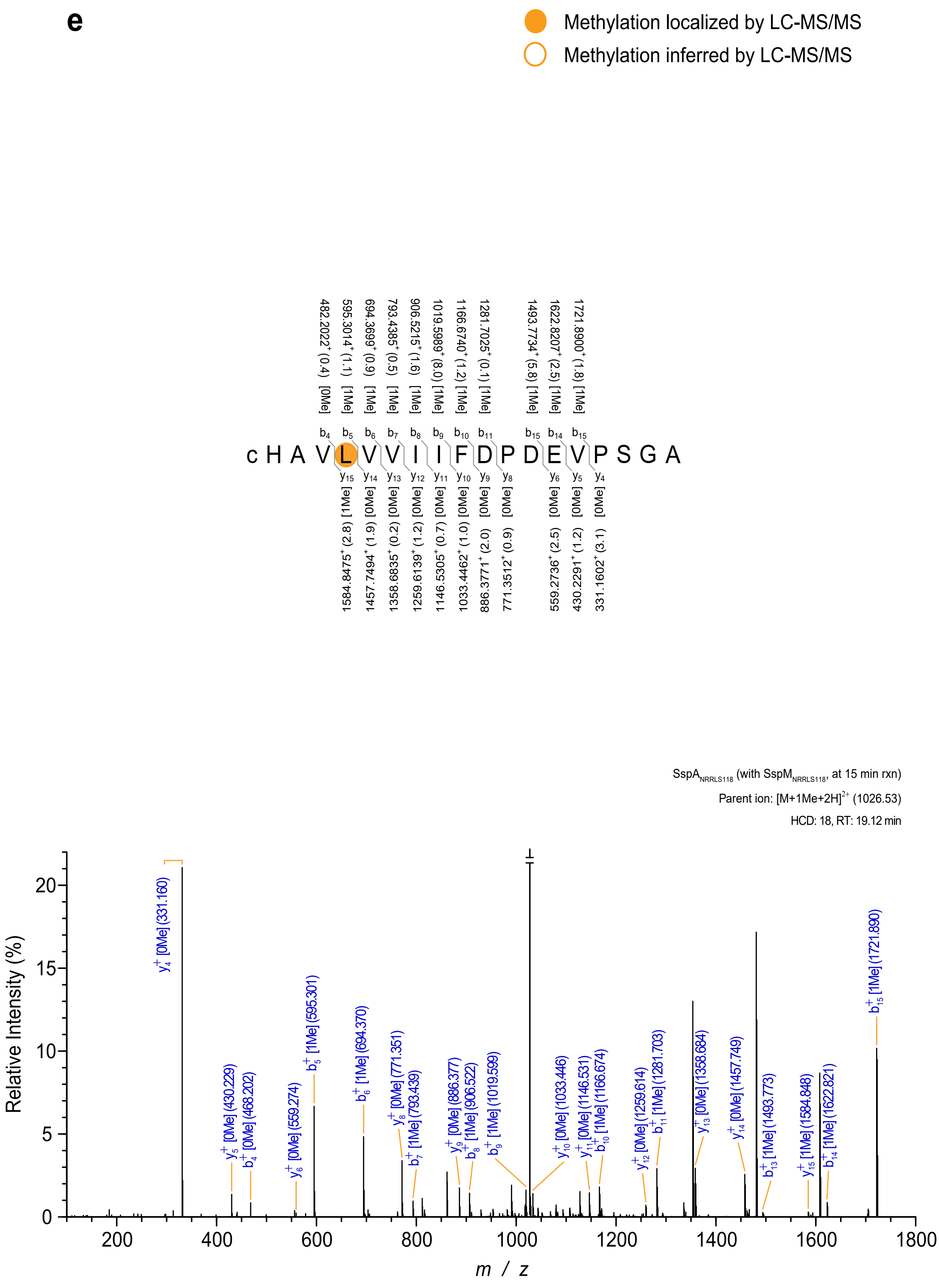

Methylation localized by LC-MS/MS
Methylation inferred by LC-MS/MS
c H A V L V V I I F D P D E V P S G A
482.2022+ (0.4) [0Me]
595.3014+ (1.1) [1Me]
694.3699+ (0.9) [1Me]
793.4385+ (0.5) [1Me]
906.5215+ (1.6) [1Me]
1019.5989+ (8.0) [1Me]
1166.6740+ (1.2) [1Me]
1281.7025+ (0.1) [1Me]
1493.7734+ (5.8) [1Me]
1622.8207+ (2.5) [1Me]
1721.8900+ (1.8) [1Me]
1584.8475+ (2.8) [1Me]
1457.7494+ (1.9) [0Me]
1358.6835+ (0.2) [0Me]
1259.6139+ (1.2) [0Me]
1146.5305+ (0.7) [0Me]
1033.4462+ (1.0) [0Me]
886.3771+ (2.0) [0Me]
771.3512+ (0.9) [0Me]
559.2736+ (2.5) [0Me]
430.2291+ (1.2) [0Me]
331.1602+ (3.1) [0Me]
SspA NRRLS118 (with SspM NRRLS118, at 15 min rxn)
Parent ion: [M+1Me+2H]2+ (1026.53)
HCD: 18, RT: 19.12 min
Relative Intensity (%)
m / z

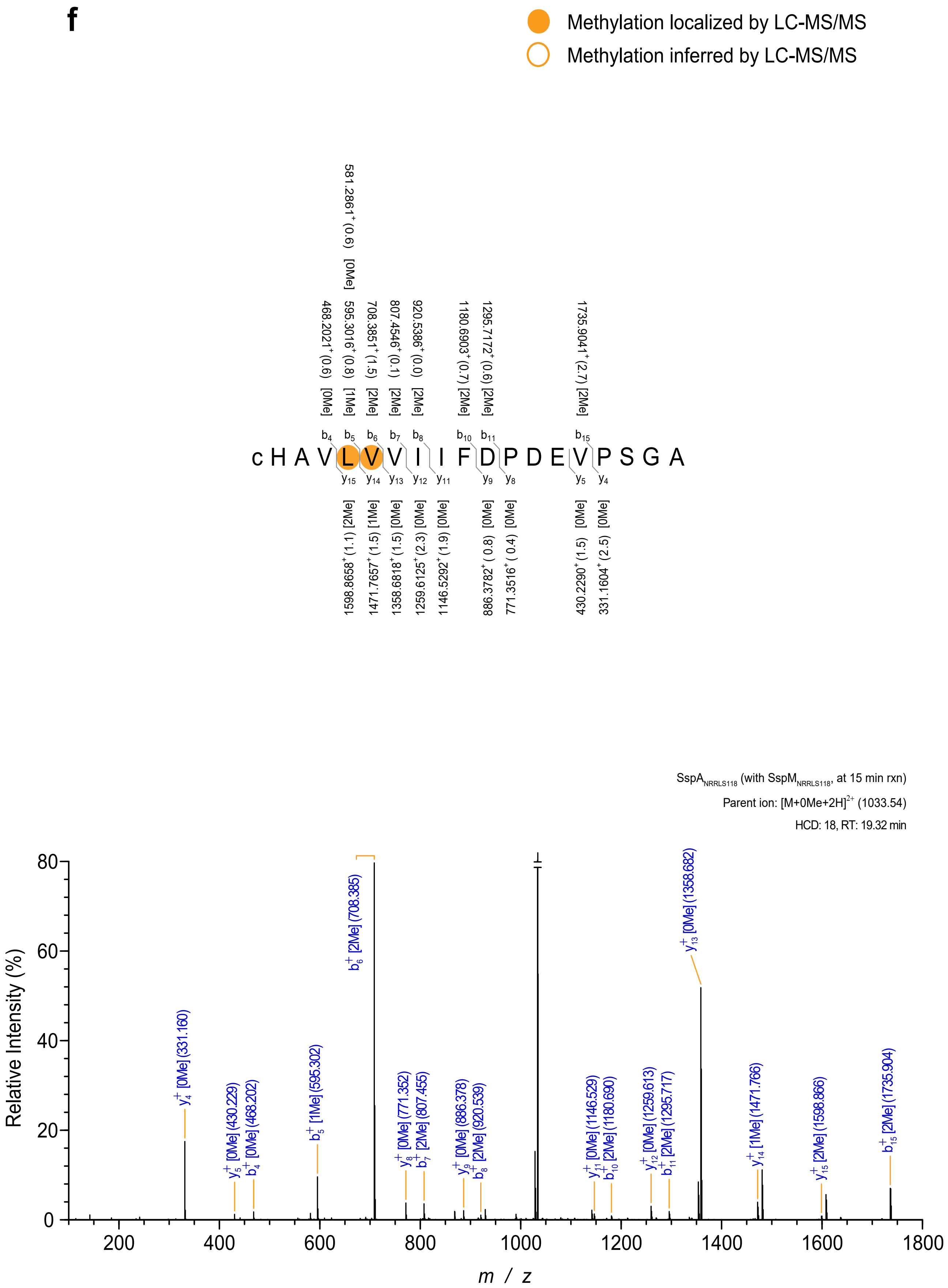
f
Methylation localized by LC-MS/MS
Methylation inferred by LC-MS/MS
c H A V L V V I I F D P D E V P S G A
SspA NRRLS118 (with SspM NRRLS118, at 15 min rxn)
Parent ion: [M+0Me+2H]2+ (1033.54)
HCD: 18, RT: 19.32 min
Relative Intensity (%)
m / z

g

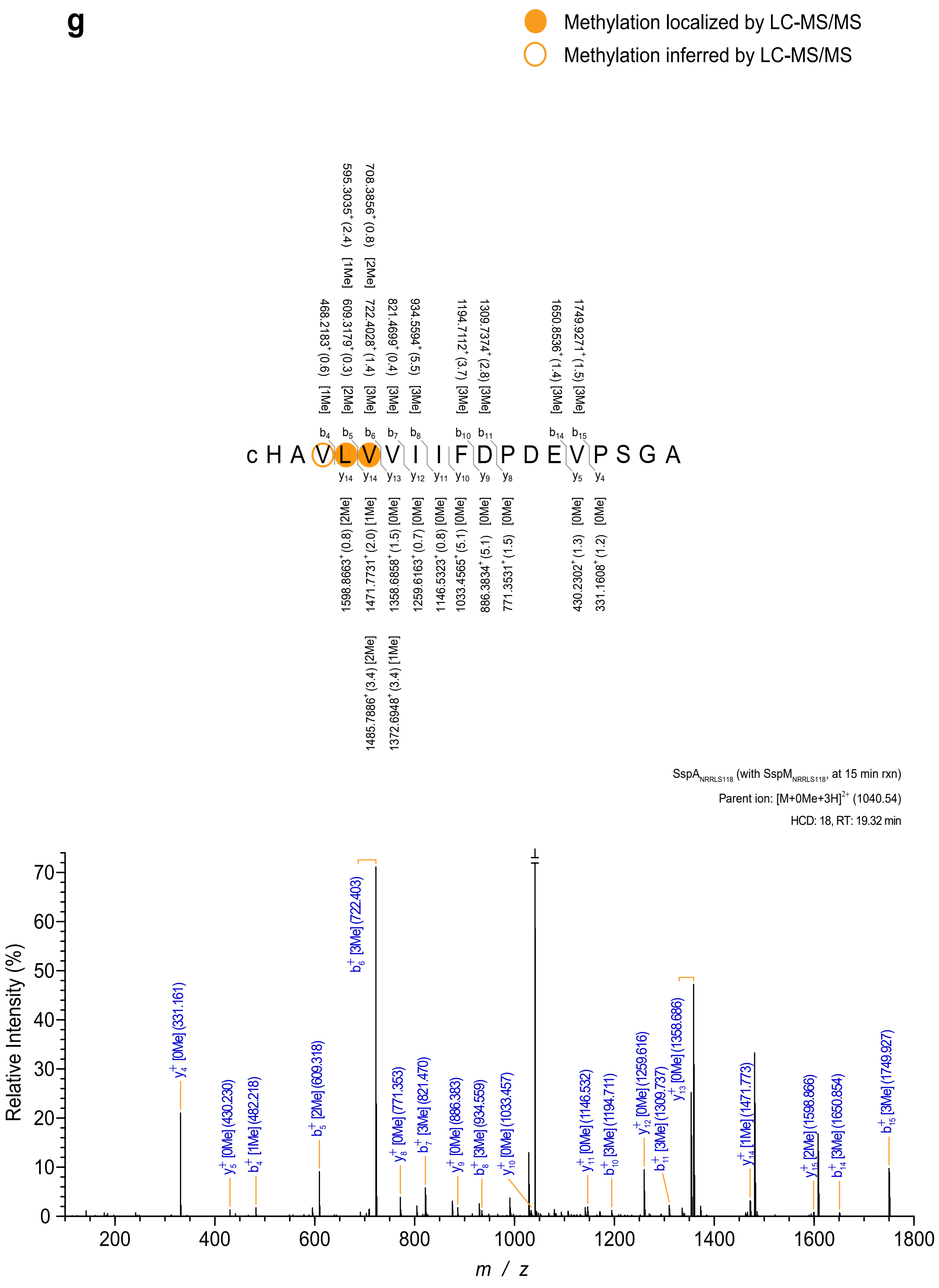

Methylation localized by LC-MS/MS
Methylation inferred by LC-MS/MS
c H A V L V V I I F D P D E V P S G A
SspA$_{NRRLS118}$ (with SspM$_{NRRLS118}$, at 15 min rxn)
Parent ion: [M+0Me+3H]$^{2+}$ (1040.54)
HCD: 18, RT: 19.32 min
Relative Intensity (%)
m / z

h

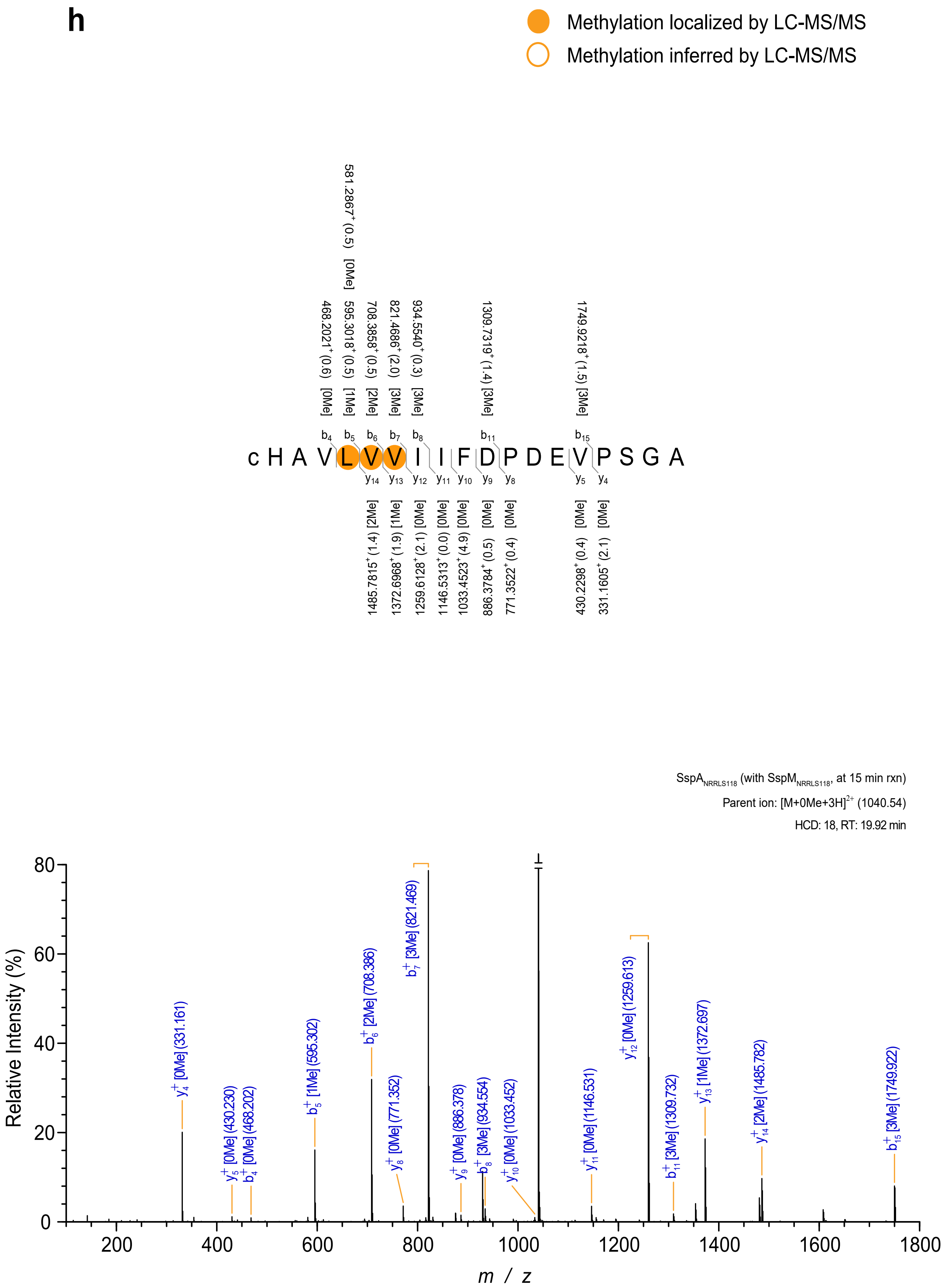
Methylation localized by LC-MS/MS
Methylation inferred by LC-MS/MS
c H A V L V V I I F D P D E V P S G A
SspA_NRRLS118 (with SspM_NRRLS118, at 15 min rxn)
Parent ion: [M+0Me+3H]2+ (1040.54)
HCD: 18, RT: 19.92 min
Relative Intensity (%)
m / z

i

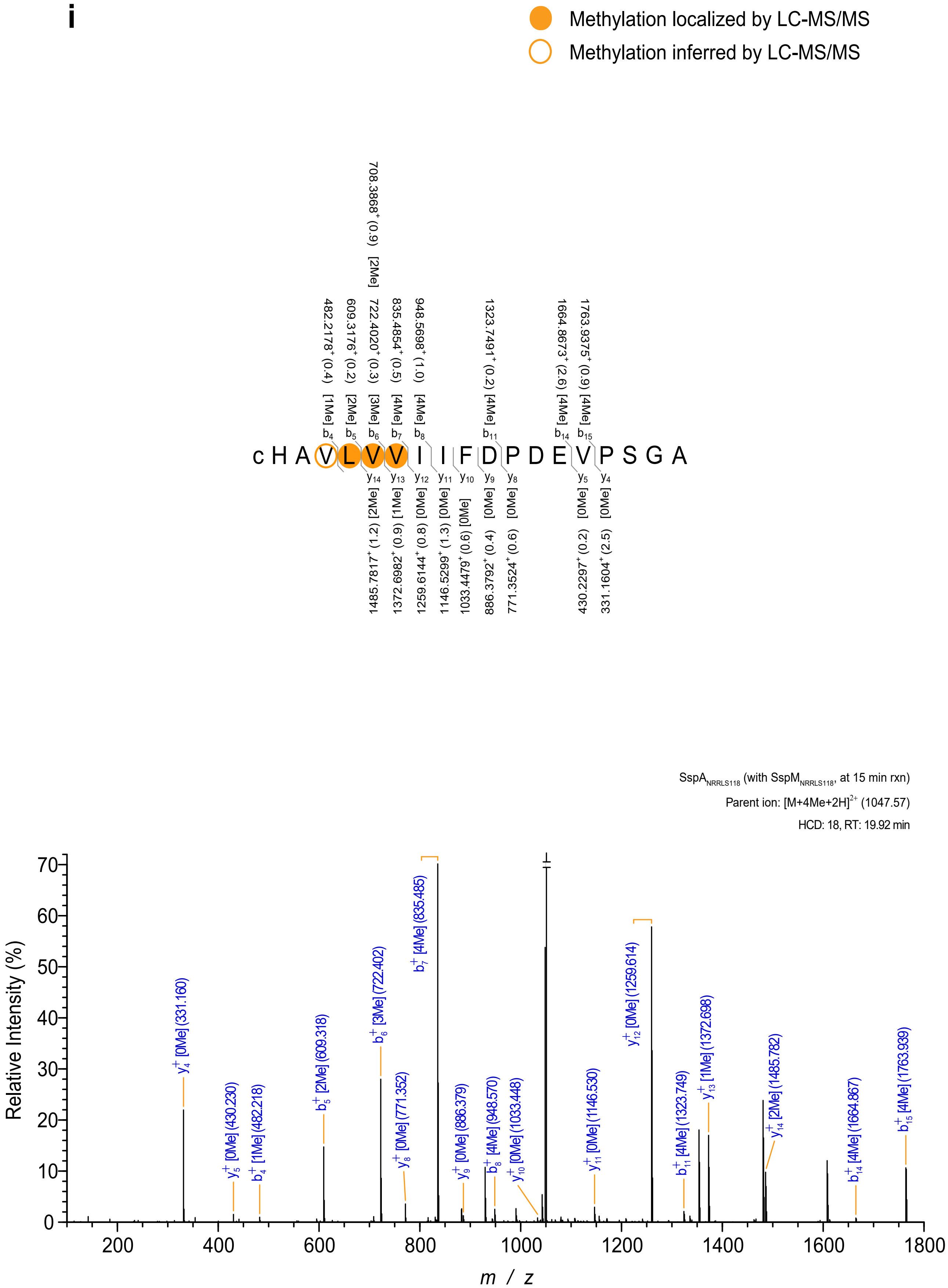
Methylation localized by LC-MS/MS
Methylation inferred by LC-MS/MS
c H A V L V V I I F D P D E V P S G A
SspA NRRLS118 (with SspM NRRLS118, at 15 min rxn)
Parent ion: [M+4Me+2H]2+ (1047.57)
HCD: 18, RT: 19.92 min
Relative Intensity (%)
m / z

j EIC from HPLC-MS: $SspA_{NRRLS118}$ (GluC digest)

15 min

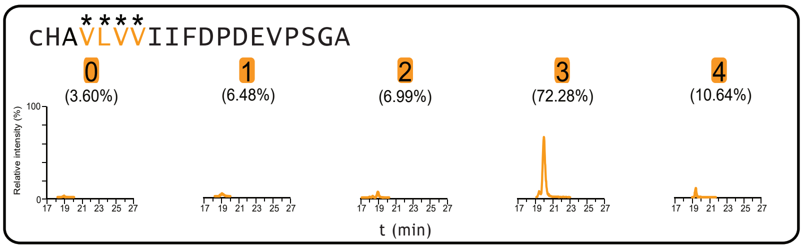

Supplementary Figure 5. Global structural comparisons of borosin α-*N*-methylating enzymes. The (**a**) SonM—SonA-2Me—SAH (PDB: 7LTE) heteromeric configuration is different from the fused homologous fungal systems (**b**) OphMA[2] (PDB: 5N0X) and (**c**) dbOphMA[3] (PDB: 6MJG). A single heterodimer (SonM—SonA-2Me—SAH) or a single monomer of a homodimer (OphMA and dbOphMA) is represented as a cartoon, with SAH depicted as orange sticks.

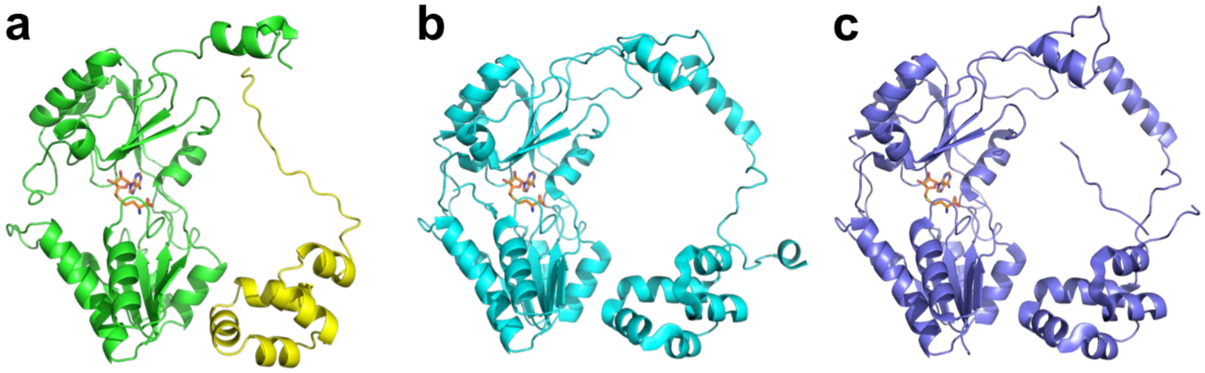

Supplementary Figure 6. Oligomeric state of his$_6$-SonM and his$_6$-SonA proteins by size exclusion chromatography. Size exclusion chromatogram of (**a**) his$_6$-SonM and (**b**) his$_6$-SonA after 24 hour expression in *E. coli* BL21(DE3) cells and nickel affinity purification. The volume at which the protein eluted is indicated with a purple arrow on the x-axis and labelled above the peak. (**c**) The calibration curve used to determine the oligomeric state of his$_6$-SonM and his$_6$-SonA in solution. The x-axis is the molecular weight in log scale, and the y-axis is the partition coefficient ($K_{av} = (V_E - V_0)/(V_C - V_0)$). The molecular weight markers used were: aprotinin (6.5 kDa), ribonuclease A (13.7 kDa), carbonic anhydrase (29 kDa), ovalbumin (43 kDa), and conalbumin (75 kDa). All proteins and standards were run on a HiLoad 16/600 Superdex 75 pg column (Cytiva). The observed mass of the his$_6$-SonM dimer was 48.5 kDa compared to the theoretical mass of 60.3 kDa. The extensive dimer interface of his$_6$-SonM likely accounts for a smaller hydrodynamic radius and the slightly delayed elution time observed. The observed mass of the his$_6$-SonA monomer is 7.5 kDa compared to the theoretical mass of 8.7 kDa. These results have been repeated at least twice from separate expressions with each protein. (**d**) SDS-PAGE of purified his$_6$-SonM (lane 1) and his$_6$-SonA (lane 2) proteins. A standard 15% (w/v) SDS-gel was loaded with 10 µg of each respective protein from the pooled elution fractions from size exclusion chromatography. Source data are provided as a Source Data file.

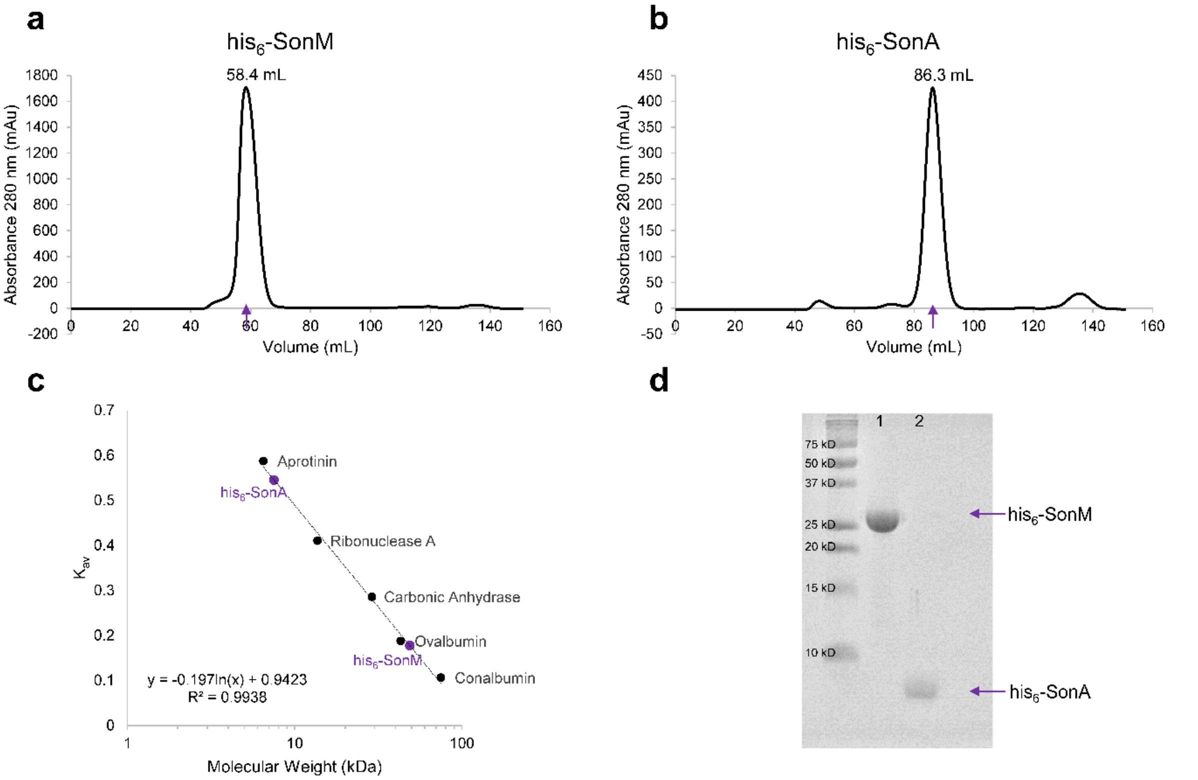

Supplementary Figure 7. Structural overlay of SonM—SonA-2Me—SAH. The SonM—SonA-2Me—SAH complex (SonA is shown in yellow and cyan cartoon, SonM in purple and green, PDB: 7LTE) shows structural similarity to homologous systems (**a**) OphMA[2] (PDB: 5N0X) and (**b**) dbOphMA[3] (PDB: 6MJG). Significant translation movement is visible for SonA (yellow) compared to counterparts in (**c**) OphMA and (**d**) dbOphMA. Key distances are depicted as black dashed lines and their lengths noted in italics in Ångstroms.

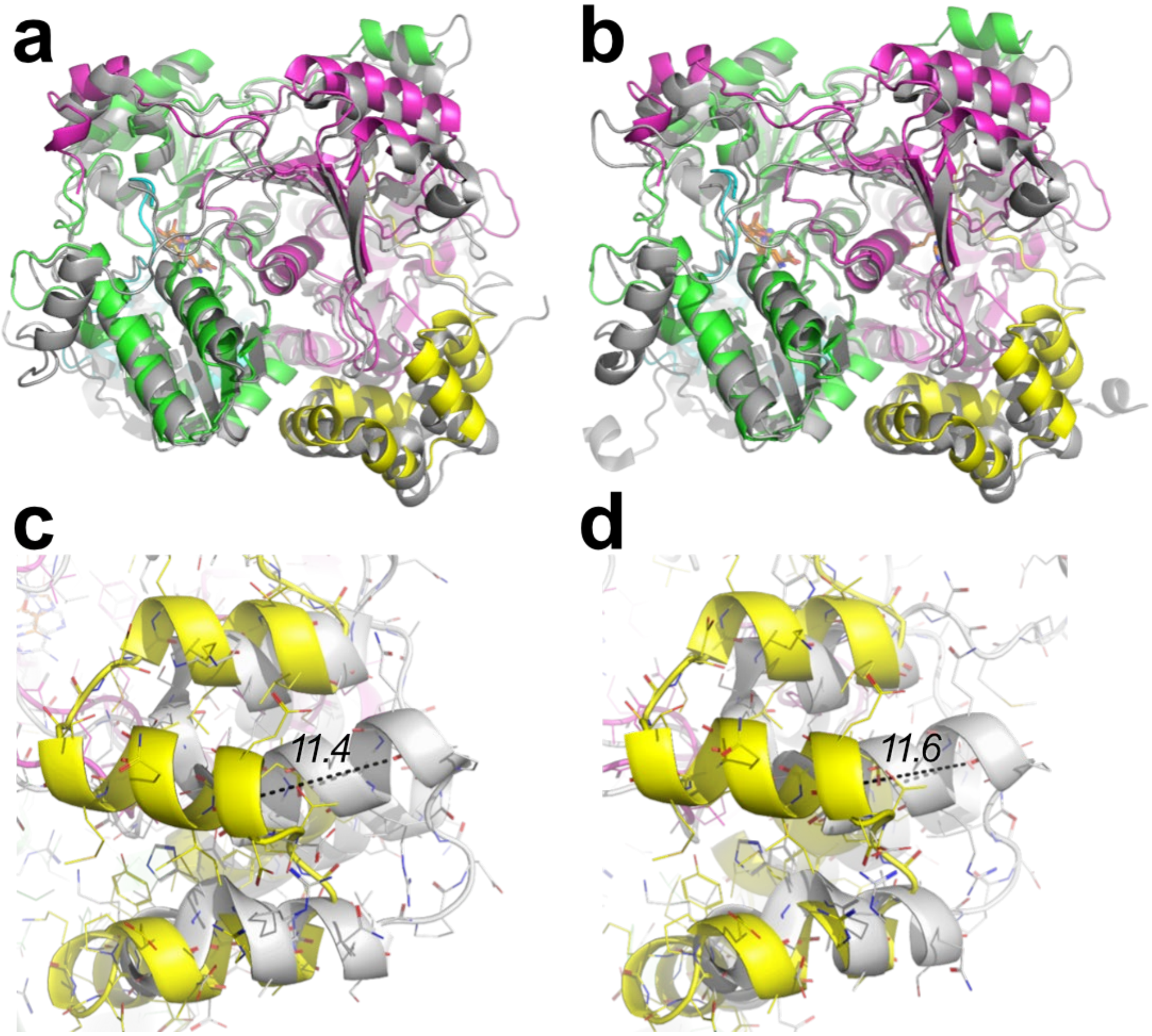

Supplementary Figure 8. Structural overlay of the BBD with other protein domains. The BBD from the SonM—SonA-2Me—SAH complex (PDB: 7LTE) shares structural homology to the BBD of the borosin methyltransferase OphMA[2] (PDB: 5N0X; root means squared distance (RMSD) of 2.3 Å for 306 atoms), to LigA of the protocatuate 4,5-dioxygenase complex LigAB[4] (PDB: 1BOU; RMSD of 1.1 Å for 251 atoms), and to a tethered domain in the gallate dioxygenase DesB[5] (PDB: 3WRB; RMSD of 1.9 Å for 286 atoms). RMSDs (all atoms) were calculated using the 'super' function in PyMOL. The close structural homology is in contrast to the relatively low pairwise sequence identity of 24.3% and 45.3% sequence similarity amongst these domains.

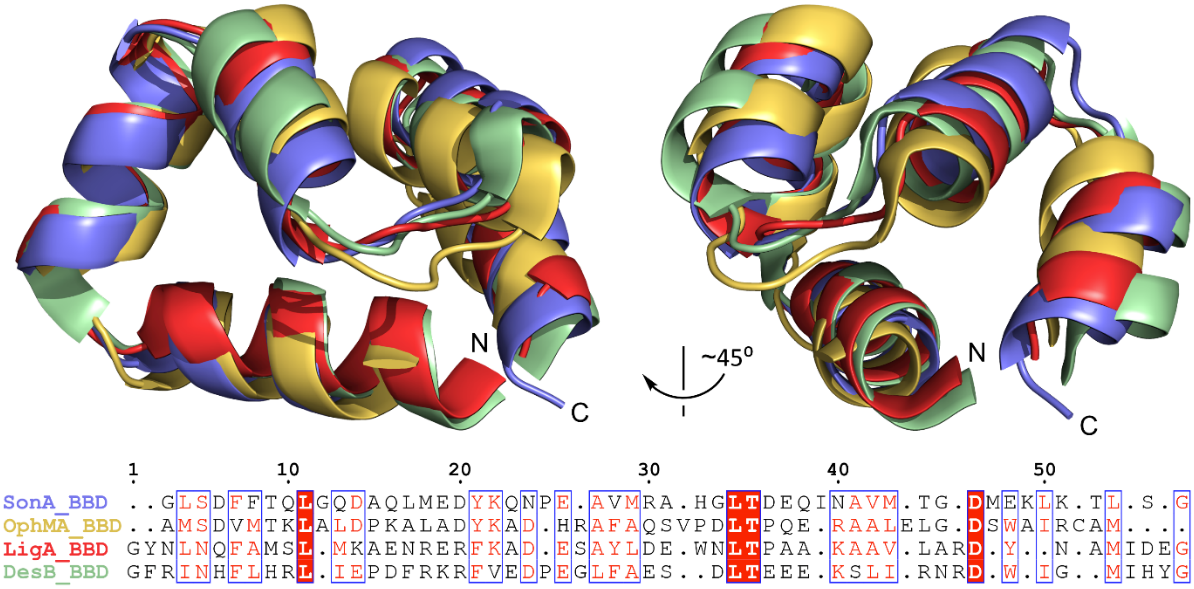

Supplementary Figure 9. Thermal motion B-factors for SonA and SonM. Putty cartoon representations of the thermal motion B-factor variation for (**a**) SonA and (**b**) SonM in the structure SonM—SonA-2Me—SAH (PDB: 7LTE). B-factors are represented in a rainbow-color spectrum of dark blue (lowest mobility) to dark red (highest mobility).

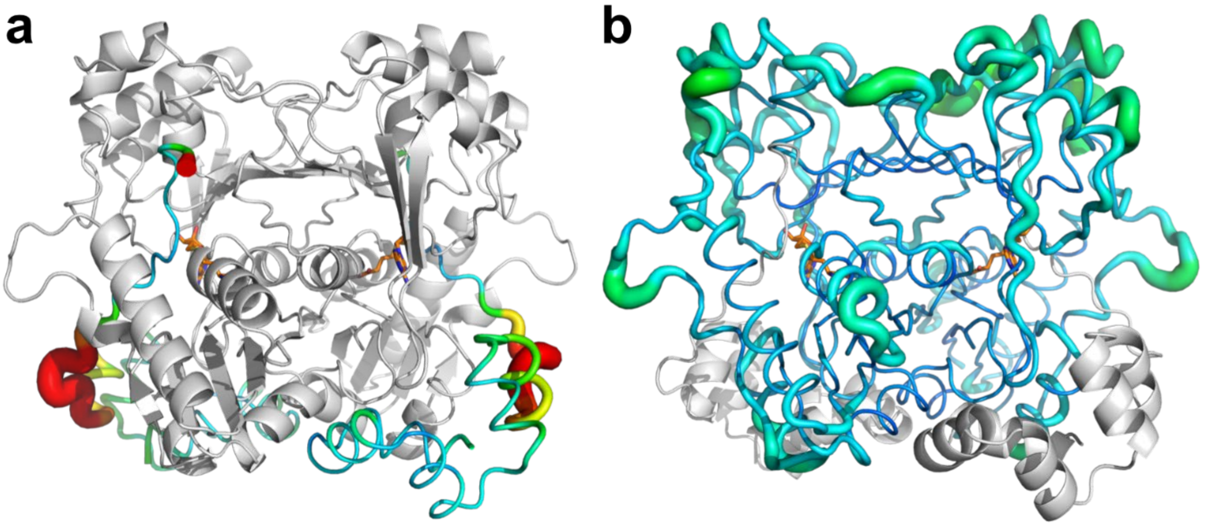

Supplementary Figure 10. SonM—SonA-2Me—SAH active site coordination. (**a**) SonM-Y71 and SonM-Y58 (green cartoon and sticks) coordinates with the 'i+1', 'i', and 'i-1' residues of the SonA core peptide (slate cartoon and sticks). Key distances are depicted as black dashed lines and their lengths noted in italics in Ångstroms. (**b**) Interestingly, the Ψ angle between residues 'I' and 'i+1' is 29.9° and consequently the main chain twists 101° (angle between 'i+1', 'I' and 'i-1' α-carbon atoms). A similar observation was made in OphMA.[2] Ψ torsion angles values of ± 30° were reported to create electronic distortion in amide bonds that could increase the reactivity of the backbone NH group,[6] and could therefore help catalysis. This specific conformation may result from the active site pre-organization and is stabilized by a network of interactions between the side chains of SonM-Y71 and SonM-Y58 and SonA's main chain, and particularly with the carbonyl group of 'i-1', but also with a hydrogen bond between the carbonyl group of residue 'i' and the backbone NH of SonM-C101. Of note, the backbone NH group of SonM-C101 is located at the N-termini of an α-helix, and helix macrodipoles have been found to be involved in stabilizing interactions.[7] Because we obtained the structure with the fully methylated peptide (i.e. post-catalysis), the twisted conformation of the main chain could also result from the presence of the methyl group. In fact, the Ψ angle between residues 'i-1' and 'i-2' is 23.5°, and also lead to a substantial twist in the main chain (108° angle between 'i', 'i-1' and 'i-2' α-carbon atoms).

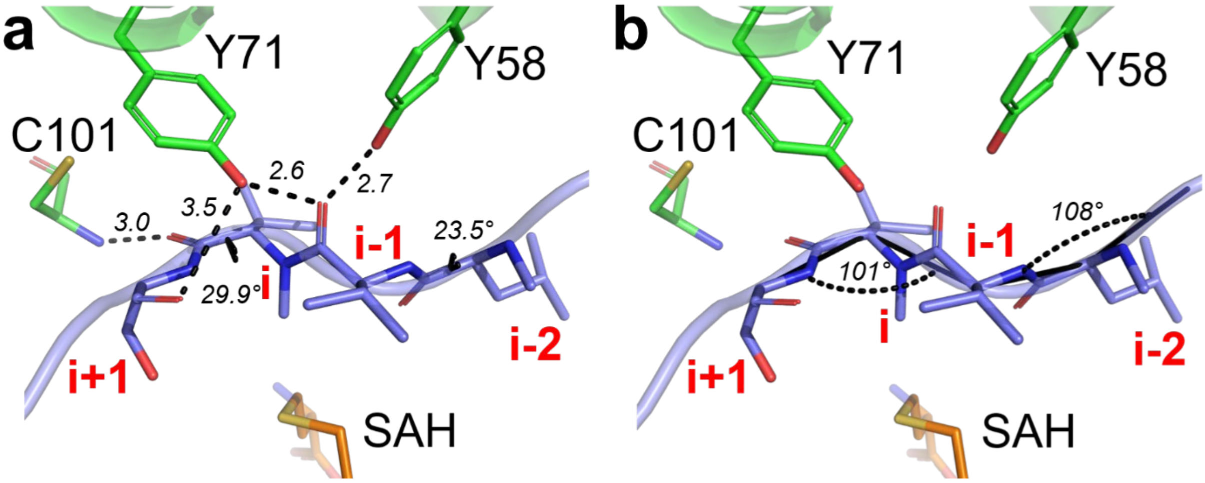

Supplementary Figure 11. SonM interactions with the SonA core peptide in SonM—SonA-2Me—SAH. (**a**) The side chain of residue 'i' of SonA (slate sticks and cartoon) sits in a well-defined pocket formed with SonM-A100, SonM-V102, SonM-L92, and SonM-L34 (green sticks and cartoon). Key distances are depicted as black dashed lines and their lengths noted in italics in Ångstroms. (**b**) Other core peptide side chains are accommodated by less defined binding pockets: the side chain of residue 'i-1' sits in a large cavity formed by polar and apolar residues (SonM-Q167, SonM-I170, SonM-R67, and i-3's side chain SonA-Y62) and the side chain of 'i-2' (SonA-MLE63) sits in a large hydrophobic pocket, while 'i+1' (SonA-S66) sits in a hydrophilic pocket and is hydrogen bonded to SonM-Q167 and SonM-F99. (**c**) Residues 'i+3' to 'i+7' are exposed to the solvent, and so are residues from 'i-3' to the N-termini of the BBD. Residue 'i+4' (SonA-N69) interacts with the carbonyl group of SonM-H174 (3.0 Å). SonM-R68, in addition to its interaction with the carbonyl groups of 'i+5' and the C-termini ('i+6'), it also interacts with the side chain of 'i+6' (SonA-D71) and therefore may contribute to the stabilization of the bound core peptide.

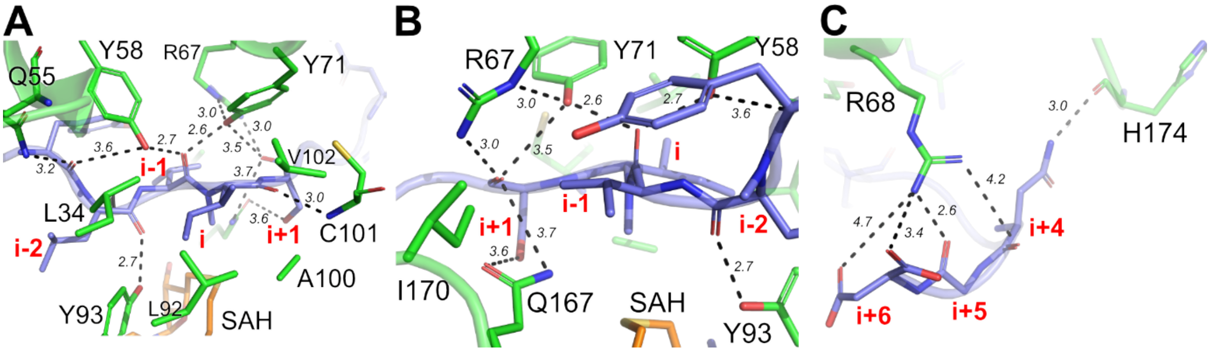

Supplementary Figure 12. Superposition of SonM—SonA-2Me complexes. The structure of *apo* SonM—SonA-2Me (grey cartoon, PDB: 7LTC) is highly similar to SonM—SonA-2Me—SAH (green cartoon, PDB: 7LTE), with an RMSD of 0.19 Å for 4638 atoms. The RMSD was calculated using the 'super' function in PyMOL. SAH is shown as orange sticks.

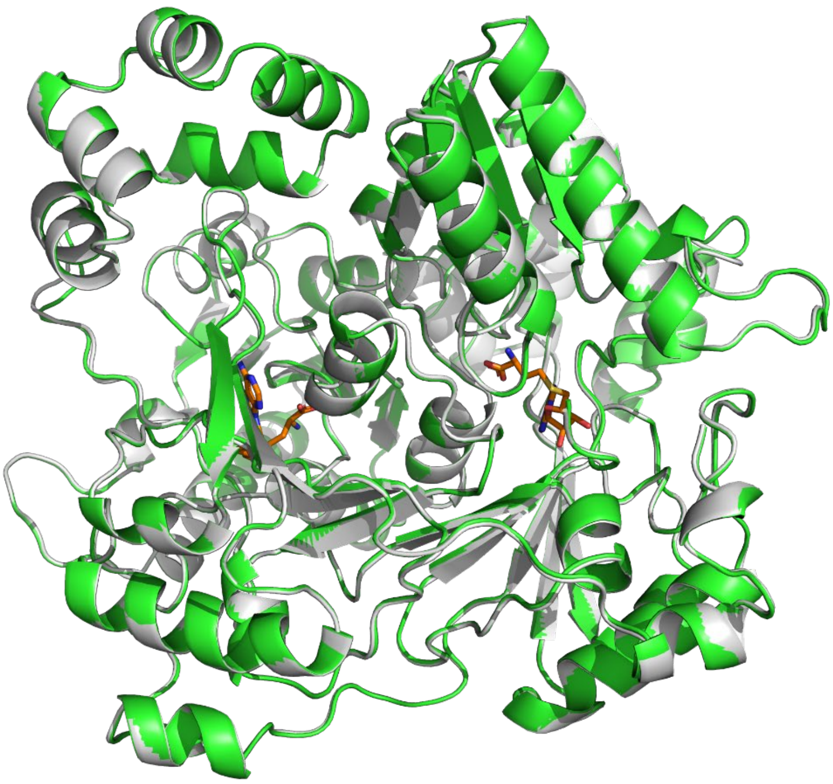

Supplementary Figure 13. SAH makes extensive contacts in the SonM—SonA-2Me—SAH complex. A LIGPLOT[8] of the extensive network of interactions made by SAH in SonM—SonA-2Me—SAH (PDB: 7LTE). SAH and key residues are displayed as ball and sticks, while other contacts are displayed as 'eyelashes'. Key distances are depicted as green dashed lines and their lengths noted in italics in Ångstroms.

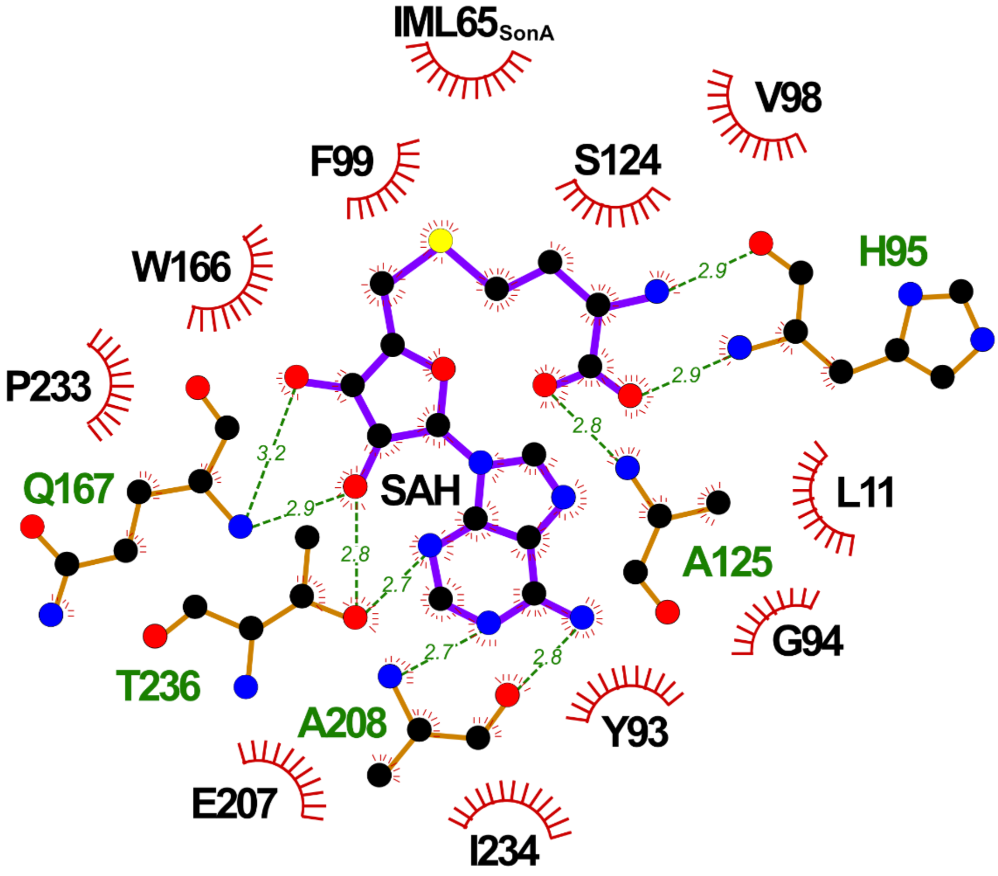

Supplementary Figure 14. SonM – fitted Michaelis-Menten kinetic curves. Michaelis-Menten substrate velocity curves of (**a**) wt SonM and (**b**) SonM mutants with varied [SonA] and saturating [SAM] (left) or varied [SAM] and saturating [SonA] (right). Each substrate concentration was assayed in triplicate (n=3); the enzyme assayed for each set of experiments is listed in the y-axes. The plotted point is the mean velocity measurement at that substrate concentration with the error bars representing the standard deviation between replicates. The overlaid curves were fit using nonlinear regression models in GraphPad Prism 8 and used to determine kinetic constants. The R^2 value for the fitted curve is included at the bottom right of each graph. No kinetics data is shown for SonM-R67A or SonM-Y58F/Y71F as these mutants had no measurable activity under the conditions of the kinetics assay used in this work. Source data are provided as a Source Data file.

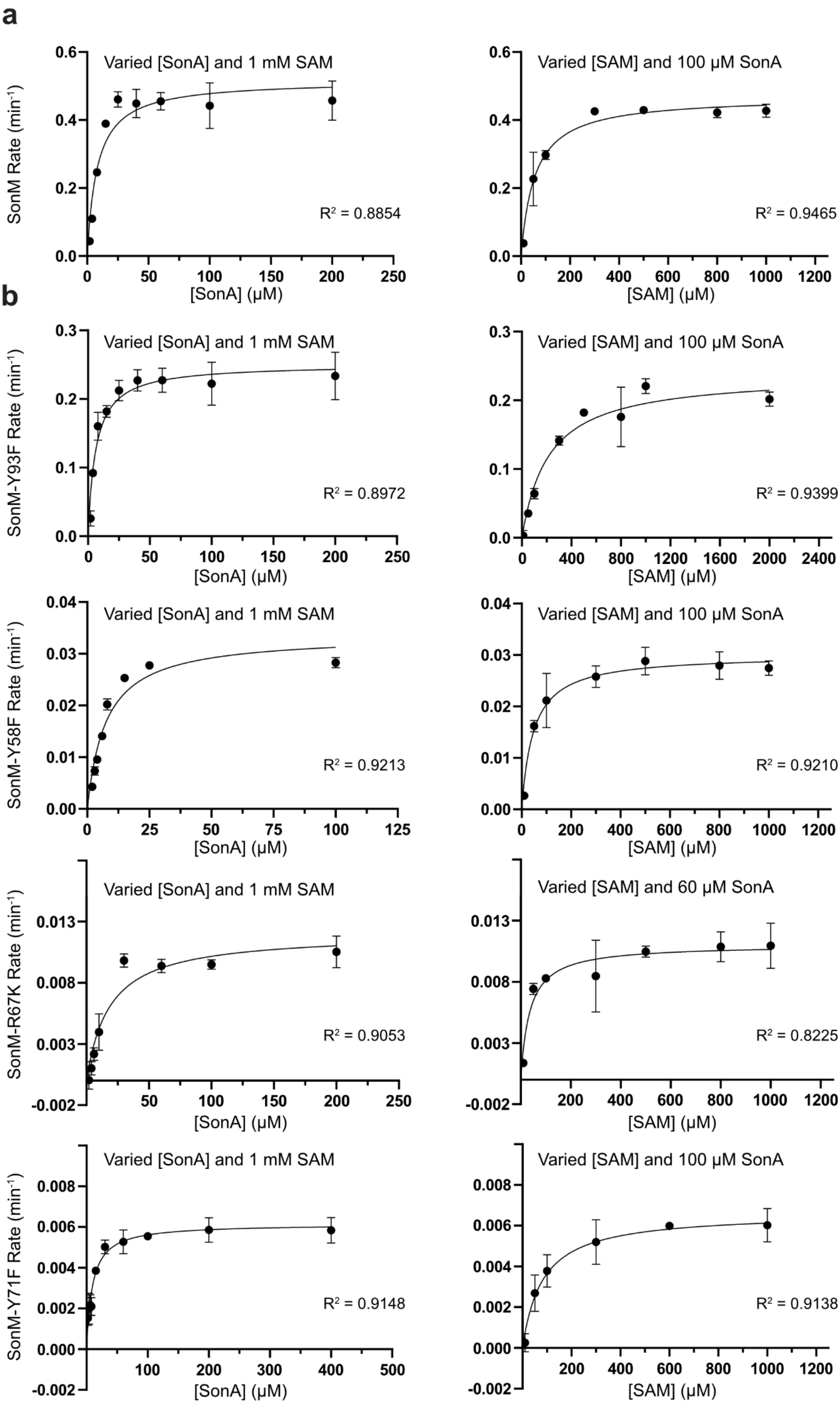
a
b
Varied [SonA] and 1 mM SAM
Varied [SAM] and 100 µM SonA
Varied [SAM] and 60 µM SonA
SonM Rate (min-1)
SonM-Y93F Rate (min-1)
SonM-Y58F Rate (min-1)
SonM-R67K Rate (min-1)
SonM-Y71F Rate (min-1)
[SonA] (µM)
[SAM] (µM)
R2 = 0.8854
R2 = 0.9465
R2 = 0.8972
R2 = 0.9399
R2 = 0.9213
R2 = 0.9210
R2 = 0.9053
R2 = 0.8225
R2 = 0.9148
R2 = 0.9138

Supplementary Figure 15. Active site tyrosine mutant structures. (**a**) Superposition of the SonM-Y93F—SonA-2Me mutant structure complex (green, dark blue, PDB: 7LTH) with wt SonM—SonA-2Me—SAH (grey, cyan, PDB: 7LTE) The RMSD (all atoms) between the two structures is 0.16 Å for 4539 atoms, when using the 'super' function in PyMOL. (**b**) Superposition of the SonM-Y58F—SonA-2Me mutant structure complex (green, dark blue, PDB: 7LTF) with wt SonM—SonA-2Me—SAH (grey, cyan, PDB, 7LTE). The RMSD (all atoms) between the two structures is 0.08 Å for 4824 atoms, when using the 'super' function in PyMOL. Key residues are shown as sticks and key distances are depicted as black dashed lines, with their lengths noted in italics in Ångstroms. Both residues SonM-Y71 and SonM-Y58 are involved in an extensive network of interaction with the core peptide, including hydrogen bonding to the carbonyl group of 'i-1' SonM-IML65. This configuration is similar to OphMA, where the corresponding tyrosines OphMA-Y66 and OphMA-Y76 were proposed to stabilize sp^3 hybridization and the developing negative charge on the carbonyl's oxygen atom (oxyanion hole).[2] We note that in the SonM—SonA-2Me—SAH structure, the interaction angles between the carbonyl group and the hydroxyl groups of SonM-Y58 and SonM-Y71 are 122° and 108°, respectively. The interaction angle with SonM-Y71 is therefore close to the canonical angle value of 109.5° for sp^3 hybridization to the carbonyl group.

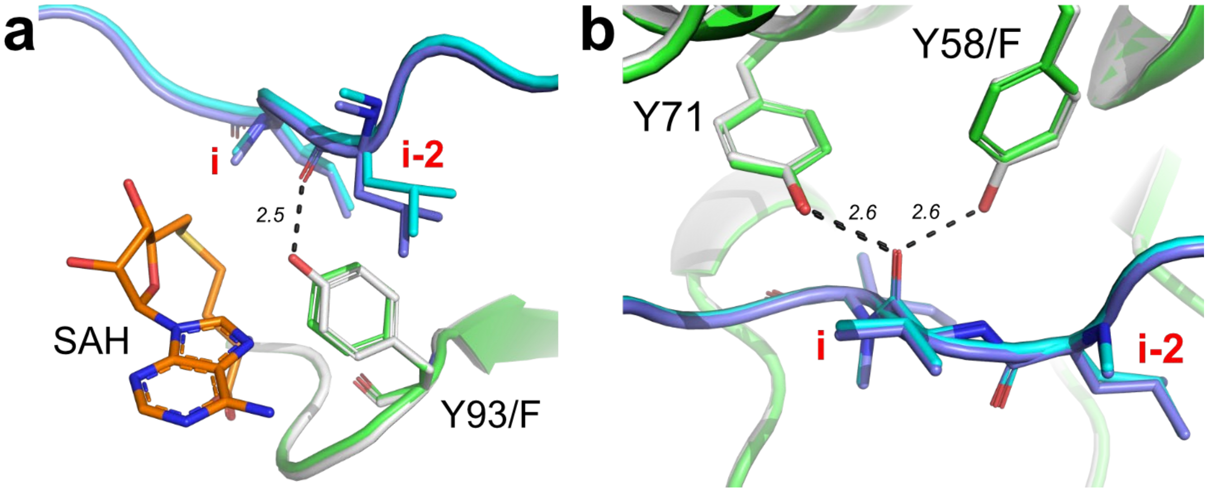

Supplementary Figure 16. SonM in vitro reactions analyzed by LC-MS/MS and compared to kinetic model simulations. Relative abundances for each species of SonA (SonA-0Me , SonA-1Me, SonA-2Me) are depicted as extracted ion chromatograms from LC-MS data after incubation at 30°C with wt SonM, saturating [SAM], and the other enzymes and kit reagents used in the kinetics assay (see Methods section). All reactions were set up in duplicate under the same conditions and were quenched at time points as indicated on the left of each set of plots. The amino acid sequence of the AspN digested fragment is shown at the top with the methylated residues in orange with an asterisk (*). The methylation state is indicated over each graph (0-2) in an orange box with the relative abundance (%) of the methylated species directly below. Relative abundance (intensity %) was determined by integrating under each peak from the extracted ion chromatogram. Each peak is plotted over its retention time (x-axis). This data is displayed in each panel of Fig. 4.

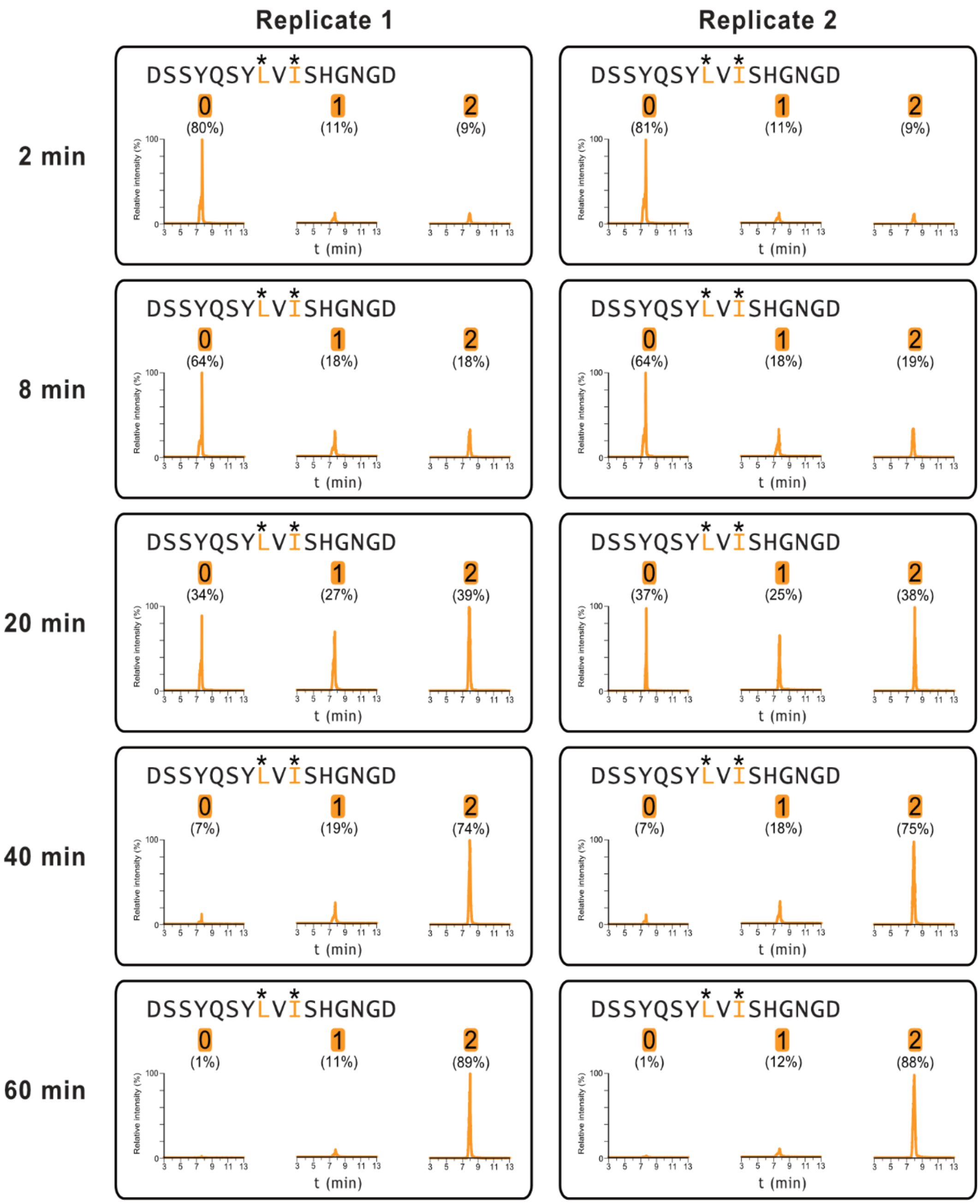

Supplementary Figure 17. SonM – fitted Michaelis-Menten competitive inhibition kinetic curves. Competitive inhibition curves for wt SonM with varied [SonA] saturating [SAM] and increasing (**a**) [SonA-BBD] or (**b**) [SonA-2Me], respectively. Each substrate concentration was assayed in triplicate (n=3). The plotted point is the mean velocity measurement at that substrate concentration with the error bars representing the standard deviation between replicates. The overlaid curves were fit using nonlinear regression models in GraphPad Prism 8 and used to determine kinetic constants. The R^2 value for the fitted curve is included at the bottom right of each graph. Source data are provided as a Source Data file.

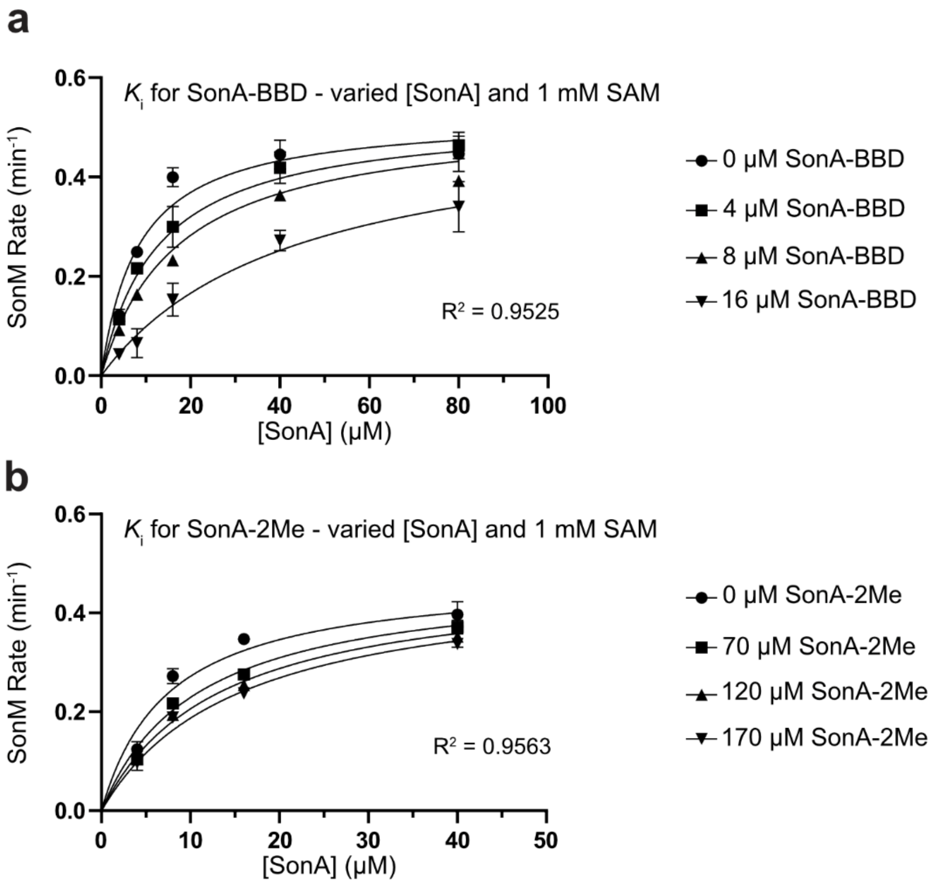

Supplementary Figure 18. Superposition of SonM—SonA-2Me and SonM—SonA-BBD—(±)SAM complexes. The SAM-bound heterodimer of SonM—SonA-BBD—(±)SAM (green and beige cartoon, PDB: 7LTR) is similar to SonM—SonA-2Me—SAH (grey and dark grey cartoon, PDB: 7LTE). SAM/SAH is shown as orange sticks.

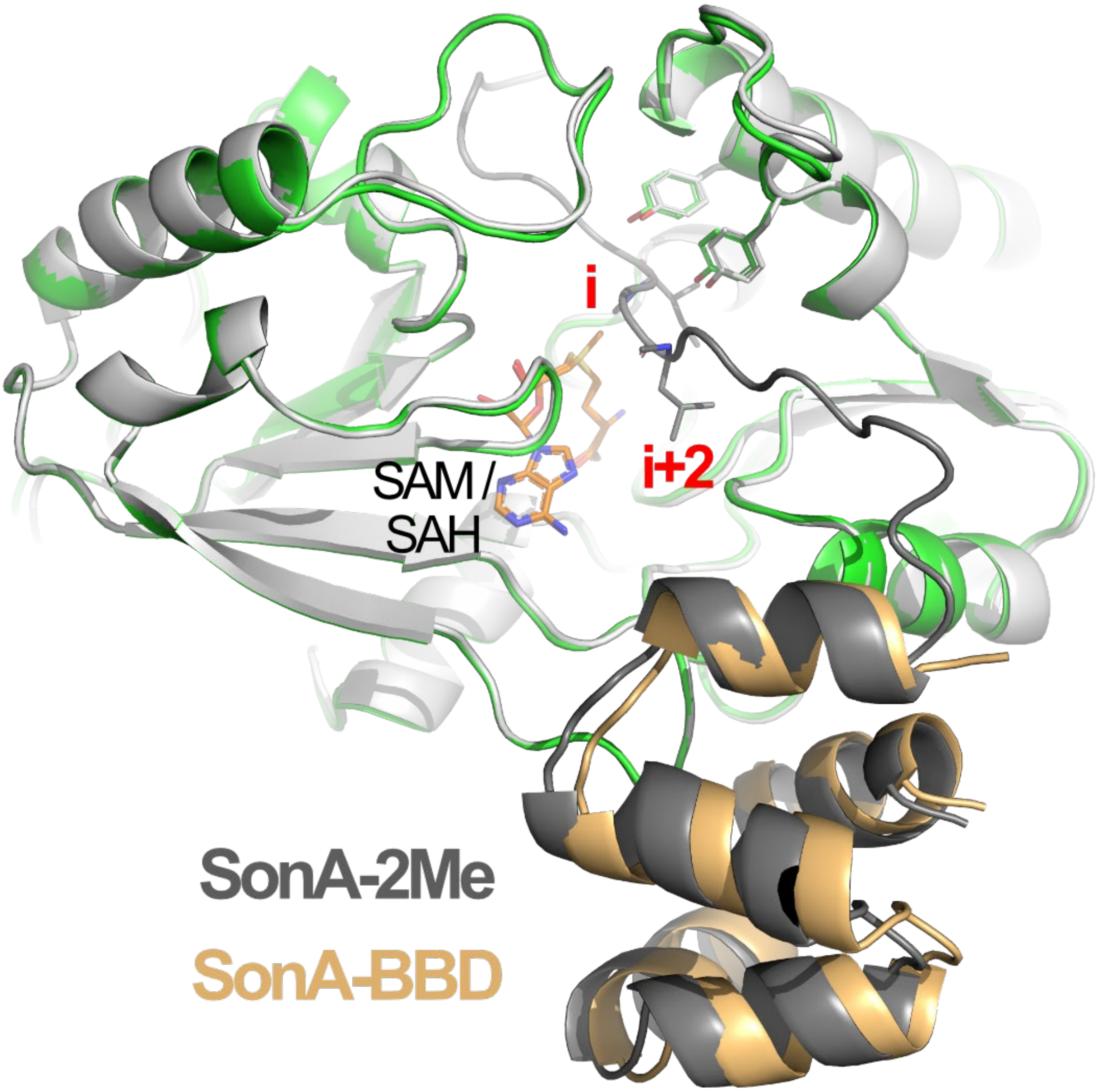

Supplementary Figure 19. Bottom lock configurations of the SonM—SonA-BBD—(±)SAM complex. (**a**) Closed and (**b**) open *bottom lock* configurations in SonM—SonA-BBD—(±)SAM (PDB: 7LTR).

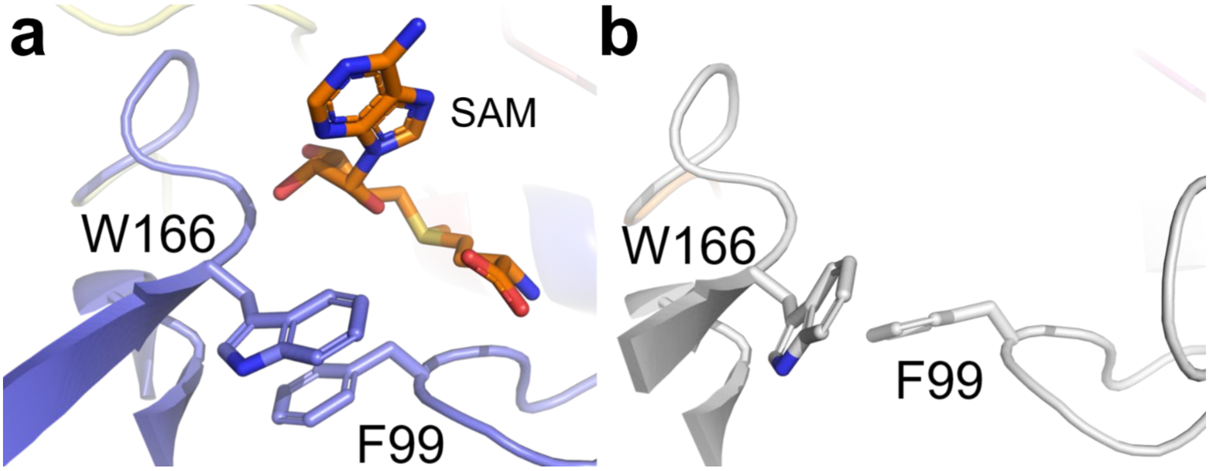

Supplementary Figure 20. Top lock configurations between borosin α-*N*-methyltransferases. (**a**) View of the R67 active site interaction network of SonM—SonA-2Me—SAH (PDB: 7LTE) among residues in the top clamp, side clamp, and SonA core peptide. (**b**) View of the equivalent network of the homologous residue R72 in OphMA (PDB: 6MJG).

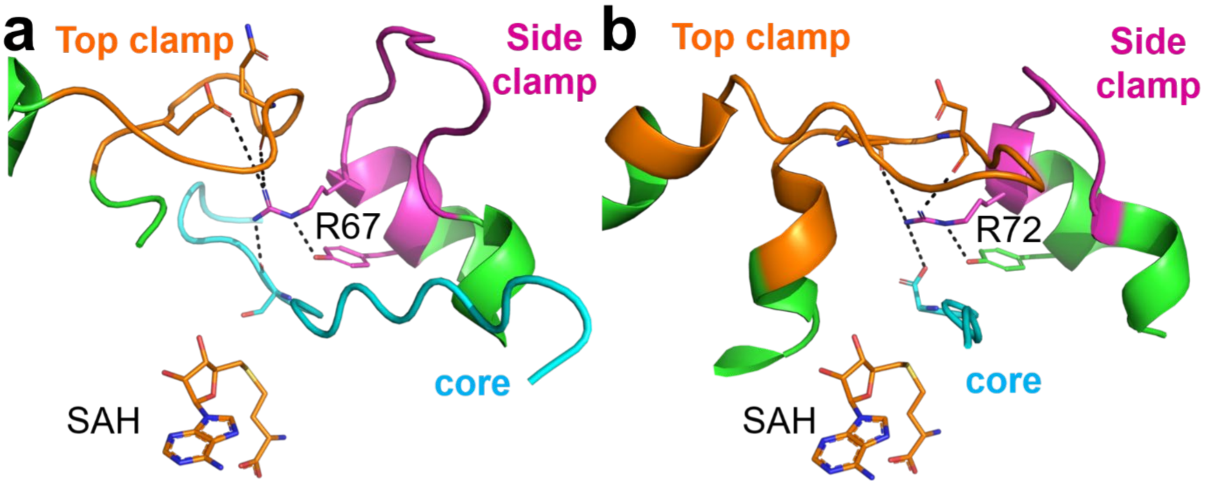

Supplementary Figure 21. Active site configuration differences among split borosin complexes. Superposition between the SonM—SonA-2Me—SAH (grey and teal cartoon, PDB: 7LTE), SonM-R67A—SonA-0Me—SAH (green and slate cartoon, PDB: 7LTS), and the heterodimer not bound to cofactor in SonM—SonA-BBD—(±)SAM (maroon cartoon, PDB: 7LTR). In addition to the signifcant change in core peptide conformation, the top and side clamps of SonM-R67A—SonA-0Me—SAH are in intermediate positions as compared to the two other structures.

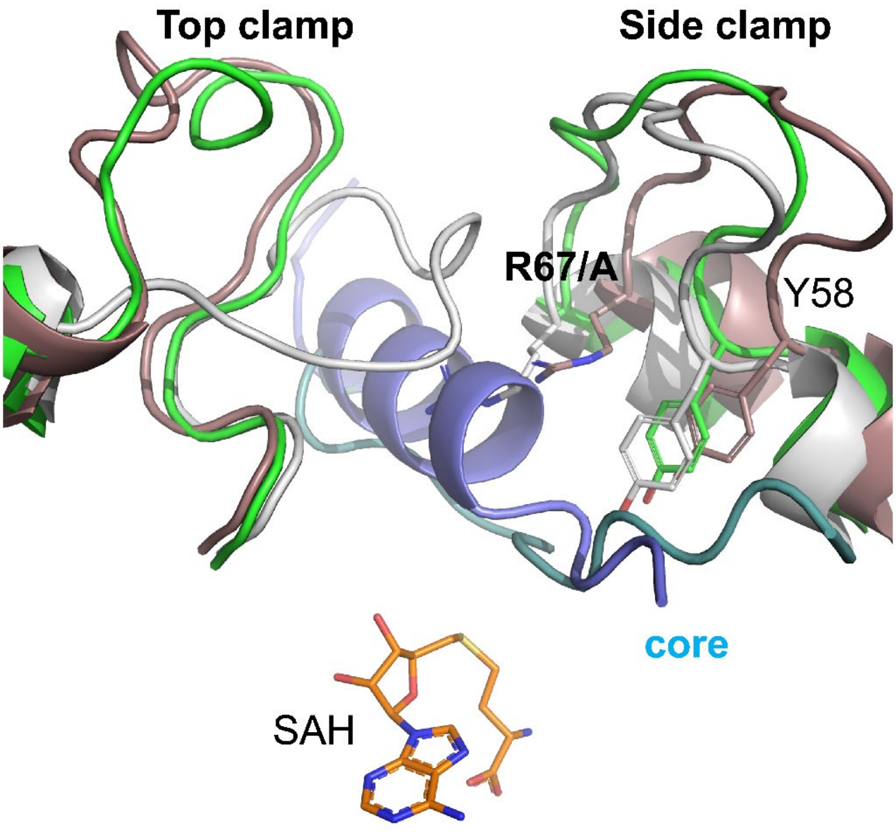

Supplementary Figure 22. Interaction networks in different configurations of SonM and mutant complexes. (**a**) Interaction network of R185 in the SonM—SonA-2Me—SAH (PDB: 7LTE) compared to the synonymous network in (**b**) SonM-R67A—SonA-0Me—SAH (PDB: 7LTS) and (**c**) Interaction network of R185 in the heterodimer not bound to cofactor in SonM—SonA-BBD—(±)SAM (PDB: 7LTR). Similarly to SonM—SonA-BBD, SonM-R185 rotates ~5 Å and interacts with SonM-E173 (5.8 Å), possibly compensating for the loss of the SonM-R67—SonM-E173 interaction. SonM-R185 also interacts with SonM-S182 (3.8 Å) and the carbonyl groups of SonM-A169 (3.5 Å), SonM-A171 (3.2 Å), and SonM-H180 (3.9 Å), contributing to stabilize the open top clamp conformation (~14 Å). Key distances are depicted as black dashed lines and their lengths noted in italics in Ångstroms.

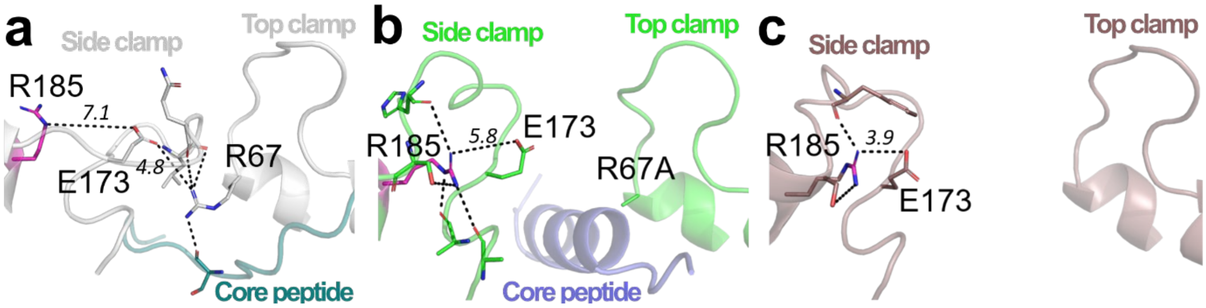

Supplementary Figure 23. Structural differences in the heterodimers of SonM-R67A—SonA-0Me—SAH. Superposition of the two SonM-R67A—SonA-0Me—SAH heterodimers (green/cyan and maroon/slate cartoons, PDB: 7LTS). The RMSD (all atoms) between the two structures is 0.32 Å for 2000 atoms, when using the 'super' function in PyMOL. Small differences can be observed in the configuration of SonA as well the top and side clamps in SonM-R67A.

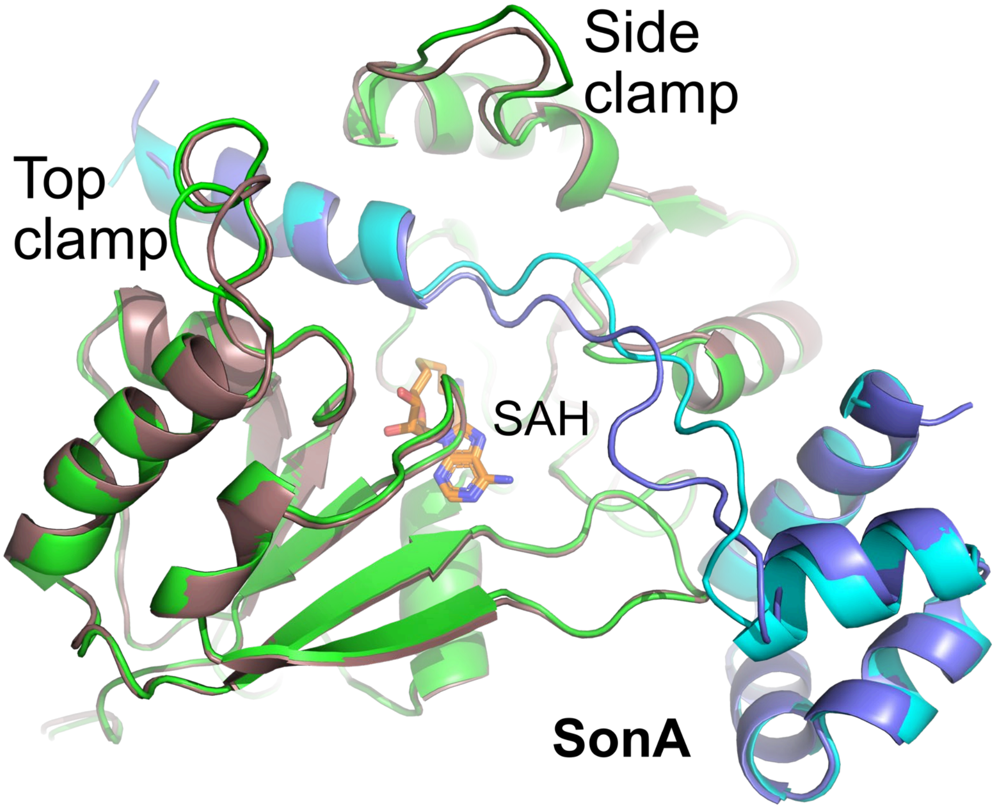

Supplementary Figure 24. Structural differences in the BBD of SonM-R67A—SonA-0Me—SAH and *apo* SonM—SonA-2Me—SAH. Superposition of SonM-R67A—SonA-0Me—SAH (grey and cyan cartoon, PDB: 7LTS) with *apo* SonM—SonA-2Me—SAH (green and slate cartoon, PDB: 7LTE). Helix 5 of the BBD is unwound in the SonM-R67A—SonA-0Me—SAH structure. Key distances are depicted as black dashed lines and their lengths noted in italics in Ångstroms.

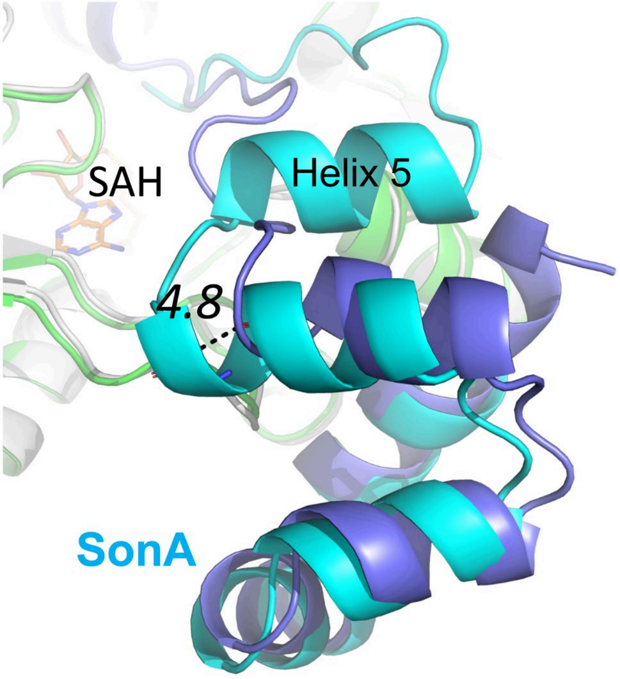

Supplementary Figure 25. Mass spectrometric analysis of SonM mutant in vitro reactions. HPLC-MS/MS spectra of the highest methylated species from AspN-digested SonA peptides after incubation at 30°C with the listed SonM mutant (**a**) SonM-Y93F, (**b**) SonM-R67K, (**c**) SonM-R67A, (**d**) SonM-Y58F, (**e**) SonM-Y71F, and (**f**) SonM-Y58F/Y71F, saturating [SAM], and the other enzymes and kit reagents used in the kinetics assay (see materials and methods). The amino acid sequence above each spectra depicts the *N*-methylated residues that could be confirmed by MS/MS fragmentation (solid orange circles) or are inferred *N*-methylated since the position is not completely defined by MS/MS (unfilled orange circles). Observed MS/MS fragmentation masses are listed above (b-ions) and below (y-ions) the amino acid sequence. The gray lines within the sequence mark the sites of fragmentation. Masses of methylation-containing ions are denoted in brackets, where 'Me' stands for methylation. The ppm difference from the observed masses to the theoretical expected masses are labeled in parentheses. A 10.0-ppm mass cutoff for annotated HPLC-MS/MS peaks was used. The protein, time of *in vitro* reaction, parent ion information and HPLC retention time (RT) are listed in the upper right corner of the LC-MS/MS spectra. Off-target methylations were not detected in any sample.

a

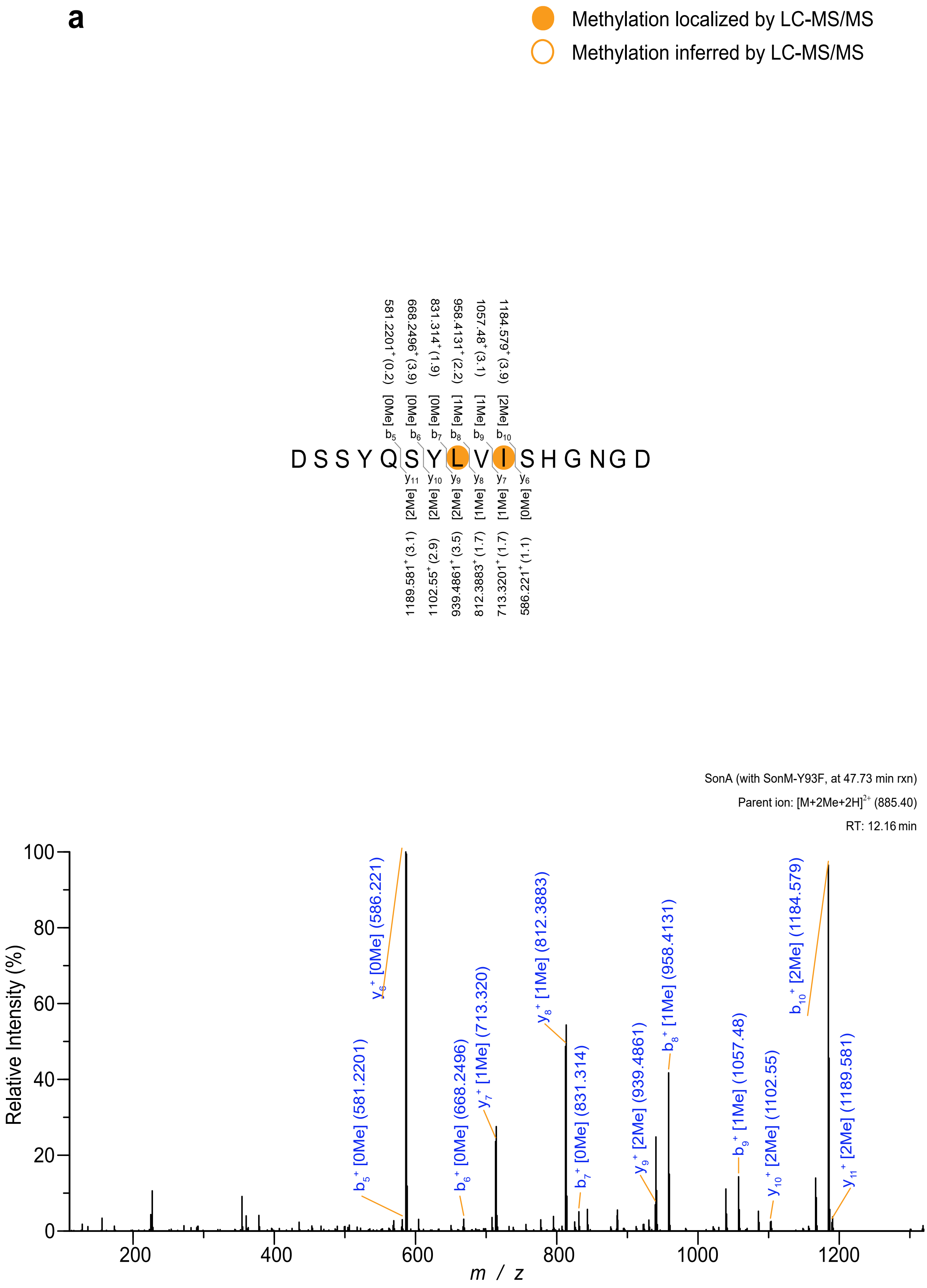
Methylation localized by LC-MS/MS
Methylation inferred by LC-MS/MS
D S S Y Q S Y L V I S H G N G D
581.2201+ (0.2) [0Me] b5
668.2496+ (3.9) [0Me] b6
831.314+ (1.9) [0Me] b7
958.4131+ (2.2) [1Me] b8
1057.48+ (3.1) [1Me] b9
1184.579+ (3.9) [2Me] b10
y11 [2Me] 1189.581+ (3.1)
y10 [2Me] 1102.55+ (2.9)
y9 [2Me] 939.4861+ (3.5)
y8 [1Me] 812.3883+ (1.7)
y7 [1Me] 713.3201+ (1.7)
y6 [0Me] 586.221+ (1.1)
SonA (with SonM-Y93F, at 47.73 min rxn)
Parent ion: [M+2Me+2H]2+ (885.40)
RT: 12.16 min
Relative Intensity (%)
m / z
b5+ [0Me] (581.2201)
y6+ [0Me] (586.221)
b6+ [0Me] (668.2496)
y7+ [1Me] (713.320)
y8+ [1Me] (812.3883)
b7+ [0Me] (831.314)
y9+ [2Me] (939.4861)
b8+ [1Me] (958.4131)
b9+ [1Me] (1057.48)
y10+ [2Me] (1102.55)
b10+ [2Me] (1184.579)
y11+ [2Me] (1189.581)
0
20
40
60
80
100
200
400
600
800
1000
1200

b

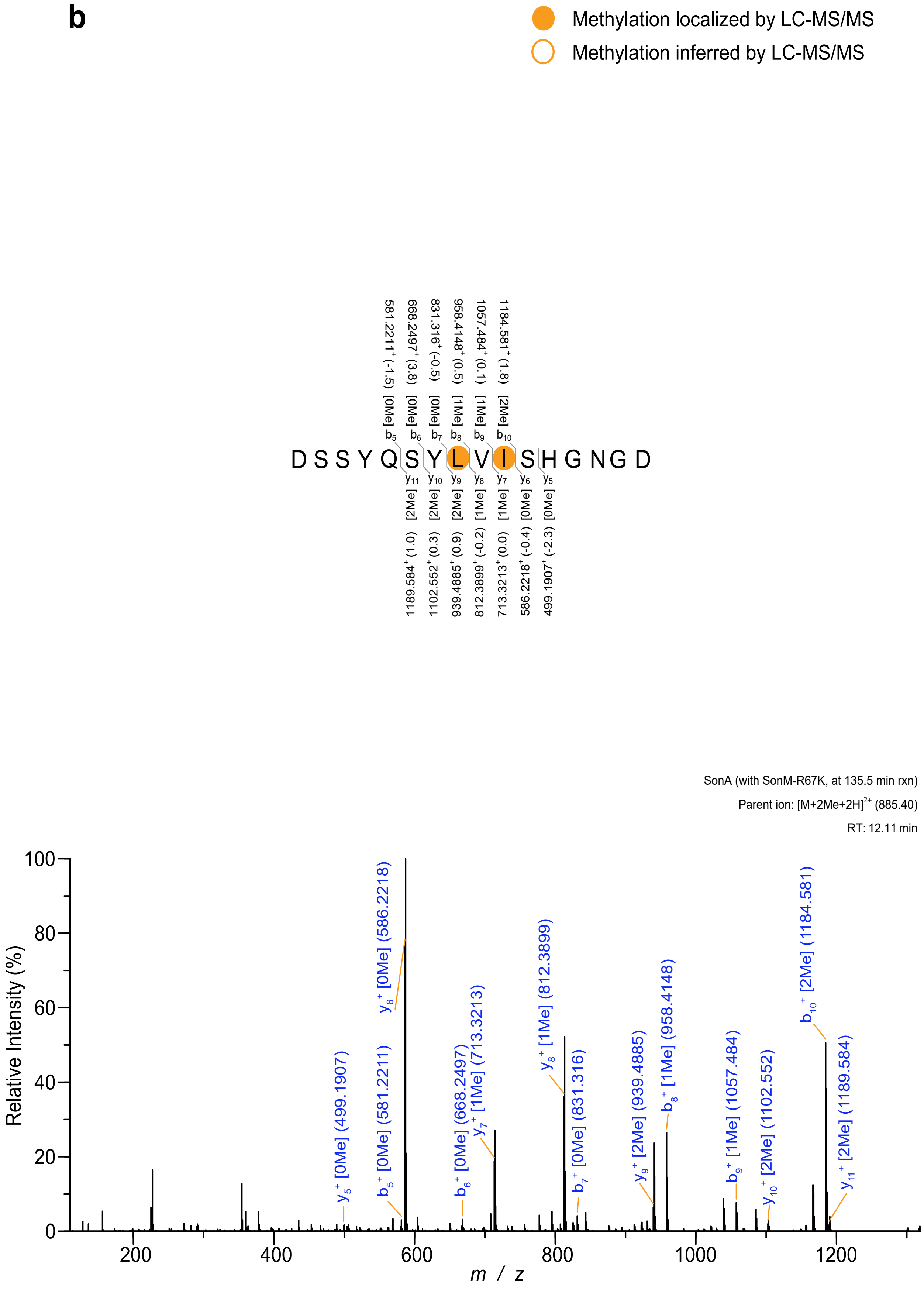
Methylation localized by LC-MS/MS
Methylation inferred by LC-MS/MS
D S S Y Q S Y L V I S H G N G D
581.2211+ (-1.5) [0Me] b5
668.2497+ (3.8) [0Me] b6
831.316+ (-0.5) [0Me] b7
958.4148+ (0.5) [1Me] b8
1057.484+ (0.1) [1Me] b9
1184.581+ (1.8) [2Me] b10
y11 [2Me] (1.0) 1189.584+
y10 [2Me] (0.3) 1102.552+
y9 [2Me] (0.9) 939.4885+
y8 [1Me] (-0.2) 812.3899+
y7 [1Me] (0.0) 713.3213+
y6 [0Me] (-0.4) 586.2218+
y5 [0Me] (-2.3) 499.1907+
SonA (with SonM-R67K, at 135.5 min rxn)
Parent ion: [M+2Me+2H]2+ (885.40)
RT: 12.11 min
Relative Intensity (%)
m / z
y5+ [0Me] (499.1907)
b5+ [0Me] (581.2211)
y6+ [0Me] (586.2218)
b6+ [0Me] (668.2497)
y7+ [1Me] (713.3213)
y8+ [1Me] (812.3899)
b7+ [0Me] (831.316)
y9+ [2Me] (939.4885)
b8+ [1Me] (958.4148)
b9+ [1Me] (1057.484)
y10+ [2Me] (1102.552)
b10+ [2Me] (1184.581)
y11+ [2Me] (1189.584)

c

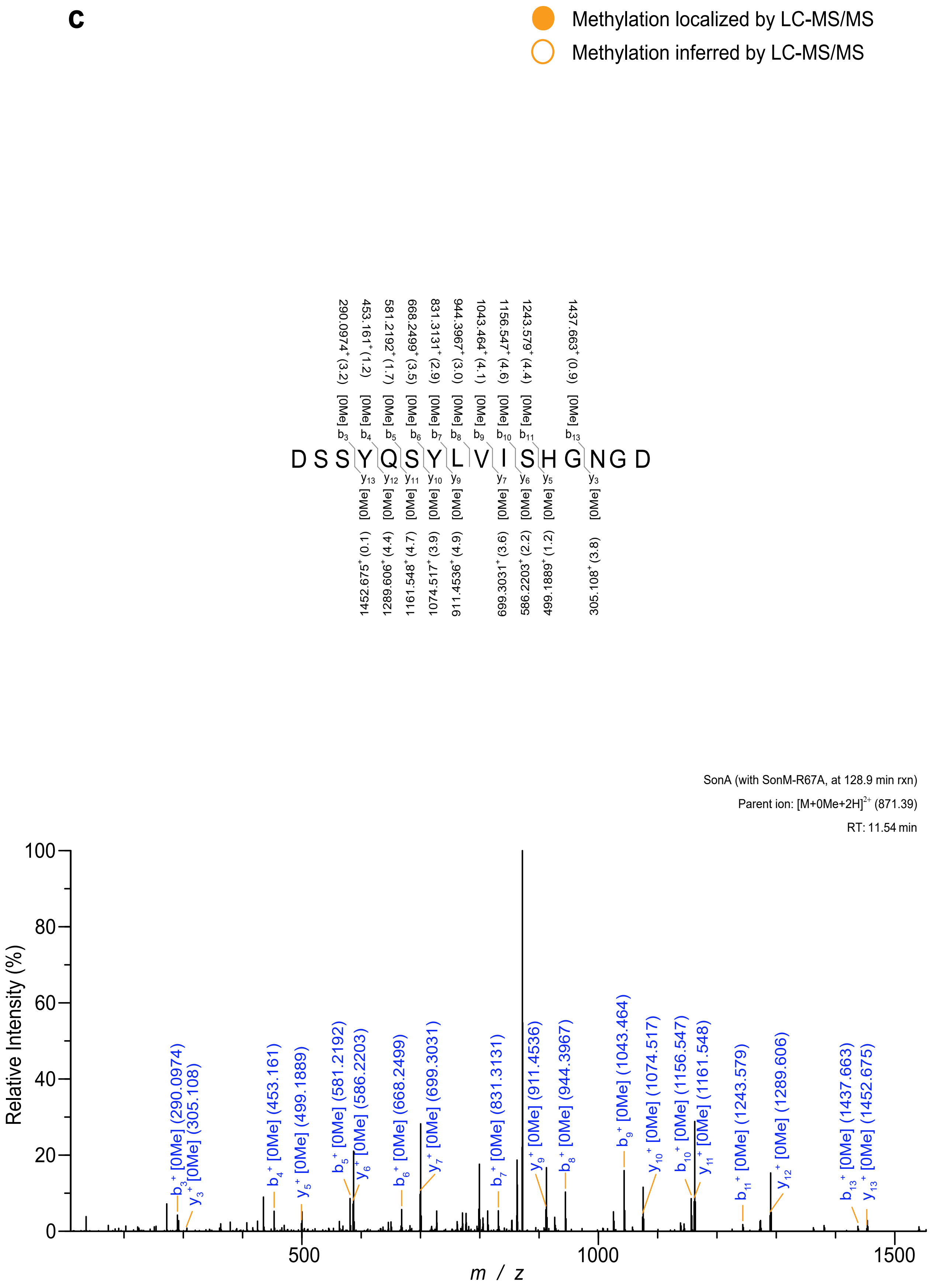

Methylation localized by LC-MS/MS
Methylation inferred by LC-MS/MS
D S S Y Q S Y L V I S H G N G D
290.0974+ (3.2) [0Me] b3
453.161+ (1.2) [0Me] b4
581.2192+ (1.7) [0Me] b5
668.2499+ (3.5) [0Me] b6
831.3131+ (2.9) [0Me] b7
944.3967+ (3.0) [0Me] b8
1043.464+ (4.1) [0Me] b9
1156.547+ (4.6) [0Me] b10
1243.579+ (4.4) [0Me] b11
1437.663+ (0.9) [0Me] b13
y13 [0Me] 1452.675+ (0.1)
y12 [0Me] 1289.606+ (4.4)
y11 [0Me] 1161.548+ (4.7)
y10 [0Me] 1074.517+ (3.9)
y9 [0Me] 911.4536+ (4.9)
y7 [0Me] 699.3031+ (3.6)
y6 [0Me] 586.2203+ (2.2)
y5 [0Me] 499.1889+ (1.2)
y3 [0Me] 305.108+ (3.8)
SonA (with SonM-R67A, at 128.9 min rxn)
Parent ion: [M+0Me+2H]2+ (871.39)
RT: 11.54 min
Relative Intensity (%)
m / z
0
20
40
60
80
100
500
1000
1500
b3+ [0Me] (290.0974)
y3+ [0Me] (305.108)
b4+ [0Me] (453.161)
y5+ [0Me] (499.1889)
b5+ [0Me] (581.2192)
y6+ [0Me] (586.2203)
b6+ [0Me] (668.2499)
y7+ [0Me] (699.3031)
b7+ [0Me] (831.3131)
y9+ [0Me] (911.4536)
b8+ [0Me] (944.3967)
b9+ [0Me] (1043.464)
y10+ [0Me] (1074.517)
b10+ [0Me] (1156.547)
y11+ [0Me] (1161.548)
b11+ [0Me] (1243.579)
y12+ [0Me] (1289.606)
b13+ [0Me] (1437.663)
y13+ [0Me] (1452.675)

d

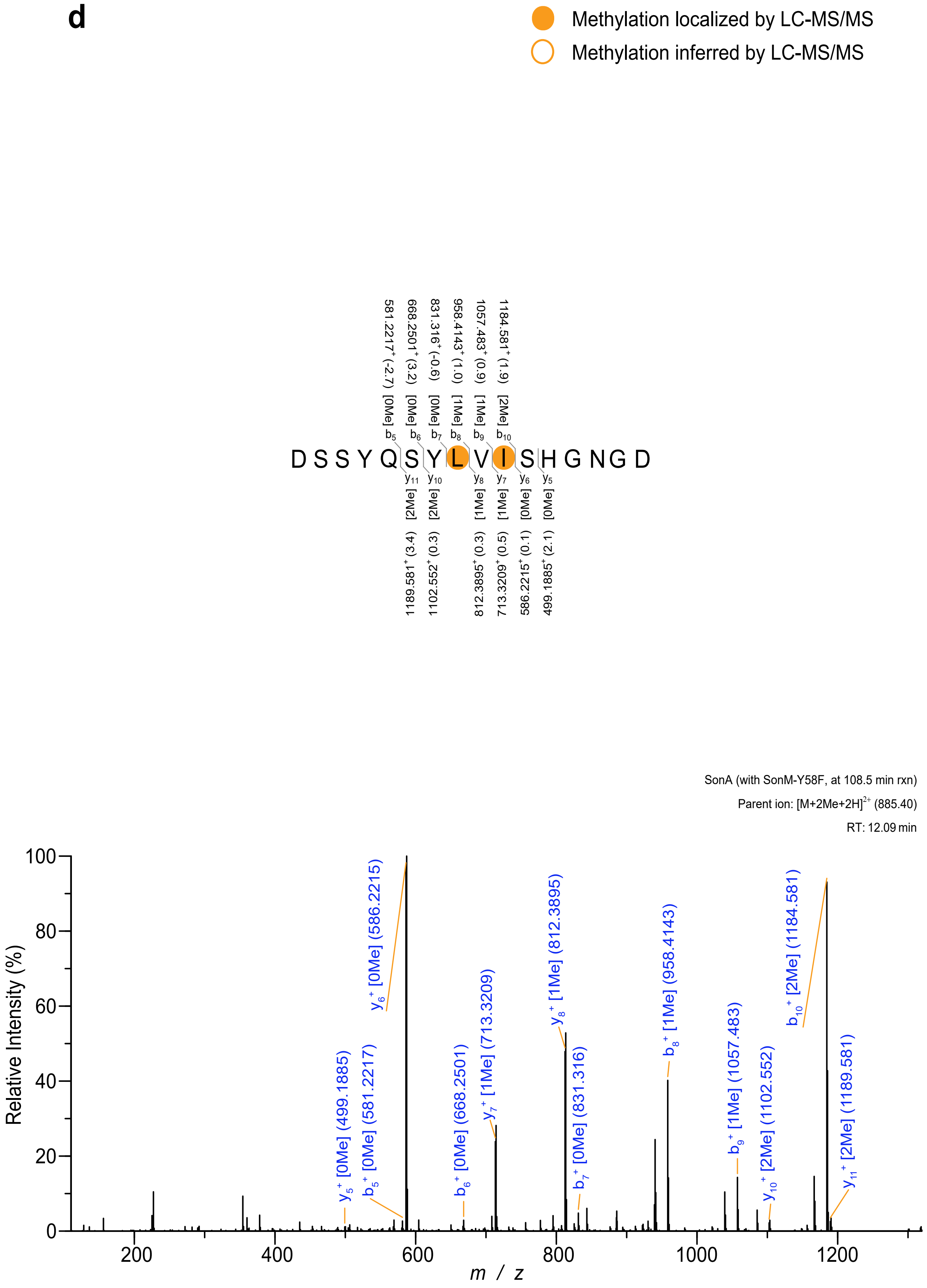

Methylation localized by LC-MS/MS
Methylation inferred by LC-MS/MS
D S S Y Q S Y L V I S H G N G D
SonA (with SonM-Y58F, at 108.5 min rxn)
Parent ion: [M+2Me+2H]2+ (885.40)
RT: 12.09 min
Relative Intensity (%)
m / z

e

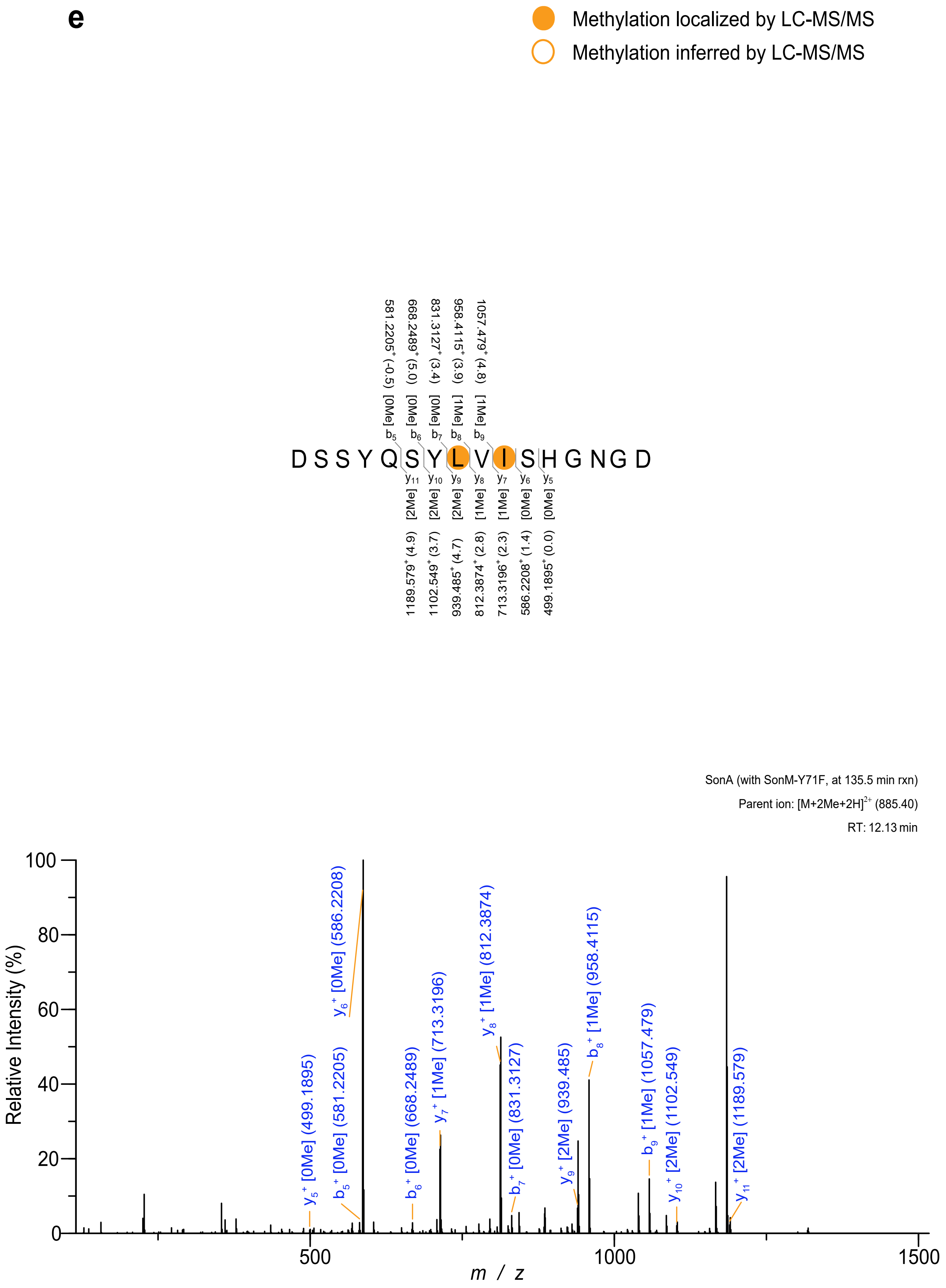

Methylation localized by LC-MS/MS
Methylation inferred by LC-MS/MS
D S S Y Q S Y L V I S H G N G D
b5 [0Me] 581.2205+ (-0.5)
b6 [0Me] 668.2489+ (5.0)
b7 [0Me] 831.3127+ (3.4)
b8 [1Me] 958.4115+ (3.9)
b9 [1Me] 1057.479+ (4.8)
y11 [2Me] 1189.579+ (4.9)
y10 [2Me] 1102.549+ (3.7)
y9 [2Me] 939.485+ (4.7)
y8 [1Me] 812.3874+ (2.8)
y7 [1Me] 713.3196+ (2.3)
y6 [0Me] 586.2208+ (1.4)
y5 [0Me] 499.1895+ (0.0)
SonA (with SonM-Y71F, at 135.5 min rxn)
Parent ion: [M+2Me+2H]2+ (885.40)
RT: 12.13 min
Relative Intensity (%)
m / z
y5+ [0Me] (499.1895)
b5+ [0Me] (581.2205)
y6+ [0Me] (586.2208)
b6+ [0Me] (668.2489)
y7+ [1Me] (713.3196)
y8+ [1Me] (812.3874)
b7+ [0Me] (831.3127)
y9+ [2Me] (939.485)
b8+ [1Me] (958.4115)
b9+ [1Me] (1057.479)
y10+ [2Me] (1102.549)
y11+ [2Me] (1189.579)

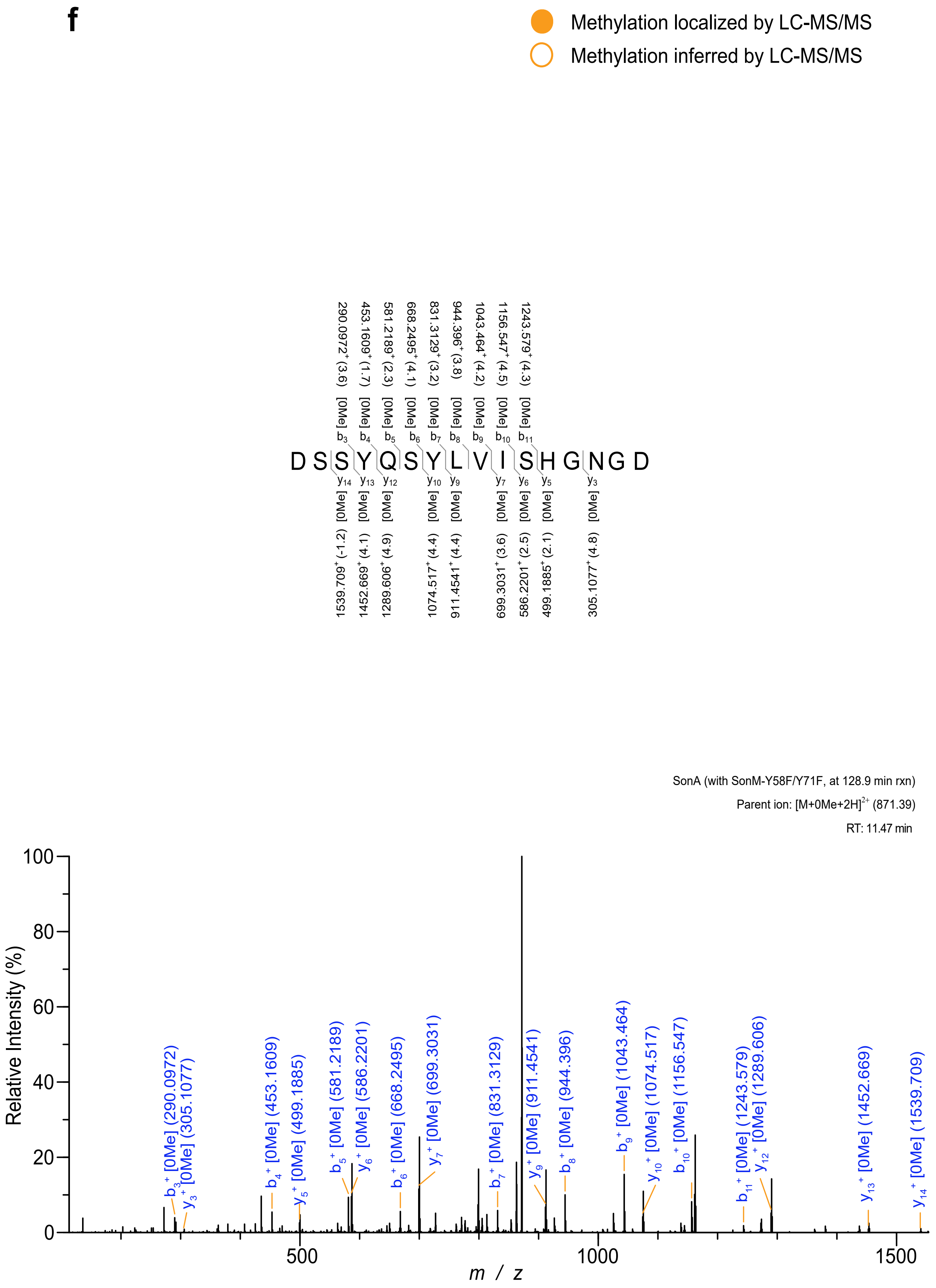

f
Methylation localized by LC-MS/MS
Methylation inferred by LC-MS/MS
D S S Y Q S Y L V I S H G N G D
SonA (with SonM-Y58F/Y71F, at 128.9 min rxn)
Parent ion: [M+0Me+2H]2+ (871.39)
RT: 11.47 min
Relative Intensity (%)
m / z

References

1. Montalbán-López, M. *et al.* New developments in RiPP discovery, enzymology and engineering. *Nat. Prod. Rep.* **37**, 919–961 (2020).

2. Song, H. *et al.* A molecular mechanism for the enzymatic methylation of nitrogen atoms within peptide bonds. *Sci. Adv.* **4**, eaat2720 (2018).

3. Ongpipattanakul, C. & Nair, S. K. Molecular basis for autocatalytic backbone *N*-methylation in RiPP natural product biosynthesis. *ACS Chem. Biol.* **13**, 2989–2999 (2018).

4. Sugimoto, K. *et al.* Crystal structure of an aromatic ring opening dioxygenase LigAB, a protocatechuate 4,5-dioxygenase, under aerobic conditions. *Structure* **7**, 953–965 (1999)

5. Sugimoto, K. *et al.* Molecular mechanism of strict substrate specificity of an extradiol dioxygenase, DesB, derived from *Sphingobium* sp. SYK-6. *PLoS One* **9**, e92249 (2014).

6. Strieter, E. R. & Andrew, T. L. Restricting the ψ torsion angle has stereoelectronic consequences on a scissile bond: An electronic structure analysis. *Biochemistry* **54**, 5748–5756 (2015).

7. Aqvist, J., Luecke, H., Quiocho, F. A. & Warshel, A. Dipoles localized at helix termini of proteins stabilize charges. *Proc. Natl. Acad. Sci. U. S. A.* **88**, 2026–30 (1991).

8. Wallace, A. C., Laskowski, R. A. & Thornton, J. M. LIGPLOT: a program to generate schematic diagrams of protein-ligand interactions. *Protein Eng.* **8**, 127–34 (1995).
