# Supplementary material for: Conformational rearrangements enable iterative backbone N-methylation in RiPP biosynthesis
Source: Nat Commun. 2021 Sep 9;12:5355. doi: 10.1038/s41467-021-25575-7 (PMC8429565; doi:10.1038/s41467-021-25575-7)
Supplement: Supplementary file 5 — Description of additional supplementary files [file 41467_2021_25575_MOESM5_ESM.docx]

Description of additional supplementary files

Title: Supplementary Data 1.

Description: Code for running kinetics simulations.

Title: Supplementary Movie 1.

Description: Animated transition between SonM— SonA-2Me—SAH and SonM—SonA-BBD—(±)SAM complexes. Hypothetical transition between the structures SonM—SonA-2Me— SAH and SonM—SonA-BBD—(±)SAM complexes and back again using the ‘morph’ function of PyMOL. The heterodimer of SonM—SonABBD—(±)SAM not bound to cofactor has large structural differences compared to wt, most notably in the top and bottom clamp orientations.

Title: Supplementary Movie 2.

Description: Animated transition between SonM— SonA-2Me—SAH and SonM-R67A—SonA-0Me—SAH complexes. Hypothetical transition between the structures SonM—SonA-2Me— SAH and SonM-R67A—SonA-0Me—SAH complexes and back again using the ‘morph’ function of PyMOL. The heterodimer of SonM— SonA-R67A—SAH has large structural differences compared to wt, most notably in helix 5 and the core peptide of SonA, as well as the top and bottom clamp orientations of SonM.
