# Supplementary material for: Conformational rearrangements enable iterative backbone N-methylation in RiPP biosynthesis
Source: Nat Commun. 2021 Sep 9;12:5355. doi: 10.1038/s41467-021-25575-7 (PMC8429565; doi:10.1038/s41467-021-25575-7)
Supplement: Supplementary file 6 — Reporting Summary [file 41467_2021_25575_MOESM6_ESM.pdf]

nature research

Corresponding author(s): Michael F. Freeman & Mikael H. Elias

Last updated by author(s): Jul 31, 2021

Reporting Summary

Nature Research wishes to improve the reproducibility of the work that we publish. This form provides structure for consistency and transparency in reporting. For further information on Nature Research policies, see our Editorial Policies and the Editorial Policy Checklist.

Statistics

For all statistical analyses, confirm that the following items are present in the figure legend, table legend, main text, or Methods section.

n/a	Confirmed	
☐	☒	The exact sample size (*n*) for each experimental group/condition, given as a discrete number and unit of measurement
☐	☒	A statement on whether measurements were taken from distinct samples or whether the same sample was measured repeatedly
☒	☐	The statistical test(s) used AND whether they are one- or two-sided<br>*Only common tests should be described solely by name; describe more complex techniques in the Methods section.*
☒	☐	A description of all covariates tested
☒	☐	A description of any assumptions or corrections, such as tests of normality and adjustment for multiple comparisons
☐	☒	A full description of the statistical parameters including central tendency (e.g. means) or other basic estimates (e.g. regression coefficient) AND variation (e.g. standard deviation) or associated estimates of uncertainty (e.g. confidence intervals)
☒	☐	For null hypothesis testing, the test statistic (e.g. *F*, *t*, *r*) with confidence intervals, effect sizes, degrees of freedom and *P* value noted<br>*Give P values as exact values whenever suitable.*
☐	☒	For Bayesian analysis, information on the choice of priors and Markov chain Monte Carlo settings
☒	☐	For hierarchical and complex designs, identification of the appropriate level for tests and full reporting of outcomes
☒	☐	Estimates of effect sizes (e.g. Cohen's *d*, Pearson's *r*), indicating how they were calculated

Our web collection on statistics for biologists contains articles on many of the points above.

Software and code

Policy information about availability of computer code

Data collection	Simulations were carried out in R version 3.6.2 using the deSolve 1.28 package. Custom code that was generated is provided with the manuscript. Other programs used in this manuscript include Geneious 2019.2, Thermo Fisher Xcalibur 3.0.63, Maxquant 1.5.3.30, GraphPad Prism 8, PyMol 2.3.3, Affinity Designer 1.8.2, XDS package version Mar 15, 2019, PHASER 2.8.3, COOT 0.8.9.2, and REFMAC 5.8.0238.
Data analysis	The fit of the models to the data were manually calculated using R^2.

For manuscripts utilizing custom algorithms or software that are central to the research but not yet described in published literature, software must be made available to editors and reviewers. We strongly encourage code deposition in a community repository (e.g. GitHub). See the Nature Research guidelines for submitting code & software for further information.

Data

Policy information about availability of data

All manuscripts must include a data availability statement. This statement should provide the following information, where applicable:

- Accession codes, unique identifiers, or web links for publicly available datasets
- A list of figures that have associated raw data
- A description of any restrictions on data availability

The mass spectrometry data and custom R code for the kinetics simulations generated in this study have been deposited in the Data Repository for the University of Minnesota (https://doi.org/10.13020/y8ry-gm18).49 Crystallographic data generated in this study are deposited in the Protein Data Bank: 7LTE [http://doi.org/10.2210/pdb7LTE/pdb] (SonM—SonA-2Me—SAH); 7LTC [http://doi.org/10.2210/pdb7LTC/pdb] (SonM—SonA-2Me); 7LTF [http://doi.org/10.2210/pdb7LTF/pdb] (SonM-Y58F—SonA-2Me); 7LTH [http://doi.org/10.2210/pdb7LTH/pdb] (SonM-Y93F—SonA-2Me); 7LTS [http://doi.org/10.2210/pdb7LTS/pdb] (SonM-R67A—SonA-0Me—SAH); 7LTR [http://doi.org/10.2210/pdb7LTR/pdb] (SonM—SonA-BBD—(±)SAM). Additional data in this study are provided in the

Supplementary Information. For Supplementary Figures 6, 14, and 17, source data are provided with this paper. All other materials and data supporting the results of this study can be requested from the corresponding authors.

Field-specific reporting

Please select the one below that is the best fit for your research. If you are not sure, read the appropriate sections before making your selection.

☒ Life sciences ☐ Behavioural & social sciences ☐ Ecological, evolutionary & environmental sciences

For a reference copy of the document with all sections, see nature.com/documents/nr-reporting-summary-flat.pdf

Life sciences study design

All studies must disclose on these points even when the disclosure is negative.

Sample size	A sample size of n=3 was used for each substrate concentration assayed with SonM wt or mutants. This sample size was chosen as 3 is statistically significant and there is precedent in the literature for performing enzyme kinetics assays with 3 technical replicates.
Data exclusions	Data points for the kinetic assays were only excluded if there was a clear malfunction in the performance of the plate reader as determined by the noise of the raw absorbance readings. Technical replicates were then re-measured for that substrate concentration with the respective enzyme.
Replication	Independent experiments were performed in vivo and in vitro for SonA and SonM wt and mutants at least twice to confirm the methylation patterns and directionality. These results were further reproduced in the kinetics modeling experiment. Three technical replicates were used for each substrate concentration assayed with SonM wt or mutants. Replicates of the entire substrate-velocity curves for wt SonM and SonA have been repeated with different purifications of SonM, SonA, and SAM at least twice as a test of reproducibility for our kinetics assay. Additionally, SonM-Y71F was purified twice and the two substrate-velocity curves for the enzymes from each purification overlaid well. All replications of experiments were successful.
Randomization	Randomization is not relevant to this study. There is no precedent or requirement for randomization in the types of biophysical and kinetics analyses performed in this manuscript . Additionally, no patient or an individual's data was collected or analyzed in this manuscript.
Blinding	Blinding is not relevant to this study. There is no precedent or requirement for blinding in the types of biophysical and kinetics analyses performed in this manuscript . Additionally, no patient or an individual's data was collected or analyzed in this manuscript.

Reporting for specific materials, systems and methods

We require information from authors about some types of materials, experimental systems and methods used in many studies. Here, indicate whether each material, system or method listed is relevant to your study. If you are not sure if a list item applies to your research, read the appropriate section before selecting a response.

Materials & experimental systems

n/a	Involved in the study	
☒	☐	Antibodies
☒	☐	Eukaryotic cell lines
☒	☐	Palaeontology and archaeology
☒	☐	Animals and other organisms
☒	☐	Human research participants
☒	☐	Clinical data
☒	☐	Dual use research of concern

Methods

n/a	Involved in the study	
☒	☐	ChIP-seq
☒	☐	Flow cytometry
☒	☐	MRI-based neuroimaging
